# Modification of subcutaneous white adipose tissue inflammation by omega-3 fatty acids is limited in human obesity-a double blind, randomised clinical trial

**DOI:** 10.1016/j.ebiom.2022.103909

**Published:** 2022-03-02

**Authors:** Helena L Fisk, Caroline E Childs, Elizabeth A Miles, Robert Ayres, Paul S Noakes, Carolina Paras-Chavez, Ondrej Kuda, Jan Kopecký, Elie Antoun, Karen A Lillycrop, Philip C Calder

**Affiliations:** a School of Human Development and Health, Faculty of Medicine, Southampton General Hospital, University of Southampton, IDS Building, MP887, Tremona Road, Southampton SO16 6YD, United Kingdom; b Medical School, University of Notre Dame Australia, Fremantle, Australia; c Institute of Physiology, Czech Academy of Sciences, Prague, Czech Republic; d School of Biological Sciences, Faculty of Environmental and Life Sciences, University of Southampton, Southampton, United Kingdom; e NIHR Southampton Biomedical Research Centre, University Hospital Southampton NHS Foundation Trust and University of Southampton, Southampton, United Kingdom

**Keywords:** Adipose tissue, Inflammation, Obesity, LC n-3 PUFA, Lipids, Immune system, 11 12-DHET, 11 12-dihydroxy-eicosatrienoic acid, 12 13-DiHOME, 12 13-dihydroxy-octadecenoic acid, 13-oxo-ODE, 13-oxo-octadecadienoic acid, 14 15-DiHETE, 14 15-dihydroxy eicosatetraenoic acid, 15-HETrE, 15-hydroxyeicosatrienoic acid, 18:1n-7, vaccenic acid, 18:1n-9, oleic acid, 18:2n-6, linoleic acid, 18:3n-3, α-linolenic acid, 20-COOH-AA, 20-COOH-arachidonic acid, 2-AG, 2-arachidonyl glycerol, 9-HpODE, 9-hydroperoxy-octadecadienoic acid, 9-HOTrE, 9-hydroxy-octadecatrienoic acid, 9-oxo-ODE, 9-oxo-octadecadienoic acid, AA, 20:4n-6, arachidonic acid, ACE2, angiotensin-converting enzyme-2, AE, adverse event, ALOX5, the gene encoding 5-lipoxygenase, ALOX15, the gene encoding 15-lipoxygenase, ALOX12, the gene encoding 12-lipoxygenase, BMI, body mass index, COX, cyclooxygenase, CYP1B1, cytochrome P450-1B1, DiHETE, dihydroxy-eicosatetraenoic acid, diHOME, dihydroxy-octadeacenoic acid, DHA, 22:6n-3, docosahexaenoic acid, DPA, 22:5n-3, docosapentaenoic acid, EPA, 20:5n-3, EpOME, epoxyoctadecaenoic acid, FA, fatty acid, FAME, fatty acid methyl ester, FDR, false discovery rate, GCGR, glucagon receptor2, GPR, G-protein coupled receptor, HDHA, hydroxy-docosahexaenoic acid, HDPA, hydroxy-docosapentaenoic acid, HETE, hydroxy-eicosatetraenoic acid, HX, hepoxilin, IL-6, interleukin-6, IL-8, interleukin-8, LC n-3 PUFA, long-chain omega-3 polyunsaturated fatty acid, LDL-C, low-density lipoprotein cholesterol, LOX, lipoxygenase, LPS, lipopolysaccharide, LT, leukotriene, LX, lipoxin, MaR, maresin, NFAT, nuclear factor of activated T-cells, NF-κB, nuclear factor kappa-light-chain-enhancer of activated B cells, OAS, 2′-5′-oligoadenylate synthetase, PD, protectin D, PG, prostaglandin, PPAR, peroxisome proliferator activated receptor, PTGS1, the gene encoding cyclooxygenase-1, PTGS2, the gene encoding cyclooxygenase-2, Rv, resolvin, SARS-CoV-2, severe acute respiratory syndrome coronavirus-2, scWAT, subcutaneous white adipose tissue, SLC27A2, the gene encoding very long-chain fatty acid Co-A synthetase, SPE, solid phase extraction, SPM, specialised pro-resolving mediator, Th, T-helper cell, TG, triglyceride, TGM2, transglutaminase-2, TNF-α, tumour necrosis factor-alpha, TREM, triggering receptor expressed on myeloid cells, TX, thromboxane, vWAT, visceral white adipose tissue, WAT, white adipose tissue

## Abstract

**Background:**

Obesity is associated with enhanced inflammation. However, investigation in human subcutaneous white adipose tissue (scWAT) is limited and the mechanisms by which inflammation occurs have not been well elucidated. Marine long chain omega-3 polyunsaturated fatty acids (LC n-3 PUFAs) have anti-inflammatory actions and may reduce scWAT inflammation.

**Methods:**

Subcutaneous white adipose tissue (scWAT) biopsies were collected from individuals living with obesity (n=45) and normal weight individuals (n=39) prior to and following a 12-week intervention with either 3 g/day of a fish oil concentrate (providing 1.1 g eicosapentaenoic acid (EPA) + 0.8 g docosahexaenoic acid (DHA)) or 3 g/day of corn oil. ScWAT fatty acid, oxylipin, and transcriptome profiles were assessed by gas chromatography, ultra-pure liquid chromatography tandem mass spectrometry, RNA sequencing and qRT-PCR, respectively.

**Findings:**

Obesity was associated with greater scWAT inflammation demonstrated by lower concentrations of specialised pro-resolving mediators (SPMs) and hydroxy-DHA metabolites and an altered transcriptome with differential expression of genes involved in LC n-3 PUFA activation, oxylipin synthesis, inflammation, and immune response. Intervention with LC n-3 PUFAs increased their respective metabolites including the SPM precursor 14-hydroxy-DHA in normal weight individuals and decreased arachidonic acid derived metabolites and expression of genes involved in immune and inflammatory response with a greater effect in normal weight individuals.

**Interpretation:**

Downregulated expression of genes responsible for fatty acid activation and metabolism may contribute to an inflammatory oxylipin profile and limit the effects of LC n-3 PUFAs in obesity. There may be a need for personalised LC n-3 PUFA supplementation based on obesity status.

**Funding:**

European Commission Seventh Framework Programme (Grant Number 244995) and Czech Academy of Sciences (Lumina quaeruntur LQ200111901).

**Research in context**

**Evidence before this study**

Obesity increases the risk of developing conditions such as hyperlipidaemia, diabetes, and cardiovascular disease, and has negative consequences on the immune system. Inflammation is part of the immune response; however, chronic inflammation accompanying obesity results in dysregulated responses linking adipose tissue and the immune system. The pathogenesis of adipose inflammation in humans is understudied; current literature focuses on circulating markers of inflammation and metabolic health is often overlooked when attempting to elucidate the link between obesity and inflammation. Marine derived long chain omega-3 polyunsaturated fatty acids (LC n-3 PUFAs) are anti-inflammatory but their effects on obesity-associated scWAT inflammation are understudied. Comprehensive investigation of multiple aspects of tissue physiology and inflammation in human scWAT in obesity in comparison to normal weight individuals and in response to LC n-3 PUFA is lacking.

**Added value of this study**

Here we report a comprehensive evaluation of scWAT inflammation in human obesity prior to metabolic complications. We identify and describe novel alterations to FA composition and subsequent oxylipin profile, including decreased levels of specialised pro-resolving mediators (SPMs), increased expression of genes associated with inflammatory and immune signalling, including SARS CoV-2 pathogenesis, and altered response to the effects of LC n-3 PUFAs on these parameters. LC n-3 PUFA intervention decreased pro-inflammatory oxylipin levels and inflammatory and immune gene expression in scWAT to a greater extent in normal weight individuals in comparison to those living with obesity. In addition, LC n-3 PUFA intervention increased EPA and DHA metabolites including the precursor to the SPM maresin-1, 14-hydroxy-DHA, in normal weight individuals only. We, for the first time, identify differences in the expression of genes involved in fatty acid handling in individuals living with obesity, including *SLC27A2*, which is required for the metabolism of DHA to further bioactive signalling molecules. We describe this as one mechanism by which the effects of DHA are impaired in individuals living with obesity, in addition to potential differences in LC n-3 PUFA membrane incorporation and contribution following intervention.

**Implications of all the available evidence**

Our findings describe for the first time, alteration to the scWAT oxylipin and transcriptome profile in human obesity, providing insight into the progression of inflammation through oxylipin signalling and gene expression which links scWAT, the immune system, and whole-body homeostasis. Furthermore, our findings highlight the importance of metabolic health in the progression of obesity-associated inflammation. LC n-3 PUFAs have been shown to have anti-inflammatory actions in humans but we describe for the first time, anti-inflammatory actions of these lipids in scWAT through modulation of oxylipin signalling and immune and inflammatory gene expression. Furthermore, we describe altered response to the effects of LC n-3 PUFAs in human obesity (compared to normal weight). These findings provide novel insights into altered inflammatory signalling in scWAT and responses to LC n-3 PUFA intervention in obesity, potential mechanisms by which these occur, and links negative effects of obesity on the immune system with scWAT pathophysiology.

Alt-text: Unlabelled box

## Introduction

Obesity affects over 13% of the adult population worldwide^1^ and increases the risk of developing conditions such as type-2 diabetes and cardiovascular disease.^2^, ^3^, ^4^ Furthermore, the negative consequences of obesity on the immune system have been recently highlighted during the severe acute respiratory syndrome coronavirus-2 (SARS-CoV-2) pandemic.^5^, ^6^, ^7^ Inflammation is a vital part of the immune response; however, obesity is accompanied by a state of chronic low-grade inflammation in which responses are dysregulated.^8^ This obesity-associated chronic inflammation links adipose tissue and the immune system.

The fatty acid (FA) composition of cells resident in adipose tissue influences both the innate and the adaptive immune system and inflammatory signalling in the tissue through a range of diverse mechanisms.^9^ One mechanism by which this occurs is metabolism of polyunsaturated FAs (PUFAs) into bioactive lipid signalling molecules termed lipid mediators which include oxylipins and endocannabinoids.^10^ Oxylipins can be synthesised and secreted by adipose resident cells, including both immune cells and adipocytes.^10^^,^^11^ Altered production of, and sensitivity to, such oxylipins contributes to the adipose tissue inflammation that occurs in obesity.^10^^,^^12^ We previously reported altered endocannabinoid concentrations in subcutaneous WAT (scWAT) in individuals with obesity in comparison to normal weight individuals.^13^

Oxylipins elicit a range of both pro- and anti-inflammatory actions. Pro-inflammatory actions of oxylipins synthesised from the omega-6 PUFA arachidonic acid (AA) include promoting infiltration and activation of immune cells,^14^, ^15^, ^16^ altering lipid metabolism,^17^ and promoting WAT expansion and remodelling.^18^ The two most bioactive long chain omega-3 PUFAs (LC n-3 PUFAs), eicosapentaenoic acid (EPA) and docosahexaenoic acid (DHA), can be oxidised to form a group of oxylipins termed specialised pro-resolving mediators (SPMs).^19^^,^^20^ These have anti-inflammatory actions and have been reported to regulate the infiltration of leukocytes, block interleukin (IL)-1 induced activation of nuclear factor kappa-light-chain-enhancer of activated B cells (NF-κB) and decrease the expression of pro-inflammatory cytokines.^21^, ^22^, ^23^, ^24^ We previously reported EPA and DHA to increase the concentrations of two endocannabinoids, eicosapentaenoyl ethanolamide and docosahexaenoyl ethanolamide, in scWAT in normal weight individuals but that this effect of LC n-3 PUFAs is impaired in individuals with obesity.^13^ It has been suggested that deficiency of SPMs in obesity may contribute to dampened immunity and poor response to viral infections^25^ but reports of altered concentrations of SPMs in WAT in human obesity are limited, as highlighted by Pal et al.^25^^,^^26^ and Han et al.^27^

In addition to potentially influencing oxylipin formation, EPA and DHA elicit their actions via altering the activity of transcription factors to modulate inflammation and other processes in WAT.^28^^,^^29^ Furthermore, EPA and DHA can bind to receptors such as peroxisome proliferator activated receptors (PPARs)^30^ to regulate the expression of genes associated with lipid accumulation in WAT,^31^^,^^32^ and to G-protein coupled receptor (GPR)-120 to regulate inflammation and insulin sensitivity.^33^ Therefore, EPA and DHA have the potential to reduce inflammation in adipose tissue but investigation of this in human scWAT is limited, as highlighted in a recent review by Dewhurst-Trigg et al.^34^

Obesity is accompanied by several pathophysiological changes in adipose tissue, but the mechanisms behind these have not been well elucidated with reports predominantly focussing on circulating markers of inflammation. In addition, the metabolic health of individuals living with obesity is often overlooked when trying to understand the link between obesity and adipose tissue inflammation which can also be said for assessing the effects of LC n-3 PUFAs.^35^, ^36^, ^37^ The earlier stages of obesity in which metabolic complications have not yet manifested, may offer a window for therapeutic intervention in that the adipose tissue may have a greater degree of normal function and be responsive to manipulation, for example by different PUFAs. Further to this, comprehensive investigation of scWAT assessing multiple aspects of tissue physiology and inflammation in obesity in comparison to normal weight individuals and in response to LC n-3 PUFAs is lacking.^34^

In this study we combine whole tissue FA, oxylipin, and transcriptome profiling to investigate obesity associated scWAT inflammation, to identify potential mechanisms by which this occurs in humans, and to assess responses to LC n-3 PUFA intervention.

## Methods

### Participants

50 healthy normal weight individuals (BMI 18.5 to 25 kg/m^2^) and 50 individuals living with obesity (BMI 30 to 40 kg/m^2^, waist circumference ≥ 94 cm males and ≥ 80 cm females) aged 18-65 years were recruited into a double blind placebo (comparator oil) controlled trial (Supplemental Figure 1) at the University of Southampton, UK. Prior to scWAT collection at week-0, 16 individuals withdrew leaving 39 normal weight individuals and 45 individuals living with obesity. The House of Commons Library Obesity Statistics briefing paper for 2021 estimates 28% of adults (age 18 years and above) in the UK to be obese. The age group most likely to be overweight or obese is age 65–74 years and the prevalence of overweight and obesity is above 70% for all age groups aged 45 years and above.^38^ The cohort recruited as part of the current study is reflective of the wider UK population of adults living with obesity without diagnosed diabetes and or other diagnosed metabolic complications. Individuals outside the defined BMI, age or waist circumference categories, diagnosed with metabolic disease (e.g. diabetes, cardiovascular disease) or chronic gastrointestinal problems (e.g. inflammatory bowel disease, celiac disease, and cancer), using prescribed medicine to control blood lipids, blood pressure or inflammation, consuming more than one serving of oily fish per week (140 g cooked), taking fish oil or other oil supplements, who were pregnant or planning to become pregnant during the study period, or were participating in another clinical trial were not eligible for the study.

### Study design

Fasted blood and an abdominal scWAT biopsy (∼1 g) were collected at study entry (week-0) and following 12 weeks intervention (week-12) during which participants were randomised to consume either 3 g of a fish oil concentrate (EPAX6000 (Epax Norway AS, Alesund, Norway) providing 1.1 g EPA + 0.8 g DHA) or 3 g of corn oil (providing 1.65 g linoleic acid and 0.81 g oleic acid) per day (Supplemental Figure 1). Full composition of the intervention oils is detailed in Supplemental Table 1; both oils were provided in one g softgel capsules. Blinding, randomization, and supplement packaging were completed by the Research Pharmacy at Southampton General Hospital, Southampton, United Kingdom, by individuals independent of the researchers involved in the study. Treatment group blinding was maintained until completion of statistical analysis of all data.

### Adverse events

Among the 39 normal weight participants, there were 32 adverse events (AEs) during the 12-week intervention period; of these 21 were not related to any study procedure or the oil treatments, 1 was possibly related to oil treatment (headache), and 10 were certainly related to study procedures (all related to bruising, bleeding, pain or mild infection at the site of the wound induced to collect one of the two adipose tissue biopsies). Among the 45 participants living with obesity, there were 30 AEs during the 12 week intervention period; of these 19 were not related to any study procedure or the oil treatments, 2 were possibly related to oil treatment (indigestion, headache), 1 was possibly related to a study procedure (arm pain in the area of cannulation for blood collection) and 8 were certainly related to study procedures (all related to bruising, bleeding, pain or mild infection at the site of the wound induced to collect one of the two adipose tissue biopsies). None of these participants withdrew from the study as a result of the AE.

### Sample preparation

Abdominal scWAT biopsies were collected by surgical removal and stored as previously described.^13^ ∼5 mL of heparinised blood was collected and stored on ice. Plasma was prepared by centrifugation (1900 × g, 10 min, room temperature) and stored at −80°C until analysis. Plasma triglyceride (TG), cholesterol, high density lipoprotein cholesterol (HDL-C), non-esterified fatty acid (NEFA), insulin and glucose concentrations were measured as previously described.^13^ Low density lipoprotein cholesterol (LDL-C) concentrations were calculated using the Friedewald formula. HOMA2-IR was calculated as follows: (((insulin mmol/L) x (glucose IU/L)) / 22.5) corrected for variations in hepatic and peripheral glucose resistance, increases in insulin secretion curve for plasma glucose concentrations above 10 mmol/L, and the contribution of circulating proinsulin (University of Oxford, Oxford, UK).

### Anthropometry

Height was measured by a Seca stadiometer (Seca, Hamburg, Germany), weight and body composition measurements were made using bioelectrical impedance apparatus (TANITA BC-418), and waist and hip circumference measurements were made using a tape measure as previously described.^13^

### Fatty acid composition

Total lipids were extracted from frozen scWAT, red blood cells and plasma and were processed to obtain FA methyl esters (FAMEs) as previously described.^13^^,^^39^ FAMEs were separated by gas chromatography on a BPX-70 fused silica capillary column (30 m x 0.2 mm x 0.25 µm; manufactured by SGE) in a HP6890 gas chromatograph fitted with a flame ionisation detector. Run conditions were as described elsewhere.^39^

### Oxylipin analysis

FA metabolites were extracted from ∼100 mg of liquid N_2_ frozen scWAT as previously described.^13^ FA metabolites were separated on a Thermo Dionex Ultimate 3000 RSLC (Dionex Corporation, Sunnyvale, CA) liquid chromatograph fitted with a Kinetex® UPLC column 1.7 µm particle size, C18 stationary phase, 100 A pore size, 150 × 2.1 mm (Phenomenex, Torrance, CA) with mobile phase composition A = 70% H_2_O, 30% acetonitrile and 0.02% acetic acid; B = 50% acetonitrile and 50% isopropanol, and identified on an AB SCIEX SelexION QTRAP 5500 (AB SCIEX, Framingham, MA) triple quadrupole mass spectrometer selecting for electrospray in both positive and negative modes.^40^ Deuterated internal standards were used to orientate the resulting UPLC-MS outputs and identify correct FA metabolite peaks using Analyst software (version 1.6.2 2013, AB SCIEX, Framingham, MA). The limit of detection was ≥ 0.1 pg for all metabolites. Calibration curves were created for the quantification of FA metabolites; however, availability of standards was limited so data are expressed as absolute concentration (pg/mg tissue) for those that could be quantified against calibration curve data, and as proportion (% of total metabolites measured) for those without calibration curve data.

### Gene expression

#### RNA sequencing

RNA was isolated from scWAT and stored in RNAlater® using the RNeasy lipid tissue mini kit™ (QIAGEN, Hilden, Germany). The kit protocol was carried out twice to yield RNA from ∼150 mg of scWAT in total from each participant, yielding an average of 2.47 µg of total RNA determined by Nanodrop 2000 (Thermo Scientific, Waltham, MA). A subset of 40 samples (20 paired week-0 and week-12 normal weight, 20 paired week-0 and week-12 obese, best matched for age and sex) were extracted to obtain RNA with an RNA integrity (RIN) score of 6.5 to be sequenced. Sequencing was performed on a Hiseq2000 platform with 5 samples per lane in a total of 8 lanes (SE50) with a total of 20 million reads. RNA-Seq reads were aligned to the hg38.0 reference genome using TopHat^41^ (open source, Johns Hopkins University, Center for Computational Biology, Baltimore, MD) and a read count table produced using HTSeq^42^ (open source, Huber group, Heidelberg, Germany). The read counts were normalised in EdgeR^43^ (open source, Bioconductor.org). The normalised counts per million (CPM) were used to evaluate gene expression. The results from this subset were validated via qRT-PCR of RNA extracted from the whole cohort which showed the subset to be representative of the whole cohort.

#### Real-time quantitative PCR

Reverse-transcription was performed to obtain cDNA using the GoScript Reverse Transcription System (Promega, Southampton, UK) and gene expression was quantified with RT-qPCR using Applied Biosystems 7500 qRT-PCR (Applied Biosystems, Waltham, MA) using 4 ng cDNA per 20 μl reaction with QuantiNova Probe PCR kit (QIAGEN, Hilden, Germany) and custom double dye taqman style primers (Primer Design, Southampton, UK). Primer sequences for genes validated by qRT-PCR and housekeeping genes are detailed in Supplemental Table 2. RT-qPCR data were analysed using the comparative ΔΔCt approach against three housekeeping genes to obtain fold change values.

### COX-2 activity

Total protein was extracted from ∼50 mg scWAT homogenised in lysis buffer (1% NP-40 in PBS + protease inhibitor cocktail (1:200)). Supernatant was collected following centrifugation and the protein concentration determined using the Pierce™ BCA Protein Assay Kit (Fisher Scientific, Loughborough, UK) following the manufacturer's instructions. Cyclooxygenase (COX)-2 activity was measured using the COX Activity Assay kit (Abcam, Cambridge, UK) according to the manufacturer's instructions and the plate read in loop mode at Ex/Em 520/ 580–640nm for 30 min. Delta RFU (30 min data–0 min data) data were calculated against a standard curve and adjusted to mg protein used to give activity in µU/mg.

### Statistics

Sample size was calculated considering the typical distribution and expected response of circulating cytokines, not reported here (20% decrease in IL-6 following fish oil intervention) and participant drop out of 20%.^13^ A sample size of 25 participants per group (BMI and treatment subgroup) was determined to be able to detect changes in circulating cytokines at > 80% power and a 5% level of significance with consideration for 20% loss.^13^ Not all data were normally distributed, and some could not be normalised with log_10_ transformation. Appropriate non-parametric tests were performed on these data, and non-normal data are reported as median and interquartile range (IQR).

The limit of detection was ≥ 0.1 pg for all oxylipins; signals were determined to be genuine above this level and if 30% greater compared to background signals. Oxylipin data were evaluated by Mann-Whitney U test to identify differences in FA metabolite composition of individuals living with obesity in comparison to normal weight individuals, and by Wilcoxon test with data split by BMI and selected by treatment to compare paired post intervention data (week-12) with study entry (week-0).

RNA-sequencing data were analysed by pair-wise comparison in EdgeR (open source, Bioconductor.org) to obtain the false discovery rate, fold change and significance of the data. Upper limits for FDR and *P* value (≤0.05, both) were applied to the data. The significance of RT-qPCR fold change data was evaluated by Mann Whitney-U test to identify differences in gene expression of individuals living with obesity in comparison to normal weight individuals at study entry, and by Wilcoxon test to compare paired post intervention data with study entry data.

Participant characteristic data were normally distributed and data from normal weight individuals and individuals living with obesity were compared using univariate general linear model analysis. Oxylipin data were not normally distributed and subsequent analysis of correlations between oxylipin data, HOMA2-IR, and expression of enzymes encoding COX and LOX were assessed by Spearman's correlation.

Data can be openly accessed in GEO under accession code GSE162653.

For all data, significance level was set as ≤0.050.

### Ethics

All procedures involving human subjects were approved by the National Research Ethics Service South Central–Berkshire Research Ethics Committee (submission no. 11/SC/0384). The trial was conducted according to the principles of the Declaration of Helsinki, and all participants gave written informed consent prior to enrolment. The study is registered at www.isrctn.com (study ID: ISRCTN96712688).

### Role of funding source

The research was funded by the European Commission Seventh Framework Programme (Grant Number 244995) and the Czech Academy of Sciences (Lumina quaeruntur LQ200111901). The funding sources had no role in study design, data collection, data analyses, interpretation, or writing of the manuscript.

## Results

### Anthropometric and metabolic characteristics of participants

To evaluate obesity-associated inflammation in human scWAT, we recruited 100 individuals, characterising them as normal weight (n = 50) or living with obesity (n = 50) based upon their BMI and waist circumference. Diagnosed metabolic or inflammatory conditions were exclusion criteria for recruitment to the study but to gain further insight into the metabolic health of individuals recruited to the study, a range of anthropometric and metabolic parameters were measured. Sixteen individuals withdrew from the study prior to their first clinical visit resulting in scWAT biopsies being collected from 39 normal weight individuals and 45 individuals living with obesity. The anthropometric and metabolic characteristics of these individuals are detailed in Table 1. Individuals living with obesity had significantly greater BMI, % body fat, body fat mass (kg), waist and hip circumference, and higher blood concentrations of TG, total cholesterol, LDL-C, glucose, and insulin in comparison with normal weight individuals (Table 1). Despite individuals living with obesity having significantly higher blood concentrations of TG, glucose, and insulin, and higher HOMA2-IR scores, these were generally still within the clinically defined ‘normal range’ (Table 1). As individuals living with obesity recruited to the study did not exhibit hypertriglyceridemia and had HOMA2-IR scores within the normal range, they were defined as living with obesity in which metabolic syndrome is yet to manifest.

**Table 1:** Anthropometric and metabolic characteristics in normal weight and individuals living with obesity reproduced from Fisk et al.^13^

	1Normal weight (n = 39)	1Obese (n = 45)	2*P*	Normal range
Sex M/F	10 / 28	12 / 33		
Age	31.68 ± 14.79	44.55 ± 12.14	≤ 0.001	
BMI (kg/m^2^)	22.25 ± 1.79	34.80 ± 2.87	≤ 0.001	
Waist (cm)	75.41 ± 7.00	108.42 ± 11.87	≤ 0.001	
Hip (cm)	92.92 ± 5.23	117.74 ± 8.21	≤ 0.001	
Body fat (%)	22.63 ± 7.43	41.52 ± 6.84	≤ 0.001	
Body fat mass (kg)	13.73 ± 4.40	40.45 ± 7.67	≤ 0.001	
Lean mass (kg)	48.25 ± 10.40	57.53 ± 12.02	0.001	
TG (mmol/L)	0.79 ± 0.29	1.32 ± 0.70	≤ 0.001	< 1.7 mmol/L
NEFAs (mmol/L)	0.48 ± 0.21	0.60 ± 0.22	0.245	< 0.72 mmol/L
TC (mmol/L)	4.42 ± 1.04	5.24 ± 0.92	≤ 0.001	< 5.0 mmol/L
HDL-C (mmol/L)	1.57 ± 0.39	1.49 ± 0.37	0.295	> 1.0 mmol/L
LDL-C (mmol/L)	2.69 ± 0.90	3.49 ± 0.78	≤ 0.001	< 3.0 mmol/L
Glucose (mmol/L)	4.77 ± 0.42	5.6 ± 1.05	≤ 0.001	<7.0 mmol/L
Insulin µIU/L	5.48 ± 2.74	13.03 ± 6.69	≤ 0.001	2.6-24.9 µIU/L
3HOMA2-IR	0.73 ± 0.35	1.64 ± 0.79	≤ 0.001	< 1.9
4Adipose-IR	2843 ± 2097	7110 ± 3320	≤ 0.001	

1 Mean ± SD.

2 *P* obtained from univariate general linear model analysis by comparison of obese and normal weight data.

3 HOMA2-IR = HOMA2-IR = (((insulin mmol/L) x (glucose IU/L)) / 22.5) corrected for variations in hepatic and peripheral glucose resistance, increases in insulin secretion curve for plasma glucose concentrations above 10 mmol/L, and the contribution of circulation proinsulin.

4 Adipose-IR = (NEFAs mmol/L) x (insulin µIU/L).

### Obesity is associated with a dysregulated oxylipin profile indicative of enhanced inflammation and inhibited resolution

**Figure 1:**
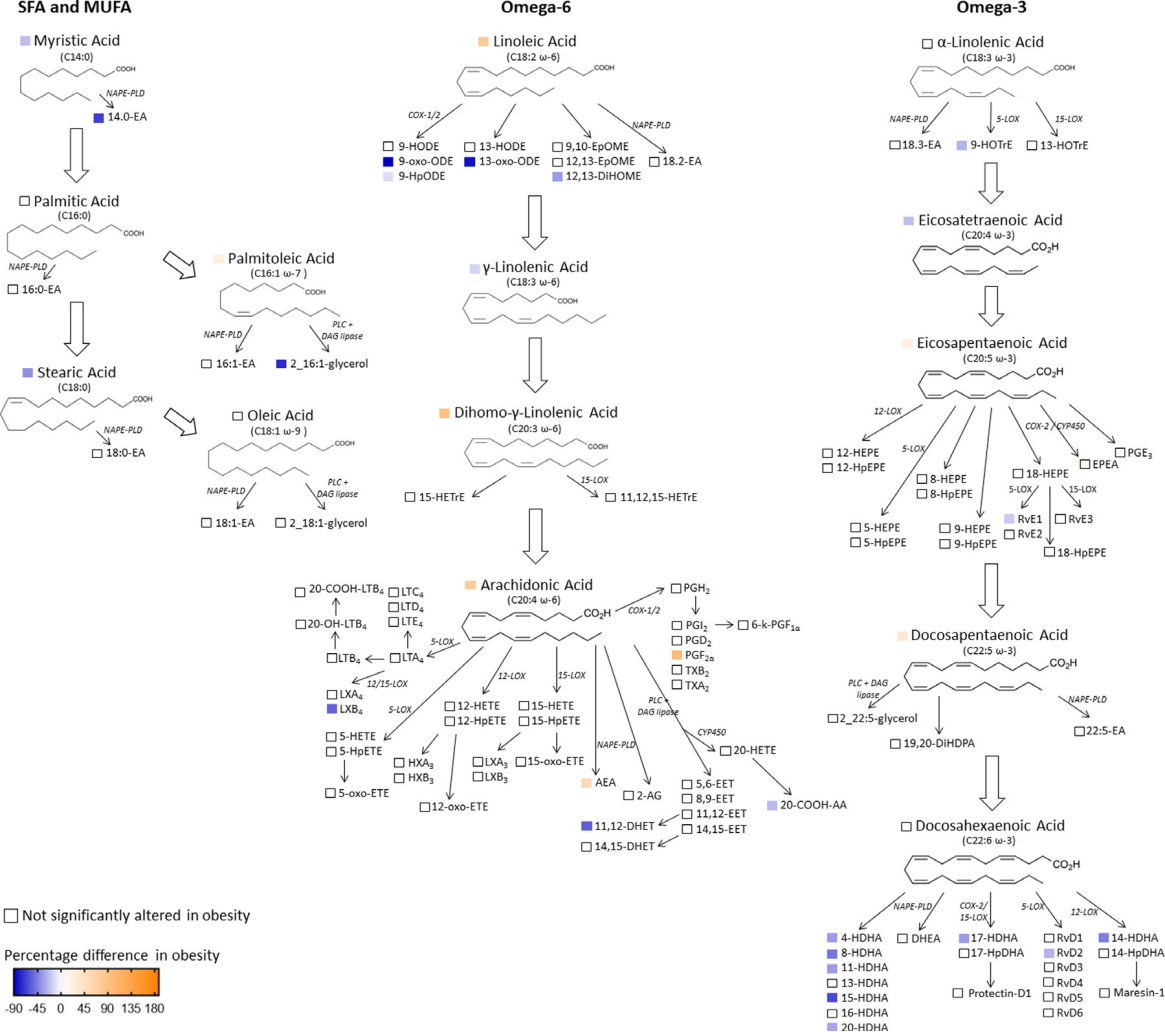
Fatty acids and fatty acid-derived mediators in scWAT at study entry (week-0) in individuals living with obesity (n=45) in comparison to normal weight individuals (n=39). Data are shown according to the significance (*P*) of the proportional difference at study entry ((normal weight week-0) – (obesity week-0)). Significance is defined as *P* < 0.05, Mann-Whitney U test.

Profiling of human scWAT FA composition revealed significantly altered proportions of several PUFAs in individuals living with obesity in comparison to normal weight individuals at week-0. Significantly higher proportions of the n-6 PUFAs dihomo-gamma-linolenic acid (20:3n-6) and AA (20:4n-6), and the n-3 PUFAs EPA (20:5n-3) and docosapentaenoic acid (DPA; 22:5n-3), as well as significantly lower proportions of the n-3 PUFAs alpha-linolenic acid (18:3n-3) and eicosatetraenoic acid (20:4n-3) were observed in individuals living with obesity (Figure 1). The proportions of EPA and DHA were significantly correlated with markers of insulin resistance. The proportion of EPA was positively correlated with adipose-IR (ρ 0.248, *P* = 0.043) and the proportion of DPA with HOMA2-IR and adipose-IR (ρ 0.258, *P* = 0.038, and ρ 0.342, *P* = 0.005 respectively, Spearman's rank order correlation, data not shown). Comparison of the full FA composition of scWAT in individuals living with obesity in comparison to normal weight individuals from this cohort is previously described.^13^

Oxylipin profiling of whole scWAT was employed to assess inflammatory lipid signalling in obesity. 111 FA metabolites were identified in human scWAT; 33 of these were significantly altered in individuals living with obesity in comparison to normal weight individuals (Figure 1). Dysregulation of oxylipins derived from n-3 and n-6 PUFAs was observed in individuals living with obesity at study entry. There were lower proportions of 9-hydroperoxy-octadecadienoic acid (9-HpODE), 9-oxo-octadecadienoic acid (9-oxo-ODE), 13-oxo-octadecadienoic acid (13-oxo-ODE), 12,13-dihydroxy-octadecenoic acid (12,13-DiHOME), 20-COOH-arachidonic acid (20-COOH-AA), 11,12-dihydroxy-eicosatrienoic acid (11,12-DHET), u-leukotriene (LT) D_4_, lipoxin B_4_ (LXB_4_), hepoxilin A_3_ (HXA_3_), 9-hydroxy-octadecatrienoic acid (9-HOTrE), resolvin (Rv) E1, 8-HDHA, 14-HDHA, 15-HDHA, 17-HDHA, 20-HDHA, and RvD2, and lower concentrations of 4-HDHA and 11-HDHA in individuals living with obesity (Figure 1). In addition, there were lower proportions of 14:0-EA and 16:1-glycerol and higher concentrations of prostaglandin (PG) F_2α_ (Figure 1) in individuals living in obesity.

Several oxylipins were significantly correlated with HOMA2-IR and a number of these were dysregulated in individuals living with obesity. 9-HpODE, 9-oxo-ODE, 13-oxo-ODE, 6-keto-PGF_2α_, 11,12-DHET, 14,15-dihydroxy eicosatetraenoic acid (14,15-DiHETE), 17,18-DiHETE, HXA_3_, 12-hydroxyeicosatetraenoic acid (12-HETE), 12-HEPE, 4-HDHA, 8-HDHA, 11-HDHA, 13-HDHA, 14-HDHA, 17-HDHA and 20-HDHA were all negatively correlated with HOMA2-IR indicating lower proportions of these oxylipins with increasing insulin resistance (Table 2).

**Table 2:** Correlations between oxylipins in scWAT and HOMA2-IR scores.

Oxylipin	ρ^1^	*P*^1^
9-HpODE	-0.253	0.038
9-oxo-ODE	-0.348	0.004
13-oxo-ODE	-0.377	0.002
6-keto-PGF_2α_	-0.260	0.034
11,12-DHET	-0.303	0.013
14,15-DiHETE	-0.286	0.019
17,18-DiHETE	-0.313	0.010
HXA_3_	-0.320	<0.001
12-HETE	-0.306	0.012
12-HEPE	-0.240	0.051
4-HDHA	-0.252	0.004
8-HDHA	-0.247	0.044
11-HDHA	-0.315	0.009
13-HDHA	-0.369	0.002
14-HDHA	-0.256	0.036
17-HDHA	-0.276	0.024
20-HDHA	-0.343	0.005

1 Coefficient and *P* value obtained using Spearman's rank order correlation, significance is deemed *P* < 0.05.

### Role of enzymes involved in FA metabolism in the oxylipin profile changes seen in obesity

**Figure 2:**
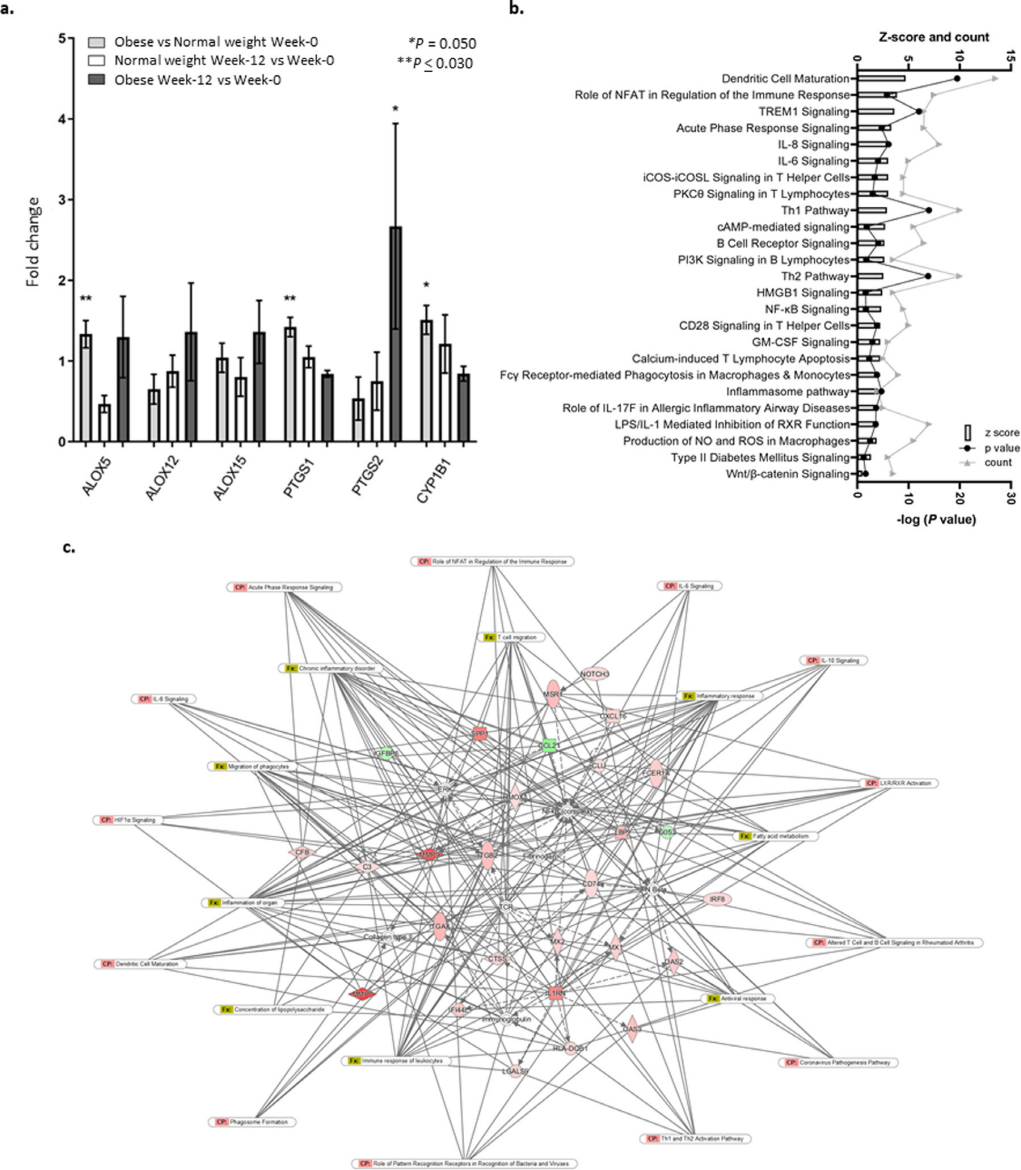
**a.** Fold difference in gene expression in scWAT from individuals living with obesity (n=39) compared with normal weight individuals (n=28) at study entry (week-0) (univariate general linear model) and fold change in both groups following 12-week fish oil intervention (week-12 compared with week-0, paired samples T-test). Mean (± SEM) * *P* = 0.050, **P ≤ 0.030. **b.** Histogram of 25 canonical pathways associated with differential gene expression in scWAT from individuals living with obesity. NFAT, nuclear factor of activated T cells; TREM1, triggering receptor expressed on myeloid cells. **c.** Network of differentially expressed genes in scWAT from individuals living with obesity compared with normal weight individuals and association with canonical pathways (CP) and disease & function (Fx) pathways. Red = upregulated, green = downregulated. 2 x FC, FDR 0.05, *P* ≤ 0.05.

The expression of genes encoding cytochrome P450 (CYP-450), cyclooxygenase (COX) and lipoxygenase (LOX) enzymes, which are involved in the oxidation of PUFAs to form oxylipins, was investigated to assess their contribution to the differences in the oxylipin profile observed at study entry between normal weight individuals and those living with obesity. The mRNA expression of a cytochrome P450 gene *CYP1B1, ALOX5* (which encodes 5-LOX), and *PTGS1* (which encodes COX-1) was upregulated in individuals living with obesity (Figure 2a). *ALOX5* mRNA expression was positively correlated with body % and kg of body fat (ρ = 0.423, *P* <0.001 and ρ = 0.457, *P* <0.001 respectively, Spearman's rank order correlation, data not shown) and *ALOX12* mRNA expression was negatively correlated with % body fat (ρ = -0.263, *P* = 0.036, Spearman's rank order correlation, data not shown).

Correlations between the expression of COX, LOX, and CYP genes and proportions of scWAT oxylipins were assessed. In normal weight individuals, the proportion of 17-HDHA positively correlated with the expression of *ALOX15*, and the proportion of 11-HDHA positively correlated with *ALOX15* and *CYCP1B1* expression (Table 3). In individuals living with obesity, 13-HODE was positively correlated with the expression of *PTGS2*, and PGD_3_ was positively correlated with *PTGS1* and negatively correlated with *PTGS2* (Table 3). In individuals living with obesity, several oxylipins were negatively correlated with the enzyme involved in their synthesis which may suggest dysregulation in the metabolism of FAs to signalling molecules in obesity. LXA_5_, 15-HEPE, and RvE3 were negatively correlated with *ALOX15* expression (Table 3). Furthermore, the proportions of DHA-derived oxylipins, RvE2, RvD1, RvD2, 4-HDHA, and 20-HDHA were negatively correlated with expression of *ALOX15* which is not directly involved in the metabolism of DHA to these metabolites (Table 3).

**Table 3:** Correlations between expression of enzymes encoding LOX and COX enzymes and oxylipin proportions in scWAT from normal weight individuals and individuals living with obesity.

Oxylipin	Enzyme encoding	Normal weight		Obese	
		ρ^1^	*P*^1^	ρ^1^	*P*^1^
13-HODE	COX-2	0.144	0.511	0.331	0.039
PGD_3_	COX-1	0.045	0.838	0.640	0.046
	COX-2	-0.628	0.070	-0.349	0.029
LXA_5_	15-LOX	-0.044	0.841	-0.397	0.012
15-HEPE	15-LOX	0.372	0.080	-0.346	0.001
RvE2	15-LOX	0.298	0.167	-0.365	0.022
RvE3	15-LOX	-0.022	0.920	-0.442	0.005
RvD1	15-LOX	-0.001	0.996	-0.345	0.029
RvD2	15-LOX	0.263	0.226	-0.494	0.001
4-HDHA	15-LOX	0.103	0.641	-0.434	0.006
	COX-2	-0.222	0.308	-0.381	0.017
11-HDHA	15-LOX	0.465	0.026	-0.132	0.424
17-HDHA	15-LOX	0.510	0.013	-0.290	0.073
20-HDHA	15-LOX	0.370	0.082	-0.331	0.034
	COX-2	-0.046	0.833	-0.331	0.054

1 Coefficient and *P* value obtained using Spearman's rank order correlation, significance is deemed *P* < 0.05. Normal weight individuals (n=39), individuals living with obesity (n=45).

### Obesity is associated with dysregulated expression of inflammatory and immune response related genes

Sequencing of RNA extracted from human scWAT identified 4461 differentially expressed genes in individuals living with obesity compared with normal weight individuals; 798 genes showed at least a two-fold significant difference in expression (*P* ≤0.05, FDR ≤0.05), with 622 of these upregulated and 176 downregulated in obesity (Supplemental Table 3). Examination of the global difference between the two phenotypes was conducted by principal component analysis for each sample at study entry and its paired sample following oil treatment and identified samples to cluster by obesity status (Supplemental Figure 2). The top 10 upregulated genes were *EGLF6, MMP7, CCL22, MMP9, DCSTAMP, URAD, LINC01010, AADACL3, CHIT1*, and *SPP1* (*P* ≤ 0.001, Supplemental Table 3). The top 10 downregulated genes were *SLC27A2, RORB, SPX, CA3, CECR, WDR860AS1, KCNU1, ASPG, BMP3* and *RASSF6* (Supplemental Table 3).

To understand the functional significance of the changes, Ingenuity pathway analysis (Qiagen, Hilden, Germany) was run to identify pathways and networks enriched amongst the differentially expressed genes. The majority of differentially expressed genes were associated with inflammatory and immune responses and the results indicated an upregulation of these processes in the scWAT of individuals living with obesity compared to those of normal weight. Twenty-five of the most enriched inflammatory-associated canonical pathways in individuals living with obesity are depicted in Figure 2b. Upregulated genes in individuals living with obesity were enriched amongst dendritic cell maturation, nuclear factor of activated T-cells (NFAT) regulation of immune response, inflammatory signalling including IL-6 and IL-8 signalling, triggering receptor expressed on myeloid cells (TREM), B- and T-cell signalling, production of nitric oxide and reactive oxygen species, and NF-κB and inflammasome pathways (Figure 2b).

Several genes that were differentially expressed in scWAT from individuals living with obesity were observed to interact with each other and were associated with key inflammatory and homeostatic processes in scWAT including immune cell and cytokine signalling, immune cell infiltration and development and hypoxia (Figure 2c). In addition to immune and inflammatory processes, ∼20% of differentially expressed genes in individuals living with obesity were associated with lipid and carbohydrate metabolism and signalling which may contribute to an upregulation in the type 2 diabetes signalling pathway (Figure 2b). A small number of these genes can also be seen to interact with inflammatory processes in the network depicted in Figure 2c. These pathways include cytokine signalling (IL-6, -8, and -10), acute phase response signalling, T-helper cell (Th)-1 and Th2 activation pathway, role of NFAT in immune response, dendritic cell maturation, phagosome formation, and of current global interest, viral response, role of pattern recognition receptors in response to bacteria and viruses, and coronavirus pathogenesis (associated with the upregulation of 2′-5′-oligoadenylate synthetase genes *OAS2* and *OAS3*) (Figure 2c).

### Supplemental LC n-3 PUFAs increase scWAT LC n-3 PUFAs and modulate scWAT oxylipin profile in normal weight individuals

**Figure 3:**
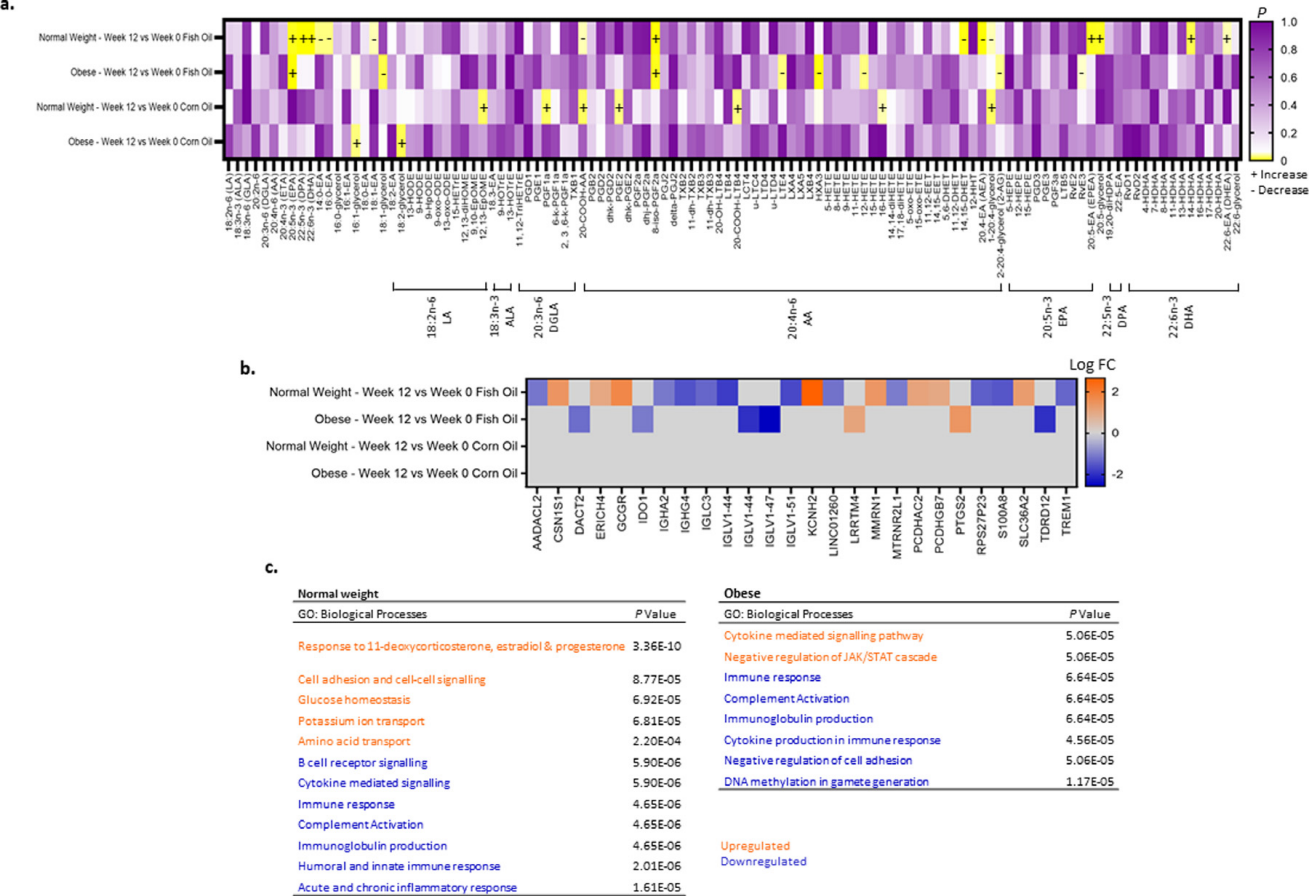
a. Fatty acids and fatty acid-derived mediators in scWAT in response to dietary oil intervention (week-12 vs week-0, Wilcoxon signed ranks test). Significance is defined as *P* < 0.05 (depicted in yellow); +, increase with intervention, -, decrease with intervention. Normal weight individuals (n=39), individuals living with obesity (n=45) b. Log fold change of scWAT genes in response to dietary oil intervention (week-12 - week 0). All fold change data are statistically significant at *P* < 0.001 (pair-wise comparison in EdgeR). Normal weight individuals (n=20), individuals living with obesity (n=20). c. Enriched GO (gene ontology) biological processes in response to 12-week fish oil intervention in normal weight individuals and in those living with obesity (pair-wise comparison in EdgeR) Normal weight individuals (n=20), individuals living with obesity (n=20).

In response to a 12-week LC n-3 PUFA intervention, the proportion of EPA in scWAT significantly increased in both normal weight and individuals living with obesity (Figure 3a). The proportions of DPA and DHA also significantly increased in normal weight individuals and increased to a similar extent in individuals living with obesity but this was not statistically significant (Figure 3a). There was no effect of sex on response to EPA, DPA or DHA (*P* ≥ 0.487 normal weight individuals, *P* ≥ 0.230 individuals living with obesity, Mann-Whitney U, data not shown). Changes (Δ) in the proportions of LC n-3 PUFAs were negatively correlated with markers of insulin resistance. Δ scWAT DPA and DHA were negatively correlated with both HOMA2-IR (ρ=-0.282, *P* = 0.023, and ρ=-0.375, *P* = 0.002 respectively, Spearman's rank order correlation, data not shown) and adipose-IR (ρ=-0.357, *P* = 0.003 and ρ=-0.423, *P* = ≤0.001 respectively, Spearman's rank order correlation, data not shown), and there was a trend for Δ EPA to be negatively correlated with adipose-IR (ρ=-0.224, *P* = 0.068, Spearman's rank order correlation, data not shown). EPA, DPA and DHA did not change in scWAT in the corn oil group (Figure 3a). Saturated and monounsaturated FAs were not altered with either fish oil or corn oil intervention in either group of individuals (*P* ≥ 0.084 normal weight individuals, *P* ≥ 0.123 individuals living with obesity, fish oil intervention, and *P* ≥ 0.060 normal weight individuals, *P* ≥ 0.123 individuals living with obesity, corn oil intervention, Wilcoxon signed ranks test, data not shown).

Several oxylipins were significantly altered by fish oil intervention, predominantly in normal weight individuals (Figure 3a). The proportion of ethanolamides of myristic, palmitic and palmitoleic acids (14:0-EA, 16:0-EA and 16:1-EA) were significantly decreased in the scWAT of normal weight individuals (Figure 3a). The proportion of the 20:4n-6 (AA) metabolites 20-COOH-AA, 14-15-DHET, and arachidonoyl ethanolamide (AEA), were significantly decreased, and the AA metabolite 8-iso-PGF_2α_, significantly increased in the scWAT of normal weight individuals receiving fish oil (Figure 3a). We previously reported an increase in the EPA and DHA containing endocannabinoids eicosapentaenoyl ethanolamide and docosahexaenoyl ethanolamide with fish oil.^13^ In addition to these, we further report a significant increase in the proportion of the EPA glycerol ester, 20:5-glycerol, and the absolute concentration of the hydroxy-DHA metabolite 14-HDHA, the precursor to maresin 1 (MaR1), in normal weight individuals receiving fish oil (Figure 3a.)

### Modulation of scWAT oxylipins by LC n-3 PUFAs is altered in obesity

Fish oil intervention in individuals living with obesity resulted in a decrease of AA metabolites in scWAT but no generation of LC n-3 PUFA metabolites. There was a decrease in the proportion of the glycerol ester of palmitoleic acid, 16:1-glycerol, the AA metabolites LTE_4_ and HXA_3_, and in the absolute concentration of 12-HETE and 2-AG in addition to a decrease in the proportion of the EPA derived SPM RvE3 in individuals living with obesity who received fish oil. The only metabolite similarly changed in individuals living with obesity and in normal weight individuals was the AA metabolite 8-iso-PGF_2α_, which increased (Figure 3a).

Corn oil increased the proportions of the linoleic acid (LA) metabolite 12,13-EpOME, the dihomo-γ-linolenic acid metabolite, PGF_1α_, and the AA metabolites 20-COOH-AA, PGE_2_, 20-COOH-LTB_4_, 16-HETE and 1-20:4-glycerol in normal weight individuals (Figure 3a). In individuals living with obesity, corn oil intervention increased the proportions of the glycerol ester of palmitoleic acid, 16:1-glycerol, and of LA, 18:2-glycerol. The corn oil supplement contained 0.55 g of LA per capsule; the proportions of LA and AA did not significantly increase in the scWAT but the rise in LA, DGLA, and AA metabolites is not surprising given the composition of the oil. Corn oil proved to be a suitable control oil in individuals living with obesity in that only 2 FA metabolites were altered; its use in normal weight individuals may be more questionable due to the increase in AA derived metabolites. However, corn oil had a smaller effect on the oxylipin profile than fish oil and had minimal effects on the expression of scWAT genes in both groups of individuals (Fig 3.b, transcriptome data can be openly accessed via GEO (https://www.ncbi.nlm.nih.gov/geo/ ID: GSE162653).

### LC n-3 PUFAs modulate inflammatory gene expression in scWAT

In response to 12-week fish oil intervention, 51 genes were differentially expressed in scWAT in normal weight individuals (meeting significance criteria of < 0.05 and FDR < 0.1). Of these, 26 genes had at least a 2-fold change in expression and 19 of these were associated with inflammatory and immune response (Figure 3b). In response to 12-week fish oil intervention, 21 genes were differentially expressed in individuals living with obesity (meeting significance criteria of < 0.05 and FDR < 0.1). Eight of these had at least a 2-fold change in expression and 7 of these were associated with inflammatory and immune response (Figure 3c). None of these genes, nor any gene associated with inflammatory and immune response, was significantly modulated in response to corn oil intervention.

The modulation of these genes was associated with the overall downregulation of acute and chronic inflammatory response, humoral and innate immune response, complement activation, immunoglobulin production, B cell receptor signalling, cytokine mediated signalling, and cytokine production in immune response (Figure 3c). Furthermore, modulation of genes in response to 12-week fish oil in normal weight individuals was associated with upregulation of glucose homeostasis (Figure 3c).

In relation to oxylipin metabolism, the expression of the gene encoding *PTGS2* significantly increased by 2.7-fold in scWAT from individuals living with obesity in response to 12-week fish oil intervention, but this was not accompanied by a change in the activity of COX-2 in these individuals or in normal weight individuals (*P* ≥ 0.166, Paired T-test, data not shown).

## Discussion

Comprehensive evaluation of ‘physiological’ scWAT and changes in obesity in the context of inflammation and in response to LC n-3 PUFAs is lacking. The current study provides evidence for altered n-3 and n-6 FA composition, altered oxylipins derived from these FAs, and an altered transcriptome in scWAT from individuals living with obesity compared to those of healthy weight. These differences are indicative of enhanced inflammation and immune response with dysregulation of resolution (of inflammation) suggesting individuals living with obesity have a reduced capability to self-resolve inflammation which may contribute to a state of chronic inflammation often observed in obesity. Furthermore, sustained inflammatory and immune response observed in individuals living with obesity may contribute to dysregulation in their ability to respond appropriately to inflammatory and immune queues such as pathogen attack with increased susceptibility and severity of infections in these individuals. In addition, the current study reports modulation of scWAT oxylipin and transcriptome profiles to promote anti-inflammatory signalling and inhibit chronic inflammation and immune response with LC n-3 PUFA intervention, but that these actions of LC n-3 PUFAs are impaired in individuals living with obesity compared to normal weight individuals.

**Figure 4:**
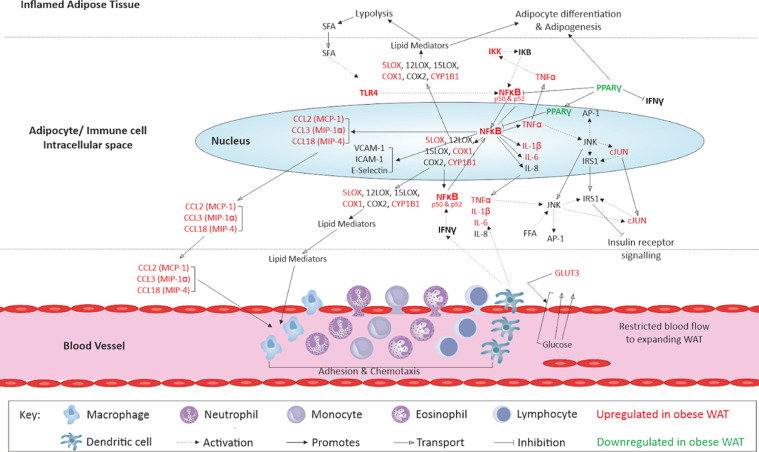
An overview of scWAT inflammatory signalling by genes dysregulated in individuals living with obesity at study entry (week-0) and their upstream regulators.

Transcriptional changes in obesity were associated with the upregulation of inflammatory and immune responses, and changes to lipid and carbohydrate metabolism which may indicate interruption of whole tissue homeostasis occurring in these early stages of obesity. Several genes involved in key tissue processes including interleukin signalling, inflammatory transcription factor activation, chemokine signalling, and function of enzymes involved in oxylipin synthesis were differentially expressed. An overview of such genes and their actions in the scWAT of individuals living with obesity is depicted in Figure 4. ‘Immune signalling and response’ was dysregulated in individuals living with obesity in which there was differential expression of genes involved in B- and T-cell signalling (Figure 2b).

### Dysregulation of immune responses in obesity-link to SARS-CoV-2 susceptibility

A novel finding of the current study is the upregulation of *OAS2* and *OAS3,* genes that encode oligoadenylate synthetases that synthesize 2′-5′-linked oligoadenylates, in individuals living with obesity. These proteins are involved in the coronavirus pathogenesis pathway (Figure 3c) and have a role in activating RNase L leading to the degradation of viral ssRNA.^44^ OAS variants have been implicated in susceptibility to SARS-CoV-2.^45^ Analysis of biobank data has shown obesity to almost double the risk of infection when adjusted for age, sex, ethnicity, and socioeconomic status,^46^ and in the UK, of 13,503 individuals hospitalised with SARS-CoV-2, 70.2% of these were living with overweight and obesity, and 36.2% of these with obesity.^5^ Adipose tissue has been proposed to be a target for SARS-CoV-2 due to high expression of angiotensin-converting enzyme 2 (ACE2), a SARS-CoV-2 receptor and this may link obesity and increased risk of susceptibility to the disease.^7^^,^^47^ However, current literature does not report any changes to *ACE2* expression in obesity^48^ and data from the current study support this (Supplemental Table 4).

There has been some discussion that obesity promotes deficiency of SPMs^25^ and the current study provides important data to support this. Because of the role of SPMs in supporting immunity, low levels of SPMs in obesity may contribute to increased susceptibility to infection.^25^ There is strong literature supporting the role for SPMs in improving outcomes to bacterial and viral infection^49^^,^^50^ in mice^51^^,^^52^ and in humans.^53^ Despite deficiency of these SPMs, B cell signalling was upregulated in individuals living with obesity in the current study. This, in addition to greater expression of OAS genes, suggests dysregulation of immune response in individuals living with obesity. Chronic upregulation of the immune system and interference from chronic inflammatory signalling in these individuals may result in failure to respond appropriately to challenges such as bacterial and viral infections.

### Dysregulation of the oxylipin profile in obesity

Deficiency of SPMs and the intermediates in their biosynthesis in the scWAT of individuals living with obesity is a novel finding. We report decreased proportions of SPMs, RvE1, RvD2 and LXB_4_, and a range of hydroxy-DHA metabolites which are precursors to Rvs, protectin D (PD) and MaRs.^19^ Hydroxy-DHA metabolites have anti-angiogenic actions, inhibit platelet aggregation and neutrophil infiltration, and promote phagocytosis of dying cells by macrophages.^54^, ^55^, ^56^ 17-HDHA is of particular interest as the precursor to RvD1 and has been reported to be decreased in WAT in obese mice and in plasma in human obesity.^57^, ^58^, ^59^ 14-HDHA, the precursor to MaR1, has been previously observed at lower concentrations in the plasma of obese humans in comparison to lean,^59^ in individuals living with morbid obesity accompanied by type-diabetes in comparison to non-diabetic individuals living with mild obesity^60^ and at lower concentrations in the adipose tissue of C57BL/6 mice with diet-induced obesity in comparison to adipose tissue from lean mice.^23^ Altered concentrations of SPMs in human obesity in comparison to normal weight individuals have previously only been reported in visceral WAT (vWAT) in which there was an increase in several SPMs in obesity suggesting association between obesity and adipose SPMs is depot specific.^61^ The current study is the first to report decreased proportions of SPMs and HDHAs in scWAT in human obesity without accompanied metabolic complications in comparison to normal weight individuals (previous studies reviewed by Pal et al. and Han at al.^20^^,^^25^^,^^26^^,^^61^).

### Genetic regulation of the oxylipin profile in obesity

There was a lack of association between the altered oxylipin profile and the expression of genes encoding enzymes involved in the oxidation of FAs to form oxylipins in individuals living with obesity. The mRNA expression of *12-LOX* was negatively correlated with body fat % which is concordant with lower proportions of 14-HDHA in individuals living with obesity; however, greater expression of *5-LOX, COX-1* and *CYP1B1* was observed in individuals living with obesity which was not concordant with the altered proportion of respective pathway oxylipins in these individuals. Furthermore, we report negative correlations between several DHA-derived Rvs and HDHAs with the expression of 15-LOX which is not involved in the biosynthetic pathways of these oxylipins. We suggest this may reflect preferential metabolism of DHA by 15-LOX resulting in lower proportions of DHA-derived oxylipins produced via 5-LOX, 12-LOX, and COX enzymes.

What may have a greater influence on the oxylipin profile is the expression of the very long-chain acyl-CoA synthetase gene, *SLC27A2* which is responsible for converting and ‘activating’ DHA to its acyl-Co-A ester required for further metabolism to oxylipins.^62^ We previously reported 92% lower expression of *SLC27A2* in scWAT of individuals living with obesity at study entry.^13^ We reported that subsequent lower activation of DHA by SLC27A2, may result in less conversion of DHA to the DHA containing ethanolamide, providing a mechanism for the impaired synthesis of DHA-ethanolamide following LC n-3 PUFA intervention in obesity.^13^ In the current analysis, this lower expression of *SLC27A2* may contribute to lower proportions of Rvs and HDHAs observed at study entry and may explain the lack of formation of 14-HDHA in response to LC n-3 PUFA intervention in individuals living with obesity (Figure 3a). The expression of *SLC27A2* was also negatively correlated with HOMA2-IR (ρ -0.394, *P* = 0.001, Spearman's rank order correlation, data not shown), suggesting the metabolic health of the individual may play a role in its expression and subsequent LC n-3 PUFA activation for the formation of DHA-derived oxylipins.

### Altered response to LC n-3 PUFA intervention in obesity

The proportion of EPA and DHA increased to a similar extent in individuals living with obesity as in normal weight individuals, despite the response of DHA not being statistically significant in those living with obesity (Figure 3a; change in FA shown in more detail in^13^). It is important to note that individuals living with obesity had ∼3 times the amount of body fat as normal weight individuals; for the proportion of EPA and DHA to have increased to a similar extent, the absolute amount of EPA and DHA in the tissue in individuals living with obesity may be ∼3 times greater than that of normal weight individuals. This finding magnifies the apparent dysregulation in LC n-3 PUFA metabolism in individuals living with obesity as we observe a lack of response in oxylipin formation from these FAs in comparison to normal weight individuals despite the likely higher abundance of the precursor FAs (Figure 3a). Additional investigation of blood and plasma NEFAs revealed that there were lower concentrations of non-esterified LC n-3 PUFAs following fish oil intervention in individuals living with obesity than in normal weight individuals (Supplemental Table 5). There were no differences in these or the concentration of total NEFAs at study entry which may suggest there were no differences in the rate of lipolysis or specifically the release of EPA and DHA during lipolysis in obesity. Lower concentrations of non-esterified EPA and DHA in conjunction with data reporting similar proportions of scWAT EPA and DHA following fish oil intervention in individuals living with obesity compared to normal weight individuals (Supplemental Table 5) may suggest retention of these FAs within the tissue. This may reflect differential storage of these lipids, changes to their liberation from fat stores and cell membranes, or processes required for metabolism to further bioactive molecules.

Metabolism of FAs residing in the membranes of scWAT cells is required for the generation of bioactive FA metabolites. If EPA and DHA are not incorporated into the cell membrane or their contribution in the membrane is not increased in comparison to other FAs, they will not be available or preferred for metabolism and subsequent metabolites will not be generated. Alternatively, it may be that DHA is retained in the membrane or not converted into is acyl-CoA ester which would impair further metabolism. We report lower expression of *SLC27A2* which may contribute to reduced acyl-Co-A ester formation, and subsequently impaired generation of 14-HDHA in response to LC n-3 PUFA intervention (Figs. 2a and 3a) in individuals living with obesity. Therefore, altered storage, activation, and metabolism of LC n-3 PUFAs in individuals living with obesity may contribute to greater concentrations retained within the scWAT and impaired generation of EPA and DHA metabolites, compared to what is seen in normal weight individuals.

We further report downregulated expression of scWAT genes involved in immune and inflammatory signalling in response to LC n-3 PUFA intervention but that these effects were impaired in individuals living with obesity. Furthermore, the current study does not provide evidence in support of LC n-3 PUFA modulated expression of key inflammatory markers such as IL-1β, IL-6, tumour necrosis factor- alpha (TNF-α), and NF-kB as previously reported in human derived lipopolysaccharide (LPS) stimulated macrophages,^63^ and cultured DCs and stimulated macrophages.^64^, ^65^, ^66^ In addition to immune and inflammatory responses, LC n-3 PUFA intervention significantly upregulated glucagon receptor2 (*GCGR*) and transglutaminase 2 (*TGM2*) which are associated with decreased adipogenesis and may result in improved glucose tolerance and insulin sensitivity,^67^ as well as accelerated clearance of apoptotic cells in areas of inflammation.^68^ These are key steps in the resolution of scWAT inflammation and dysfunction, again suggesting a benefit of LC n-3 PUFAs in scWAT, especially in normal weight individuals.

**Figure 5:**
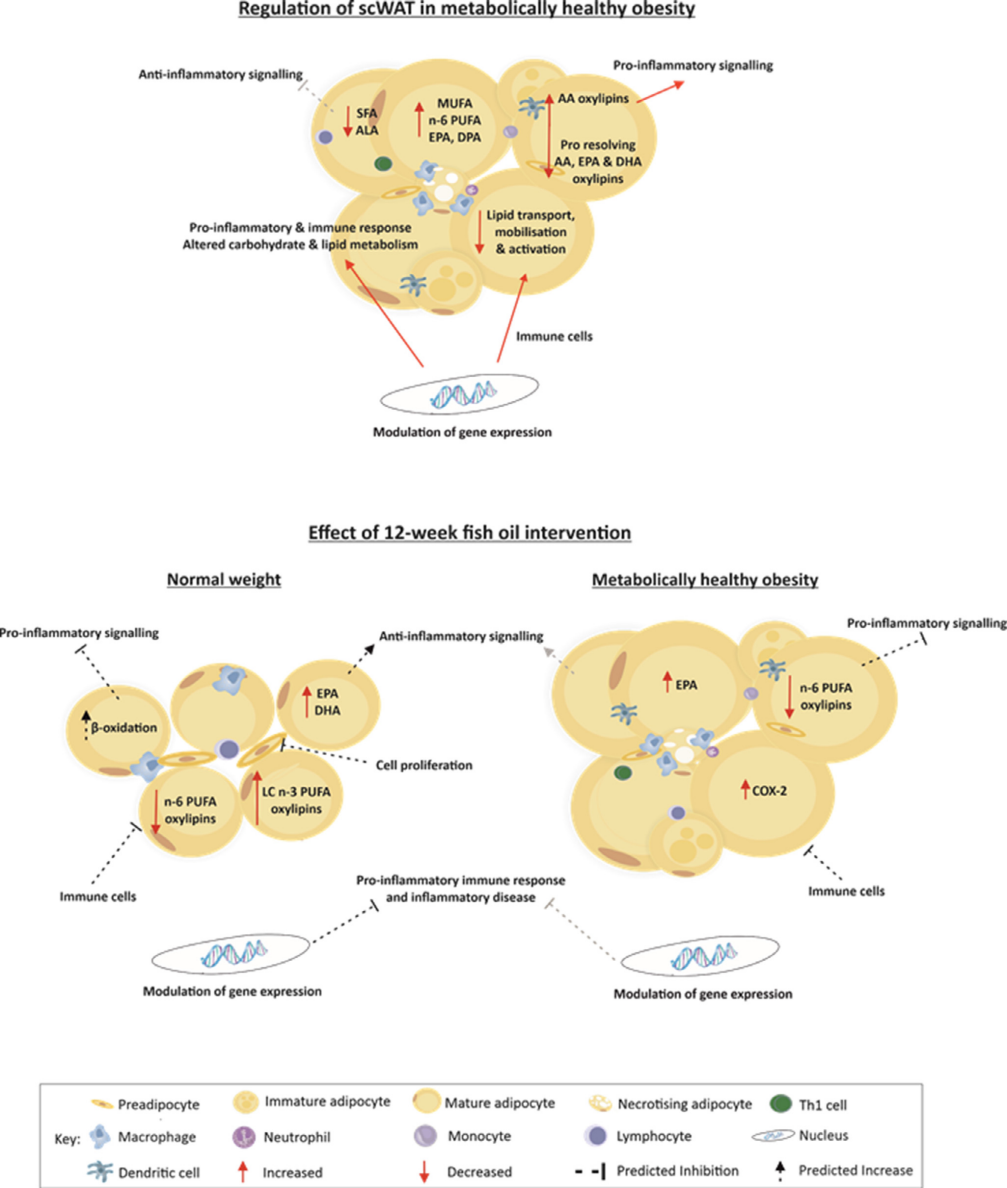
Summary of scWAT dysregulation in individuals living with obesity, and the effects of LC n-3 PUFA intervention in normal weight individuals and those living with obesity.

LC n-3 PUFA intervention did not alter the proportion of AA in scWAT, resulting in individuals living with obesity still having a higher proportion of AA in comparison to normal weight individuals following the intervention. For LC n-3 PUFAs to elicit their actions, they are incorporated into cell membranes where they alter lipid raft formation and membrane fluidity, subsequently altering the activity of transcription factors, and the synthesis and secretion of inflammatory signalling molecules.^28^^,^^29^ It may be that despite incorporation of LC n-3 PUFAs resulting in similar proportions of EPA and DHA in the scWAT, this is not enough to alter the n-6: n-3 ratio in individuals living with obesity to promote favourable metabolism of these FAs and subsequently we do not see the effects on LC n-3 PUFA derived oxylipins or as great an effect on gene expression in these individuals in comparison to normal weight individuals. The decrease in the proportion of AA metabolites in individuals living with obesity, suggests changes to the metabolism of AA in response to fish oil intervention. We previously reported no changes to the expression of genes or enzymes involved in AA metabolism to oxylipins, so this may suggest displacement of AA by EPA and DHA incorporation to scWAT cell membranes. However, this incorporation may not be enough to promote LC n-3 PUFA derived metabolites as we observe a lack of increased generation of these in obesity. As discussed above, this may also suggest impaired incorporation of DHA to cell membranes or retention of DHA in the membrane, which may in part be due to impaired activation to its acyl-CoA ester required for metabolism to SPMs. An overview of scWAT dysregulation and the effects of 12-week LC n-3 PUFA intervention is depicted in Figure 5.

In summary, the current study provides novel evidence for dysregulation of oxylipin and transcriptome profiles in scWAT in obesity prior to manifestation of metabolic syndrome as determined by circulating markers. These new data suggest dysregulation of immune response, enhanced inflammation, and declining ability to self-resolve inflammation in scWAT in individuals living with obesity which may contribute to the progression of obesity associated metabolic complication. We report both LC n-3 PUFA status and response, as well as the oxylipin profile, to be associated with HOMA2-IR indicating a link between n-3 PUFAs, their oxylipins and metabolic health. We provide novel evidence that scWAT is responsive to dietary manipulation with LC n-3 PUFAs and that beneficial changes to inflammatory state through the modulation of tissue oxylipin and transcriptome profiles can be achieved within 12 weeks of LC n-3 PUFA intervention in normal weight individuals. Furthermore, the current study provides novel evidence that the beneficial actions of LC n-3 PUFAs are impaired in individuals living with obesity; this may, at least in part, be due to altered FA incorporation to cell membranes, activation, and metabolism as a result of decreased expression of *SLC27A2*in individuals living with obesity. We further highlight the role of adipose tissue and the potential impact of obesity in viral responses and that *OAS* genes and *SLC27A2* may be novel targets for future therapeutic intervention. Furthermore, these data may suggest personalisation of EPA+DHA intake is required for individuals living with varying stages of obesity and with declining metabolic health.

### Strength and limitations

The current study has several strengths. These include its sample size, the compliance to the intervention which was >90%, the careful phenotyping of the individuals, and the use of an intervention to potentially modify scWAT oxylipin and transcriptome profiles which generated highly novel data. We have shown that 12 weeks of 1.9 g of EPA + DHA daily was adequate to increase EPA and DHA in human scWAT and to alter oxylipin and transcriptome profiles in both normal weight individuals and individuals living with obesity. This dose of EPA + DHA is attainable amongst the general population and could be achieved by diet (e.g. several servings of fatty fish weekly) or a combination of diet and supplementation.

A limitation of this study is that absolute quantification of FAs was not determined so it is not possible to determine whether individuals living with obesity indeed have ∼3 times greater EPA and DHA retained in their scWAT following the intervention or not. In addition, full dietary data, including dietary fat intake, which may have varied between the two groups, was not collected, and could not be assessed as a possible contributor to differences in FA composition and oxylipin profile at study entry. A target group size of 40 normal weight individuals and 40 individuals living with obesity was required for the study to be appropriately powered to detect changes in circulating cytokines (using IL-6 as the primary outcome) at > 80% power and a 5% level of significance. There was no formal power calculation for the outcomes described in the current paper, and it is possible that the number of scWAT samples analysed was a limitation for some of the outcomes reported herein.

### Data sharing statement

All RNA-seq raw data generated for the present study are publicly available in GEO under accession code GSE162653.

## Contributors

Helena L. Fisk: Data curation, Formal analysis, Investigation, Methodology, Writing - original draft, Writing - review and editing. Caroline E. Childs: Formal analysis, Project administration, Writing - review and editing. Elizabeth A. Miles: Conceptualisation, Writing - review and editing. Robert Ayres: Formal analysis, Writing - review and editing. Paul S. Noakes: Formal analysis, Project administration, Writing - review and editing. Carolina Paras-Chavez: Investigation, Project administration, Writing - review and editing. Ondrej Kuda: Formal analysis, Writing - review and editing. Jan Kopecký: Formal analysis, Writing - review and editing. Elie Antoun: Data curation, Writing - review and editing. Karen A. Lillycrop: Data curation, Writing - review and editing. Philip C. Calder: Conceptualisation, Funding acquisition, Supervision, Writing - review and editing. All authors read and approved the final version of the manuscript.

## Declaration of interests

P.C. Calder undertakes unpaid voluntary work as the current President of the Federation of European Nutrition Societies (FENS) and as Past President of ILSI Europe. P.C Calder received funding from the European Commission Seventh Framework Programme (Grant Number 244995). O. Kuda received funding from the Czech Academy of Sciences (Lumina quaeruntur LQ200111901). K.A. Lillycrop holds a contract with Benevolent AI Ltd. E.A. Miles received payment from Abbott Nutrition to present at The British Society for Allergy & Clinical Immunology (BSACI) conference in 2019. There are no other declarations of interests.

## Appendix Supplementary materials

Image, application 1

Image, application 2

Image, application 3

Image, application 4

Image, application 5

Image, application 6

Image, application 7

Image, application 8

Image, application 9

## References

[1] WHO (2018). http://www.who.int/news-room/fact-sheets/detail/obesity-and-overweight.

[2] British Heart Foundation (2018).

[3] National Statistics, NHS Digital (2017).

[4] Public Health England (2014).

[5] Gao M., Piernas C., Astbury N.M. (2021). Associations between body-mass index and COVID-19 severity in 6·9 million people in England: a prospective, community-based, cohort study. Lancet Diabetes Endocrinol.

[6] Stefan N., Birkenfeld A.L., Schulze M.B. (2021). Global pandemics interconnected - obesity, impaired metabolic health and COVID-19. Nat Rev Endocrinol.

[7] Mohammad S., Aziz R., Al Mahri S. (2021). Obesity and COVID-19: what makes obese host so vulnerable?. Immun Ageing.

[8] Daryabor G., Kabelitz D., Kalantar K. (2019). An update on immune dysregulation in obesity-related insulin resistance. Scand J Immunol.

[9] Pérez de Heredia F., Gomez-Martinez S., Marcos A. (2012). Obesity, inflammation and the immune system. Proc Nutr Soc.

[10] Masoodi M., Kuda O., Rossmeisl M., Flachs P., Kopecky J. (2014). Lipid signaling in adipose tissue: connecting inflammation & metabolism. Biochim Biophys Acta.

[11] Calder P.C. (2011). Fatty acids and inflammation: the cutting edge between food and pharma. Eur J Pharmacol.

[12] Calder P.C., Ahluwalia N., Brouns F. (2011). Dietary factors and low-grade inflammation in relation to overweight and obesity. Br J Nutr.

[13] Fisk H.L., Childs C.E., Miles E.A. (2021). Dysregulation of endocannabinoid concentrations in human subcutaneous adipose tissue in obesity and modulation by omega-3 polyunsaturated fatty acids. Clin Sci (Lond).

[14] Bittleman D.B., Casale T.B. (1995). 5-hydroxyeicosatetraenoic acid (HETE)-induced neutrophil transcellular migration is dependent upon enantiomeric structure. Am J Respir Cell Mol Biol.

[15] Wright H.L., Moots R.J., Bucknall R.C., Edwards S.W. (2010). Neutrophil function in inflammation and inflammatory diseases. Rheumatol (Oxf).

[16] Hecker G., Ney P., Schrör K. (1990). Cytotoxic enzyme release and oxygen centered radical formation in human neutrophils are selectively inhibited by E-type prostaglandins but not by PGI2. Naunyn Schmiedebergs Arch Pharmacol.

[17] Fain J.N., Leffler C.W., Cowan G.S., Buffington C., Pouncey L., Bahouth S.W. (2001). Stimulation of leptin release by arachidonic acid and prostaglandin E(2) in adipose tissue from obese humans. Metabolism.

[18] Balvers M.G., Verhoeckx K.C., Plastina P., Wortelboer H.M., Meijerink J., Witkamp R.F. (2010). Docosahexaenoic acid and eicosapentaenoic acid are converted by 3T3-L1 adipocytes to N-acyl ethanolamines with anti-inflammatory properties. Biochim Biophys Acta.

[19] Serhan C.N., Chiang N., Dalli J., Levy B.D. (2015). Lipid mediators in the resolution of inflammation. Cold Spring Harb Perspect Biol.

[20] Han Y.H., Lee K., Saha A. (2021). Specialized proresolving mediators for therapeutic interventions targeting metabolic and inflammatory disorders. Biomol Ther (Seoul).

[21] Schwab J.M., Chiang N., Arita M., Serhan C.N. (2007). Resolvin E1 and protectin D1 activate inflammation-resolution programmes. Nature.

[22] Croasdell A., Sime P.J., Phipps R.P. (2016). Resolvin D2 decreases TLR4 expression to mediate resolution in human monocytes. FASEB J.

[23] Claria J., Dalli J., Yacoubian S., Gao F., Serhan C.N. (2012). Resolvin D1 and resolvin D2 govern local inflammatory tone in obese fat. J Immunol.

[24] Serhan C.N. (2014). Pro-resolving lipid mediators are leads for resolution physiology. Nature.

[25] Pal A., Gowdy K.M., Oestreich K.J., Beck M., Shaikh S.R. (2020). Obesity-driven deficiencies of specialized pro-resolving mediators may drive adverse outcomes during SARS-CoV-2 infection. Front Immunol.

[26] Claria J., Nguyen B.T., Madenci A.L., Ozaki C.K., Serhan C.N. (2013). Diversity of lipid mediators in human adipose tissue depots. Am J Physiol Cell Physiol.

[27] Seppanen-Laakso T., Laakso I., Lehtimaki T. (2010). Elevated plasma fibrinogen caused by inadequate alpha-linolenic acid intake can be reduced by replacing fat with canola-type rapeseed oil. Prostaglandins Leukot Essent Fatty Acids.

[28] Calder P.C. (2014). Very long chain omega-3 (n-3) fatty acids and human health. Eur J Lipid Sci Technol.

[29] Calder P.C. (2015). Marine omega-3 fatty acids and inflammatory processes: effects, mechanisms and clinical relevance. Biochim Biophys Acta.

[30] Nisoli E., Carruba M.O., Tonello C., Macor C., Federspil G., Vettor R. (2000). Induction of fatty acid translocase /CD36, peroxisome proliferator-activated receptor- 2, leptin, uncoupling proteins 2 and 3, and tumor necrosis factor- gene expression in human subcutaneous fat by lipid infusion. Diabetes.

[31] Mejia-Barradas C.M., Del-Rio-Navarro B.E., Dominguez-Lopez A. (2014). The consumption of n-3 polyunsaturated fatty acids differentially modulates gene expression of peroxisome proliferator-activated receptor alpha and gamma and hypoxia-inducible factor 1 alpha in subcutaneous adipose tissue of obese adolescents. Endocrine.

[32] Larsen T.M., Toubro S., Astrup A. (2003). PPARgamma agonists in the treatment of type II diabetes: is increased fatness commensurate with long-term efficacy?. Int J Obes Relat Metab Disord.

[33] Villaret A., Galitzky J., Decaunes P. (2010). Adipose tissue endothelial cells from obese human subjects: differences among depots in angiogenic, metabolic, and inflammatory gene expression and cellular senescence. Diabetes.

[34] Dewhurst-Trigg R., Hulston C.J., Markey O. (2020). The effect of quantity and quality of dietary fat intake on subcutaneous white adipose tissue inflammatory responses. Proc Nutr Soc.

[35] Rossmeisl M., Pavlisova J., Janovska P. (2018). Differential modulation of white adipose tissue endocannabinoid levels by n-3 fatty acids in obese mice and type 2 diabetic patients. BBA Mol Cell Biol Lipids.

[36] Polus A., Zapala B., Razny U. (2016). Omega-3 fatty acid supplementation influences the whole blood transcriptome in women with obesity, associated with pro-resolving lipid mediator production. BBA Mol Cell Biol Lipids.

[37] Martinez-Fernandez L., Laiglesia L.M., Huerta A.E., Martinez J.A., Moreno-Aliaga M.J. (2015). Omega-3 fatty acids and adipose tissue function in obesity and metabolic syndrome. Prostaglandins Other Lipid Mediat.

[38] Baker C. (2021).

[39] Fisk H.L., West A.L., Childs C.E., Burdge G.C., Calder P.C. (2014). The use of gas chromatography to analyze compositional changes of fatty acids in rat liver tissue during pregnancy. J Vis Exp.

[40] Kuda O., Brezinova M., Rombaldova M. (2016). Docosahexaenoic acid–derived fatty acid esters of hydroxy fatty acids (FAHFAs) with anti-inflammatory properties. Diabetes.

[41] Kim D., Pertea G., Trapnell C., Pimentel H., Kelley R., Salzberg S. (2013). TopHat2: accurate alignment of transcriptomes in the presence of insertions, deletions and gene fusions. Genome Biol.

[42] Anders S., Pyl P., Huber W. (2014). HTSeq-a python framework to work with high-throughput sequencing data. Bioinformatics.

[43] McCarthy D., Chen Y., Smyth G. (2012). Differential expression analysis of multifactor RNA-Seq experiments with respect to biological variation. Nucleic Acids Res.

[44] Li Y., Banerjee S., Wang Y. (2016). Activation of RNase L is dependent on OAS3 expression during infection with diverse human viruses. Proc Natl Acad Sci USA.

[45] Pairo-Castineira E., Clohisey S., Klaric L. (2021). Genetic mechanisms of critical illness in COVID-19. Nature.

[46] Ho F.K., Celis-Morales C.A., Gray S.R. (2020). Modifiable and non-modifiable risk factors for COVID-19: results from UK Biobank. BMJ Open.

[47] Kassir R. (2020). Risk of COVID-19 for patients with obesity. Obes Rev.

[48] Pinheiro T.A., Barcala-Jorge A.S., Andrade J.M.O. (2017). Obesity and malnutrition similarly alter the renin-angiotensin system and inflammation in mice and human adipose. J Nutr Biochem.

[49] Tam Vincent C., Quehenberger O., Oshansky Christine M., Suen R., Armando Aaron M., Mea T.P. (2013). Lipidomic profiling of influenza infection identifies mediators that induce and resolve inflammation. Cell.

[50] Basil M.C., Levy B.D. (2016). Specialized pro-resolving mediators: endogenous regulators of infection and inflammation. Nat Rev Immunol.

[51] Teague H., Fhaner C., Harris M., Duriancik D., Reid G., Shaikh S. (2013). n-3 PUFAs enhance the frequency of murine B-cell subsets and restore the impairment of antibody production to a T-independent antigen in obesity. J Lipid Res.

[52] Ramon S., Baker S., Sahler J. (2014). The specialized proresolving mediator 17-HDHA enhances the antibody-mediated immune response against influenza virus: a new class of adjuvant?. J Immunol.

[53] Kim N., Lannan K., Thatcher T., Pollock S., Woeller C., Phipps R. (2018). Lipoxin B4 enhances human memory b cell antibody production via upregulating cyclooxygenase-2 expression. J Immunol.

[54] Sapieha P., Stahl A., Chen J. (2011). 5-lipoxygenase metabolite 4-HDHA is a mediator of the antiangiogenic effect of ω-3 polyunsaturated fatty acids. Sci Transl Med.

[55] Yeung J., Hawley M., Holinstat M. (2017). The expansive role of oxylipins on platelet biology. J Mol Med (Berl).

[56] Serhan C.N., Yang R., Martinod K. (2009). Maresins: novel macrophage mediators with potent antiinflammatory and proresolving actions. J Exp Med.

[57] Neuhofer A., Zeyda M., Mascher D. (2013). Impaired local production of proresolving lipid mediators in obesity and 17-hdHA as a potential treatment for obesity-associated inflammation. Diabetes Obes Metab.

[58] Crouch M.J., Kosaraju R., Guesdon W. (2019). Frontline science: a reduction in DHA-derived mediators in male obesity contributes toward defects in select B cell subsets and circulating antibody. J Leukoc Biol.

[59] Lopez-Vicario C., Titos E., Walker M.E. (2019). Leukocytes from obese individuals exhibit an impaired SPM signature. FASEB J.

[60] Schulte F., Asbeutah A.A., Benotti P.N. (2020). The relationship between specialized pro-resolving lipid mediators, morbid obesity and weight loss after bariatric surgery. Sci Rep.

[61] Titos E., Rius B., Lopez-Vicario C. (2016). Signaling and immunoresolving actions of resolvin D1 in inflamed human visceral adipose tissue. J Immunol.

[62] Watkins P.A. (2008). Very-long-chain acyl-CoA synthetases. J Biol Chem.

[63] Weldon S.M., Mullen A.C., Loscher C.E., Hurley L.A., Roche H.M. (2007). Docosahexaenoic acid induces an anti-inflammatory profile in lipopolysaccharide-stimulated human THP-1 macrophages more effectively than eicosapentaenoic acid. J Nutr Biochem.

[64] Lee J.Y., Sohn K.H., Rhee S.H., Hwang D. (2001). Saturated fatty acids, but not unsaturated fatty acids, induce the expression of cyclooxygenase-2 mediated through Toll-like receptor 4. J Biol Chem.

[65] Novak T.E., Babcock T.A., Jho D.H., Helton S.W., Espat J.N. (2003). NF-KB inhibition by n-3 fatty acids modulates LPS-stimulated macrophage TNF-α transcription. Am J Physiol Lung Cell Mol Physiol.

[66] Kong W., Yen J.H., Vassiliou E., Adhikary S., Toscano M.G., Ganea D. (2010). Docosahexaenoic acid prevents dendritic cell maturation and *in vitro* and *in vivo* expression of the IL-12 cytokine family. Lipids Health Dis.

[67] Porzio O., Massa O., Cunsolo V. (2007). Missense mutations in the TGM2 gene encoding transglutaminase 2 are found in patients with early-onset type 2 diabetes. Mutation in brief no. 982. Online. Hum Mutat.

[68] Murtaugh M.P. (1984). Induction of tissue transglutaminase in human peripheral blood monocytes. J Exp Med.

