# Supplementary material for: Modification of subcutaneous white adipose tissue inflammation by omega-3 fatty acids is limited in human obesity-a double blind, randomised clinical trial
Source: eBioMedicine. 2022 Mar 2;77:103909. doi: 10.1016/j.ebiom.2022.103909 (PMC8894262; doi:10.1016/j.ebiom.2022.103909)
Supplement: Supplementary file 1 [file mmc1.docx]

**Supplemental Figure 1**. CONSORT diagram of participant inclusion and ﬂow through the study.

Reproduced from Fisk *et al.* (13).

**Supplemental Figure 2**. Principal component analysis of samples selected for RNA-Seq analysis.

**Supplementary Table 1**. Composition of intervention oils.

**Supplementary Table 2**. Primer sequences.

**Supplemental Table 3**. Full list of genes differentially expressed in scWAT of individuals living with obesity in comparison to normal weight at study entry (meeting Log2 FC > 2, *P* < 0.05, and FDR <0.05).

**Supplementary Table 4**. Expression of *ACE2* in individuals living with obesity in comparison to normal weight individuals at study entry (week-0).

**Supplementary Table 5**. RBC, NEFA and scWAT EPA and DHA at week-0 and week-12.

Significance defined as <0.050. *P* obtained by Mann-Whitney U test comparing data from normal weight individuals and individuals living with obesity at study entry (week-0) and following fish oil intervention (week-12).
