# Supplementary material for: Modification of subcutaneous white adipose tissue inflammation by omega-3 fatty acids is limited in human obesity-a double blind, randomised clinical trial
Source: eBioMedicine. 2022 Mar 2;77:103909. doi: 10.1016/j.ebiom.2022.103909 (PMC8894262; doi:10.1016/j.ebiom.2022.103909)
Supplement: Supplementary file 2 [file mmc2.docx]

**Study Protocol (Version 4a; dated 21/9/2012)**

**1. Title of project**

Omega-3 fatty acids and inflammation in normal weight and obese subjects

Short title: Omega-3 fatty acids and inflammation

**2. Chief investigator**

Professor P.C. Calder, Institute of Human Nutrition, Faculty of Medicine, University of Southampton

**3. Funder of project**

European Commission

**4. Duration of research**

36 months from start date

**5. Places where research will be conducted**

University of Southampton and Wellcome Trust Clinical Research Facility, Southampton University Hospitals NHS Trust

**6. Researchers involved**

Professor P.C. Calder, Professor C.D. Byrne, Dr J. Hopkins, Dr P. Noakes, Miss C. Paras Chavez

**7. Purpose of project/Background**

Seafood, especially oily fish, and fish oil capsules contain relatively high amounts of two long chain omega-3 (n-3) fatty acids called eicosapentaenoic acid (EPA; 20:5n-3) and docosahexaenoic acid (DHA; 22:6n-3). These fatty acids are known to be important for human health [1,2] and the UK Government recommends that their intake be increased [3]. Amongst the physiological effects of n-3 fatty acids is an anti-inflammatory action [4]. Obesity is now recognised as a state associated with chronic low-grade inflammation [5]. In response to a meal there is a transient increase in inflammation, manifested as increased concentrations of inflammatory mediators in the bloodstream [6]; this is referred to a post-prandial inflammation. This response is exaggerated by a high fat content of the meal and in obese subjects and type 2 diabetics [7,8]. Inflammation is considered to increase cardiovascular risk [9]. Thus, the increased inflammatory state in obesity and following consumption of a meal are believed to increase cardiovascular risk [10]. Most demonstrations of the anti-inflammatory effect of n-3 fatty acids have been in healthy individuals or in the context of cardiovascular disease or a frank inflammatory condition like rheumatoid arthritis. It is not clear whether n-3 fatty acids exert anti-inflammatory effects in obese subjects or in the post-prandial state. This research will investigate these two areas. This research is important to regulators, health professionals and the health service, consumers and specific patient groups (e.g. those with obesity). We plan to investigate the anti-inflammatory action of n-3 fatty acids in normal weight and obese subjects.

**8. Objectives**

The overall objective is to identify whether n-3 fatty acids are anti-inflammatory in both normal weight and obese subjects.

The specific objectives are:

1. To follow the appearance of inflammatory mediators in the bloodstream of normal weight and obese subjects over 6 hours following consumption of a standard high fat meal and of the same meal consumed in conjunction with capsules providing EPA+DHA.
2. To identify the concentrations of inflammatory mediators in the bloodstream of normal weight and obese subjects before and after 12 weeks of consumption of capsules providing EPA+DHA or placebo.
3. To identify the presence of inflammatory cells and expression of inflammatory genes and proteins in adipose tissue of normal weight and obese subjects before and after 12 weeks of consumption of capsules providing EPA+DHA or placebo.
4. To follow the appearance of inflammatory mediators in the bloodstream of normal weight and obese subjects who have been consuming n-3 fatty acids or placebo for 12 weeks over 6 hours following consumption of a standard high fat meal.

The questions being addressed by this study are:

1. Is the post-prandial inflammatory response different in obesity?
2. Can consuming marine n-3 fatty acids with a meal alter the post-prandial inflammatory response to that meal?
3. Is the chronic effect of marine n-3 fatty acids on inflammation different between normal weight and obese subjects?
4. Can marine n-3 fatty acids reduce inflammation within adipose tissue?
5. Does chronic use of marine n-3 fatty acids alter the post-prandial inflammatory response?

**9. The study**

*General approach to be taken*

We will recruit normal weight and obese subjects. For the purpose of this study obesity will be defined as a body mass index (BMI) > 30 kg/m^2^ and a waist circumference > 94 cm for men and > 80 cm for women. Subjects’ post-prandial inflammatory response to a high fat meal and to n-3 fatty acids taken with that meal will be assessed using approaches we have used previously to study acute lipid metabolism and fatty acid appearance in the bloodstream [8,9]. Blood will be taken at several time points during the meal challenge (0, 1, 2, 3, 4 and 6 hours). Each subject will undergo both meal challenges, in random order and separated by at least 2 weeks. Subjects will then be randomised to consume placebo or “fish oil” capsules daily for about 12 weeks. Inflammatory mediators in the bloodstream will be measured at the start and end of this period. Subjects will then repeat the standard meal challenge. A range of inflammatory mediators and related hormones will be measured in the blood samples. In addition, fatty acid status will be measured in all samples. An adipose tissue biopsy will be taken at study entry and after the 12 week intervention period; this will be used to assess inflammatory cell infiltrate and the levels of genes and proteins involved in inflammation.

*Subjects and supplements*

# Subjects aged 18 to 65 years with normal weight (a body mass index (BMI) between 18.5 and 25 kg/m^2^ (n = 50)) and obesity (BMI 30 to 40 kg/m^2^ (n = 50) and a waist circumference > 94 cm for men and > 80 cm for women) will be recruited. Subjects will recruited via posters and email shots in the University of Southampton, Southampton General Hospital, and other organisations with which the researchers have contact; and via advertisements in local media including newspapers and radio. Subjects who express an interest will be screened by telephone interview. If they fit the inclusion and exclusion criteria (see below) they will be sent the information sheet. They will be contacted about 7 days later to confirm their interest or not, and if they remain interested an appointment will be made for them to visit the Wellcome Trust Clinical Research Facility at Southampton General Hospital. At this first visit the study will be explained, any questions answered and written consent obtained. Subjects will later make three clinic visits.

The n-3 fatty acid preparation to be used is commercially available as a supplement and will be provided in capsules. Approximately 1.8 g EPA plus DHA will be given during the meal challenge and then will be given per day during the chronic phase of the study. This dose of n-3 fatty acids is similar to that used in many previous studies and is an amount consistent with the Government’s guideline range for oily fish consumption [3]. The placebo capsules will be identical in size and appearance to the fish oil capsules and will contain vegetable oil. Subjects, researchers and clinical staff will be blinded to group allocation.

*Inclusion criteria*

1. Male or female
2. Aged 18 to 65 years
3. Body mass index 18.5 to 25 or 30 to 40 kg/m^2^
4. If body mass index is 30 to 40 kg/m^2^ waist circumference is > 94 cm for men or > 80 cm for women
5. Not consuming fish oil or other oil supplements
6. Not eating more than one oily fish meal per week
7. Willing to adhere to the study protocol
8. Being able to provide written informed consent

*Exclusion criteria*

1. Aged < 18 or > 65 years
2. Body mass index < 18.5, 25-29.9 or > 40 kg/m^2^
3. If body mass index is 30 to 40 kg/m^2^ waist circumference is < 94 cm for men or < 80 cm for women
4. Diagnosed diabetes
5. Use of prescribed medicine to control inflammation
6. Use of prescribed medication to control blood lipids (e.g. statins, fibrates (fenofibrate), Omacor)
7. Use of prescribed medication to control blood pressure (ACE inhibitors, angiotensin 2 receptor blockers, calcium channel blockers, α-inhibitors, thiozide diuretics)
8. Use of fish oil or other oil supplements
9. Chronic gastrointestinal problems (e.g. IBD, celiac disease, cancer)
10. Pregnant or planning to become pregnant within the study period
11. Participation in another clinical trial

# Subject participation schedule

# Subjects who express an interest will be screened by telephone interview. If they fit the inclusion and exclusion criteria they will be sent the information sheet. They will be contacted about 7 days later to confirm their interest or not and if they remain interested an appointment will be made for them to visit the Wellcome Trust Clinical Research Facility, Southampton General Hospital. At this first visit the study will be explained, any questions answered and written consent obtained; subjects will then have height, weight, waist and hip circumference, and body composition (bioelectrical impedance) measured to confirm that they meet the inclusion criteria. A 9 ml blood sample will be taken for preparation of DNA. An appointment will be made for the first clinic visit; subsequently two further clinic visits will be made (i.e. three clinic visits in total after the initial “consenting” visit). Subjects will attend each of these three clinic visits in the fasted state (no food or drink except water after 9 pm the previous evening).

# Subjects will visit the Wellcome Trust Clinical Research Facility, Southampton General Hospital on three occasions each between 8 and 10 am. On each occasion they will be in the fasted state. At each clinic visit weight, waist and hip circumference, and body composition (bioelectrical impedance) will be measured. Two visits will be for post-prandial study days prior to entering the chronic phase of the study. The third visit will be at the end of the chronic phase of the study, at which time a further post-prandial study day will occur. An adipose tissue biopsy will be taken from the subcutaneous abdominal area at the first and third clinic visits.

**First post-prandial day**

**(placebo or fish oil)**

**Second post-prandial day**

**(placebo or fish oil)**

**At least 2 weeks**

**Chronic study**

**(placebo or fish oil)**

**12 weeks duration**

**Third post-prandial day**

**Adipose biopsy;**

**6 blood samples**

**over 6 hours**

**6 blood samples**

**over 6 hours**

**Adipose biopsy;**

**6 blood samples**

**over 6 hours**

# On the first and second post-prandial study days subjects will have a venous cannula inserted and 20 ml blood will be collected at zero time; on the third post-prandial study day an additional 9 ml blood sample will be collected at zero time (this sample will be used for preparation of DNA). They will then consume a standard breakfast (on one occasion with placebo capsules and on the other with fish oil capsules – this will be in random order; on the third post-prandial day only the placebo capsules will be used) and 5 ml blood will be collected at each later time point (1, 2, 3, 4 and 6 hours). Thus 45 ml blood will be collected on each visit (54 ml on the final visit). Blood will be taken into heparin and EDTA as anticoagulants. A small adipose tissue biopsy (weighing 1 to 5 g) will be taken from the subcutaneous abdominal area at the end of the first and third clinic visits; this will be taken under local anaesthetic. At the end of each visit subjects will be offered toast and a hot drink. Subjects will receive a supply of supplements along with instructions on how to take them at the second clinic visit.

As indicated above a small adipose tissue biopsy will be taken at two of the clinic visits. The wound will be stitched and subjects will return to the Wellcome Trust Clinical Research Facility about one week later in order for the stitches to be removed.

# **Schedule of subject visits and activities performed at each visit**

# X indicates the activity will take place.

# Visits 3 and 4 will be about 12 weeks apart and subjects will consume fish oil or placebo capsules daily during that period.

| Activity | Visit 1 | Visit 2  (Clinic visit 1) | Visit 3  (Clinic visit 2) | Visit 4  (Clinic visit 3) |
| --- | --- | --- | --- | --- |
| Subject in fasting state |  | X | X | X |
| Informed consent | X |  |  |  |
| Height | X |  |  |  |
| Weight | X | X | X | X |
| Waist & hip circumference | X | X | X | X |
| Bioelectric impedence | X | X | X | X |
| Blood for DNA | X |  |  | X |
| Post-prandial study: Blood for fatty acids, inflammatory markers and related hormones |  | X | X | X |
| Adipose tissue biopsy |  | X |  | X |

# Sample analysis

# Blood collected at study entry and at the final clinic visit will be used to prepare DNA which will be stored and used later to seek to identify genetic and epigenetic factors (for example polymorphisms in genes related to lipid metabolism or inflammation) that might explain differences among individuals seen in the study. Adipose tissue biopsy material will be stored frozen at minus 80^o^C. Blood will be used to prepare plasma, mononuclear cells and red cells. Plasma will be aliquoted and frozen at minus 80^o^C. Mononuclear cells and red cells will be frozen at minus 80^o^C. Lipid will be extracted from plasma and red cells using chloroform/methanol and fatty acid composition determined using standard techniques. Inflammatory proteins in plasma will be measured by flow cytometry and ELISA. Lipid mediators of inflammation will be measured by mass spectrometry. Inflammatory proteins on the surface of mononuclear cells will be determined by flow cytometry. Inflammatory genes and proteins within mononuclear cells and adipose tissue will be determined by molecular techniques and flow cytometry. Inflammatory cells within adipose tissue will be determined by immunohistochemistry.

# Data will be compared across time and between groups by ANOVA with repeated measures.

**10. Data handling and record keeping**

- All data will be entered onto a spreadsheet (Microsoft Excel) by the researchers involved.
- All data will be entered on a password-protected computer. This data will be accessed only by the PI and the researchers involved.
- All data will only be linked to study codes and thus not identifiable with the source volunteer. However, the caveat to this will be a data set recording the volunteer name and study code without any other volunteer details.
- All data recorded on paper will be kept in a locked filing cabinet in the researchers’ office and/or in a dedicated, restricted access, clinical data storage area on Level D of the IDS Building, University of Southampton.
- Data of an identifiable nature (i.e. volunteer names, contact details, addresses) will be destroyed 12 months after the end of the study. All other data will be kept securely for 15 years and then destroyed.
- Data will be obtained, handled and stored in adherence to the principle set out in the Data Protection Act 1998.
- The investigators will permit monitoring, audits, REC and MHRA review (as applicable) and provide direct access to source data and documents.

**11. Statistical Analysis**

The statistical analysis will involve the comparison of inflammatory markers (proteins, lipids etc) and of fatty acids between normal weight and obese subjects and between placebo and n-3 fatty acid treatments. All statistical comparisons will be performed at the end of the study using SPSS version 14.

**12. Sample size calculation**

Sample size is calculated based upon the chronic intervention phase of the study. We have considered the typical means and standard deviations of prototypical inflammatory mediators (such as interleukin-6) that we have measured previously and an anticipated 20% reduction in these concentrations by n-3 fatty acids. A sample size of 20 subjects per treatment group (i.e. placebo; n-3 fatty acid) will be required to detect a 20% difference in inflammatory markers with 80% power at the 5% significance level (p < 0.05). This number is also consistent with the number of subjects studied by others in investigations of post-prandial inflammation [7,8]. Thus, 40 normal weight and 40 obese subjects will be required to be recruited to satisfy the sample size calculation for the chronic phase of the study. To allow for a 20% drop out rate, 50 normal weight and 50 obese subjects will be recruited.

**13. Safety assessments**

The n-3 fatty acid supplement to be used is commercially available and is safe. Two invasive procedures will be used, adipose tissue biopsy and obtaining blood samples. The biopsy will be taken by a trained surgeon with assistance from trained nursing staff and blood samples will be taken by trained nursing staff limiting the likelihood of adverse events related to participation in the study. However if any volunteer reports any untoward medical occurrence this will be recorded on an adverse event or serious adverse event form and the PI informed immediately. If the investigator suspects that a serious adverse event is either a) related to the intervention or b) unexpected, the PI will report the event to the main REC and to a representative of the supplier of the supplement. An adverse or serious adverse event may result in the volunteer wishing to withdraw from the study or being unable to continue with the study schedule. In this case or any other instance in which a subject withdraws or is withdrawn from the study a volunteer withdrawal form will be completed. Where the reason is know to the investigator or is volunteered by the subject this will be recorded on the form. The subject will not be required to give any reason for withdrawing themselves from the study and will not be asked to do so by the investigator.

**14. Stopping/Discontinuation of intervention**

Completion of each subject’s involvement in the study will be when the last blood sample is taken, which will be approx. 15 weeks after the subject entered the study. If there is any reason for discontinuing the intervention prior to its completion the PI will arrange for the research team to inform all volunteers immediately. The PI will also inform the sponsor and the main REC.

**15. Monitoring**

The project will be overseen and monitored by the Southampton University Hospitals Trust R&D Office.

*Steps taken to ensure quality of research*

Standard operating procedures will be developed for all aspects of the study. Staff will be fully trained in all procedures in which they are involved. All activities will conform to local health and safety regulations and staff will be adequately trained in these. Good clinical practice and good laboratory practice will be used throughout the study. Staff involved in blood sampling will be properly trained for this. All study samples will be labelled clearly, uniquely, accurately and durably using distinctive water resistant labels printed via computer. All samples will be tracked. The temperatures of fridges and freezers in which samples are stored will be monitored to ensure proper functioning. All analyses will be conducted to the highest standards. All equipment to be used is modern, in good working order and maintained on service contracts. All pipettes to be used are serviced regularly. All data will be recorded in laboratory notebooks that will be signed off by the PI at regular intervals. Data entry into spreadsheets will be carefully monitored. All data will be stored securely.

**16. Ethical considerations**

The study will involve the participants consuming a supplement over the course of several weeks and providing a series of blood samples. Participants will not be aware of whether they are consuming the placebo or n-3 fatty acid supplement. Participants will be given an information sheet outlining the nature of the study and they will have the opportunity to discuss any issues they may have with the research staff. Participants will most likely be familiar with having blood sampled, but they will not be familiar with adipose tissue biopsy. This will be explained fully and subjects will be provided with a specific information sheet related to this procedure. Trained researchers will address any concerns that the participants may have. If they remain concerned they will be reminded that they can opt out of any procedure at any time.

**17.** This study will be conducted in accordance with approvals from the LREC and the Southampton University Hospitals Trust R&D Office.

**18.** This study will be conducted in compliance with the Research Governance Framework for Health and Social Care, the Medicine for Human Use (Clinical Trials) Regulation 2004 and ICH GCP.

**19. Financial arrangements**

This study is funded by the European Commission.

**20. Indemnity**

University of Southampton insurance will apply; since an NHS Trust will act as study sponsor, CNST may also apply. University of Southampton insurance may also apply where the cause of harm was not due to clinical negligence as covered by CNST.

**21. Reporting and dissemination**

Results will be presented at scientific conferences and published in relevant scientific journals. Study participants will be informed of the findings of the study, and the results of their samples if they so wish.

**References**

- 1. Kris-Etherton, Harris, Appel, [American Heart Association Nutrition Committee](http://www.ncbi.nlm.nih.gov/entrez/query.fcgi?db=pubmed&cmd=Search&term="American+Heart+Association.+Nutrition+Committee"%5BCorporate+Author%5D) (2002) Fish consumption, fish oil, omega-3 fatty acids, and cardiovascular disease. Circulation 106: 2747-57.
  2. Calder and Yaqoob (2009) Understanding omega-3 polyunsaturated fatty acids. Postgraduate Medicine 121: 148-57.
  3. Scientific Advisory Committee on Nutrition/Committee on Toxicity (2004) Advice on fish consumption; benefits and risks. TSO, London.
  4. Calder (2006) N-3 polyunsaturated fatty acids, inflammation, and inflammatory diseases. American Journal of Clinical Nutrition 83: 1505S-1519S.
  5. Tilg H, Moschen AR (2006) Adipocytokines: mediators linking adipose tissue, inflammation and immunity. Nature Reviews Immunology 6: 772-83.
  6. Hansen, Sickelmann , Pietrowsky, et al. (1997) Systemic immune changes following meal intake in humans. American Journal of Physiology 273: R548-53.
  7. Esposito, Ciotola, Sasso, *et al.* (2007) Effect of a single high-fat meal on endothelial function in patients with the metabolic syndrome: role of tumor necrosis factor-alpha. Nutrition Metabolism and Cardiovascular Disease 17: 274-9.
  8. Nappo, Esposito, Cioffi, et al. (2002) Postprandial endothelial activation in healthy subjects and in type 2 diabetic patients: role of fat and carbohydrate meals. Journal of the American College of Cardiology 39: 1145-50.
  9. Glass and Witztum (2001) Atherosclerosis: The road ahead. Cell 104: 503-16
  10. Burdge and Calder (2005) Plasma cytokine response during the postprandial period: a potential causal process in vascular disease? British Journal of Nutrition 93: 3-9.
