# Supplementary figures and images for: Modification of subcutaneous white adipose tissue inflammation by omega-3 fatty acids is limited in human obesity-a double blind, randomised clinical trial
Source: eBioMedicine. 2022 Mar 2;77:103909. doi: 10.1016/j.ebiom.2022.103909 (PMC8894262; doi:10.1016/j.ebiom.2022.103909)

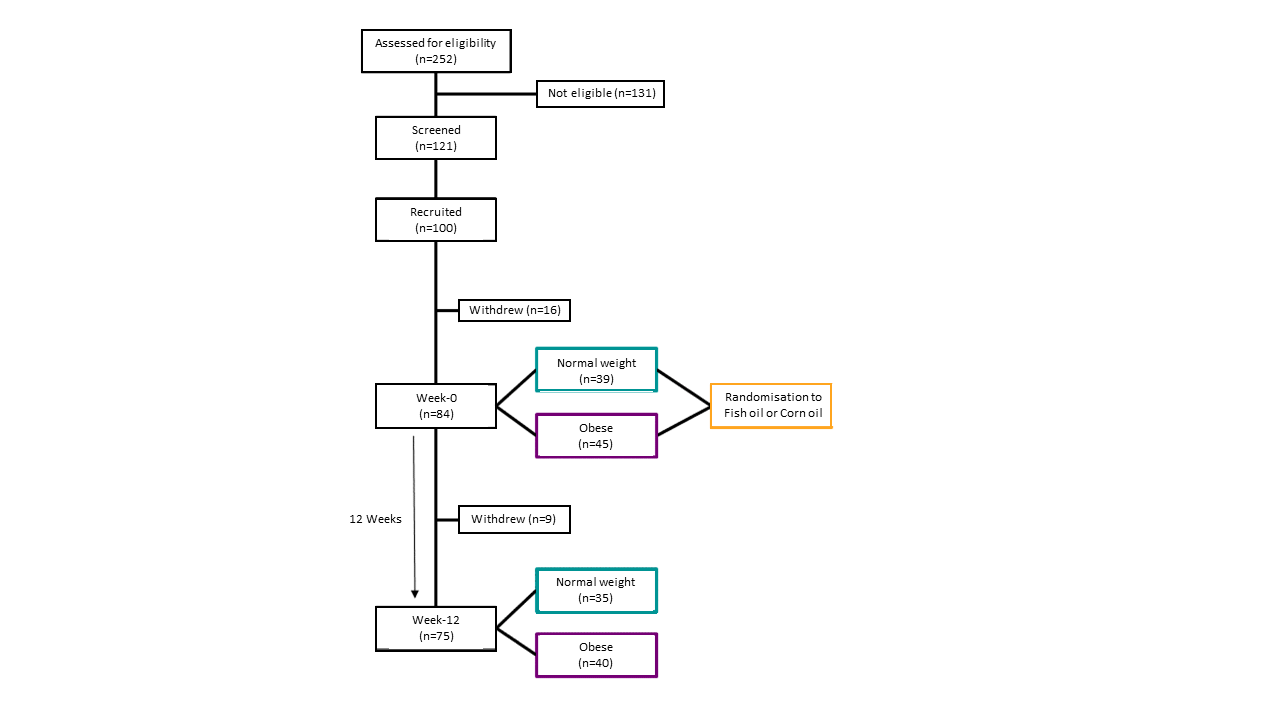

Supplement: Supplementary file 3 [file mmc3.docx]

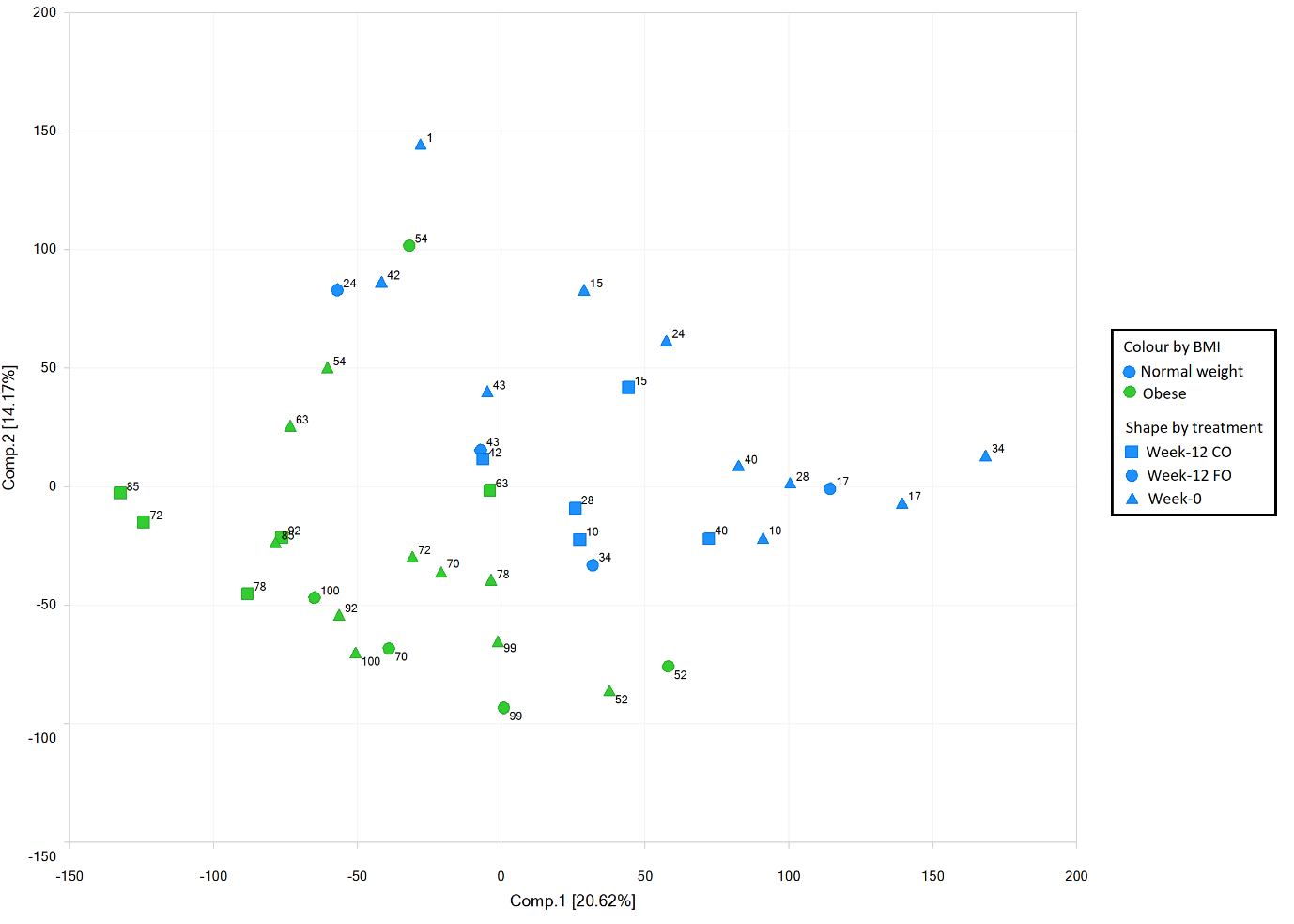

Supplement: Supplementary file 4 [file mmc4.docx]
