# Supplementary material for: Modification of subcutaneous white adipose tissue inflammation by omega-3 fatty acids is limited in human obesity-a double blind, randomised clinical trial
Source: eBioMedicine. 2022 Mar 2;77:103909. doi: 10.1016/j.ebiom.2022.103909 (PMC8894262; doi:10.1016/j.ebiom.2022.103909)
Supplement: Supplementary file 5 [file mmc5.docx]

| **Up-regulated** | | | | |  |  | **Down-regulated** | | | | |
| --- | --- | --- | --- | --- | --- | --- | --- | --- | --- | --- | --- |
| **hgnc_symbol** | **Log2 FC** | **FC** | ***P*** | **FDR** |  |  | **hgnc_symbol** | **Log2 FC** | **FC** | ***P*** | **FDR** |
| EGFL6 | 5.44 | 43.41 | <0.001 | <0.001 |  |  | SLC27A2 | -3.42 | -10.70 | <0.001 | <0.001 |
| MMP7 | 5.41 | 42.52 | <0.001 | <0.001 |  |  | RORB | -3.30 | -9.85 | <0.001 | <0.001 |
| CCL22 | 4.71 | 26.17 | <0.001 | <0.001 |  |  | SPX | -3.26 | -9.58 | <0.001 | <0.001 |
| MMP9 | 4.03 | 16.34 | <0.001 | <0.001 |  |  | CA3 | -3.25 | -9.51 | <0.001 | <0.001 |
| DCSTAMP | 3.91 | 15.03 | <0.001 | <0.001 |  |  | CECR2 | -2.71 | -6.54 | <0.001 | <0.001 |
| URAD | 3.9 | 14.93 | <0.001 | <0.001 |  |  | WDR86-AS1 | -2.56 | -5.90 | <0.001 | <0.001 |
| LINC01010 | 3.88 | 14.72 | <0.001 | <0.001 |  |  | KCNU1 | -2.48 | -5.58 | <0.001 | <0.001 |
| AADACL3 | 3.61 | 12.21 | <0.001 | <0.001 |  |  | ASPG | -2.47 | -5.54 | 0.002 | 0.013 |
| CHIT1 | 3.6 | 12.13 | <0.001 | <0.001 |  |  | BMP3 | -2.45 | -5.46 | <0.001 | <0.001 |
| SPP1 | 3.57 | 11.88 | <0.001 | <0.001 |  |  | RASSF6 | -2.42 | -5.35 | <0.001 | <0.001 |
| PLA2G7 | 3.54 | 11.63 | <0.001 | <0.001 |  |  | TTC36 | -2.41 | -5.31 | <0.001 | <0.001 |
| STMN2 | 3.45 | 10.93 | <0.001 | <0.001 |  |  | GJC3 | -2.31 | -4.96 | <0.001 | <0.001 |
| TREM2 | 3.4 | 10.56 | <0.001 | <0.001 |  |  | KCTD8 | -2.28 | -4.86 | <0.001 | <0.001 |
| SDS | 3.37 | 10.34 | <0.001 | <0.001 |  |  | RPS28 | -2.19 | -4.56 | <0.001 | <0.001 |
| KRT16 | 3.34 | 10.13 | <0.001 | <0.001 |  |  | SCEL | -2.17 | -4.50 | <0.001 | 0.004 |
| UNC13C | 3.33 | 10.06 | <0.001 | <0.001 |  |  | TSHR | -2.17 | -4.50 | <0.001 | <0.001 |
| CHI3L1 | 3.3 | 9.85 | <0.001 | <0.001 |  |  | GFRA3 | -2.07 | -4.20 | <0.001 | <0.001 |
| TM4SF19 | 3.2 | 9.19 | <0.001 | <0.001 |  |  | NDRG4 | -2.02 | -4.06 | <0.001 | <0.001 |
| COL11A1 | 3.07 | 8.40 | <0.001 | <0.001 |  |  | RASL10B | -2.00 | -4.00 | <0.001 | <0.001 |
| IGHV5-51 | 3.04 | 8.22 | <0.001 | <0.001 |  |  | AZGP1 | -1.99 | -3.97 | <0.001 | <0.001 |
| IGLV3-21 | 3.03 | 8.17 | <0.001 | <0.001 |  |  | CSDC2 | -1.94 | -3.84 | <0.001 | <0.001 |
| SLC28A3 | 3 | 8.00 | <0.001 | <0.001 |  |  | LINC01612 | -1.93 | -3.81 | <0.001 | <0.001 |
| IL1RN | 2.97 | 7.84 | <0.001 | <0.001 |  |  | COL6A6 | -1.86 | -3.63 | <0.001 | <0.001 |
| LAMP3 | 2.92 | 7.57 | <0.001 | <0.001 |  |  | PCSK2 | -1.83 | -3.56 | <0.001 | <0.001 |
| SULT1C2 | 2.8 | 6.96 | <0.001 | 0.003 |  |  | MAB21L1 | -1.82 | -3.53 | <0.001 | <0.001 |
| CCL18 | 2.8 | 6.96 | <0.001 | <0.001 |  |  | CIDEA | -1.82 | -3.53 | <0.001 | <0.001 |
| UBE2QL1 | 2.79 | 6.92 | <0.001 | <0.001 |  |  | CHODL | -1.81 | -3.51 | <0.001 | <0.001 |
| IGHV2-5 | 2.74 | 6.68 | <0.001 | 0.001 |  |  | C6 | -1.80 | -3.48 | <0.001 | <0.001 |
| IGLV2-11 | 2.72 | 6.59 | <0.001 | 0.007 |  |  | LINC01230 | -1.77 | -3.41 | <0.001 | <0.001 |
| KIF18B | 2.72 | 6.59 | <0.001 | <0.001 |  |  | LEFTY2 | -1.76 | -3.39 | <0.001 | <0.001 |
| ABCC3 | 2.7 | 6.50 | <0.001 | <0.001 |  |  | CERS3-AS1 | -1.71 | -3.27 | <0.001 | 0.001 |
| DNASE2B | 2.69 | 6.45 | <0.001 | <0.001 |  |  | CPNE4 | -1.71 | -3.27 | <0.001 | <0.001 |
| FCGBP | 2.66 | 6.32 | <0.001 | <0.001 |  |  | DNER | -1.69 | -3.23 | <0.001 | <0.001 |
| TIFAB | 2.63 | 6.19 | <0.001 | <0.001 |  |  | LINC00284 | -1.67 | -3.18 | 0.001 | 0.005 |
| HTRA4 | 2.58 | 5.98 | <0.001 | <0.001 |  |  | LDHC | -1.67 | -3.18 | 0.003 | 0.018 |
| IL4I1 | 2.58 | 5.98 | <0.001 | <0.001 |  |  | USP32P1 | -1.63 | -3.10 | 0.012 | 0.053 |
| GDA | 2.54 | 5.82 | <0.001 | 0.004 |  |  | WDR86 | -1.62 | -3.07 | <0.001 | <0.001 |
| HS3ST2 | 2.52 | 5.74 | <0.001 | <0.001 |  |  | NRXN1 | -1.61 | -3.05 | <0.001 | <0.001 |
| MKI67 | 2.51 | 5.70 | <0.001 | <0.001 |  |  | SPTB | -1.60 | -3.03 | <0.001 | <0.001 |
| IGHG2 | 2.46 | 5.50 | <0.001 | <0.001 |  |  | ABCG8 | -1.59 | -3.01 | <0.001 | 0.004 |
| TCF23 | 2.45 | 5.46 | <0.001 | <0.001 |  |  | DLGAP2 | -1.58 | -2.99 | <0.001 | <0.001 |
| IGLV1-40 | 2.41 | 5.31 | <0.001 | 0.004 |  |  | NCAM2 | -1.58 | -2.99 | <0.001 | <0.001 |
| CLEC5A | 2.41 | 5.31 | <0.001 | <0.001 |  |  | GPAT3 | -1.56 | -2.95 | <0.001 | <0.001 |
| SLC22A12 | 2.4 | 5.28 | <0.001 | 0.005 |  |  | BMP7 | -1.55 | -2.93 | 0.002 | 0.013 |
| COMP | 2.39 | 5.24 | <0.001 | 0.027 |  |  | AADACL2 | -1.55 | -2.93 | 0.009 | 0.042 |
| P2RX6 | 2.38 | 5.21 | <0.001 | <0.001 |  |  | FOXN4 | -1.55 | -2.93 | <0.001 | <0.001 |
| TOP2A | 2.38 | 5.21 | <0.001 | <0.001 |  |  | ALDH1L1-AS2 | -1.55 | -2.93 | <0.001 | <0.001 |
| RGS1 | 2.37 | 5.17 | <0.001 | <0.001 |  |  | SYT17 | -1.55 | -2.93 | <0.001 | <0.001 |
| ANLN | 2.37 | 5.17 | <0.001 | <0.001 |  |  | DKK1 | -1.53 | -2.89 | 0.009 | 0.041 |
| TMEM155 | 2.35 | 5.10 | <0.001 | <0.001 |  |  | MOV10L1 | -1.53 | -2.89 | <0.001 | <0.001 |
| EGR2 | 2.35 | 5.10 | <0.001 | <0.001 |  |  | ADH1A | -1.52 | -2.87 | <0.001 | <0.001 |
| FOSL1 | 2.33 | 5.03 | <0.001 | <0.001 |  |  | XKR4 | -1.52 | -2.87 | <0.001 | <0.001 |
| TNC | 2.32 | 4.99 | <0.001 | <0.001 |  |  | WNT3 | -1.49 | -2.81 | <0.001 | <0.001 |
| IGHG3 | 2.31 | 4.96 | <0.001 | 0.001 |  |  | EYA1 | -1.48 | -2.79 | <0.001 | 0.002 |
| CDC20 | 2.29 | 4.89 | <0.001 | <0.001 |  |  | PGM5-AS1 | -1.48 | -2.79 | <0.001 | <0.001 |
| FCGR1A | 2.28 | 4.86 | <0.001 | <0.001 |  |  | TRIM55 | -1.47 | -2.77 | 0.002 | 0.014 |
| CCL19 | 2.27 | 4.82 | <0.001 | <0.001 |  |  | MAL | -1.46 | -2.75 | <0.001 | <0.001 |
| IGHV3-21 | 2.26 | 4.79 | <0.001 | <0.001 |  |  | DUOX2 | -1.45 | -2.73 | <0.001 | 0.003 |
| LINC00184 | 2.25 | 4.76 | <0.001 | <0.001 |  |  | TMEM52 | -1.45 | -2.73 | <0.001 | <0.001 |
| TEX26 | 2.25 | 4.76 | <0.001 | <0.001 |  |  | GABRD | -1.44 | -2.71 | <0.001 | <0.001 |
| ASPM | 2.25 | 4.76 | <0.001 | <0.001 |  |  | AMN | -1.43 | -2.69 | <0.001 | <0.001 |
| CDCP1 | 2.24 | 4.72 | <0.001 | <0.001 |  |  | RPL27AP | -1.42 | -2.68 | 0.001 | 0.006 |
| MYRFL | 2.21 | 4.63 | <0.001 | <0.001 |  |  | SGCA | -1.42 | -2.68 | 0.005 | 0.027 |
| CHI3L2 | 2.21 | 4.63 | <0.001 | <0.001 |  |  | ADH1B | -1.41 | -2.66 | <0.001 | <0.001 |
| BUB1B | 2.2 | 4.59 | <0.001 | <0.001 |  |  | MKX | -1.40 | -2.64 | <0.001 | 0.002 |
| IGHV3-15 | 2.2 | 4.59 | <0.001 | <0.001 |  |  | ALPK3 | -1.40 | -2.64 | <0.001 | <0.001 |
| TPSD1 | 2.17 | 4.50 | <0.001 | 0.020 |  |  | DUOXA1 | -1.39 | -2.62 | 0.002 | 0.012 |
| LINC00601 | 2.17 | 4.50 | <0.001 | <0.001 |  |  | C19orf33 | -1.39 | -2.62 | <0.001 | <0.001 |
| COL4A2-AS2 | 2.17 | 4.50 | <0.001 | <0.001 |  |  | FHOD3 | -1.38 | -2.60 | <0.001 | 0.002 |
| IGLV2-8 | 2.15 | 4.44 | <0.001 | 0.001 |  |  | CSRP2 | -1.38 | -2.60 | <0.001 | <0.001 |
| C1orf168 | 2.14 | 4.41 | <0.001 | 0.003 |  |  | CYP4F29P | -1.37 | -2.58 | 0.001 | 0.006 |
| CD300E | 2.14 | 4.41 | <0.001 | <0.001 |  |  | CASQ2 | -1.37 | -2.58 | <0.001 | 0.003 |
| GAL | 2.13 | 4.38 | <0.001 | 0.001 |  |  | C8orf89 | -1.37 | -2.58 | <0.001 | 0.003 |
| IGKV1-5 | 2.13 | 4.38 | <0.001 | 0.002 |  |  | CNTD2 | -1.37 | -2.58 | <0.001 | <0.001 |
| SLAMF8 | 2.13 | 4.38 | <0.001 | <0.001 |  |  | MYOC | -1.36 | -2.57 | <0.001 | <0.001 |
| IGHA2 | 2.13 | 4.38 | <0.001 | <0.001 |  |  | ALK | -1.34 | -2.53 | <0.001 | 0.001 |
| LAMC3 | 2.13 | 4.38 | <0.001 | <0.001 |  |  | STOX1 | -1.34 | -2.53 | <0.001 | <0.001 |
| PLEK2 | 2.13 | 4.38 | <0.001 | <0.001 |  |  | MIR181A2HG | -1.34 | -2.53 | <0.001 | <0.001 |
| CD1E | 2.13 | 4.38 | <0.001 | <0.001 |  |  | IZUMO4 | -1.34 | -2.53 | <0.001 | <0.001 |
| IGLV3-1 | 2.12 | 4.35 | <0.001 | 0.014 |  |  | DMRT2 | -1.33 | -2.51 | <0.001 | <0.001 |
| CCL3 | 2.12 | 4.35 | <0.001 | <0.001 |  |  | ARHGEF16 | -1.32 | -2.50 | <0.001 | <0.001 |
| LIPG | 2.12 | 4.35 | <0.001 | <0.001 |  |  | ZBED9 | -1.31 | -2.48 | <0.001 | 0.001 |
| IGHV4-39 | 2.11 | 4.32 | <0.001 | 0.022 |  |  | PGM5P4 | -1.30 | -2.46 | <0.001 | <0.001 |
| AOC1 | 2.11 | 4.32 | <0.001 | <0.001 |  |  | C14orf39 | -1.30 | -2.46 | <0.001 | <0.001 |
| PRND | 2.1 | 4.29 | <0.001 | <0.001 |  |  | CSPG5 | -1.29 | -2.45 | <0.001 | <0.001 |
| IGHV3-23 | 2.09 | 4.26 | <0.001 | <0.001 |  |  | CKB | -1.29 | -2.45 | <0.001 | <0.001 |
| IGLC3 | 2.08 | 4.23 | <0.001 | 0.005 |  |  | TMEM27 | -1.28 | -2.43 | <0.001 | <0.001 |
| KCNK13 | 2.07 | 4.20 | <0.001 | <0.001 |  |  | IGSF11 | -1.27 | -2.41 | 0.011 | 0.047 |
| SIGLEC15 | 2.07 | 4.20 | <0.001 | <0.001 |  |  | FCN2 | -1.27 | -2.41 | <0.001 | <0.001 |
| IGHG1 | 2.06 | 4.17 | <0.001 | 0.005 |  |  | CEBPA-AS1 | -1.25 | -2.38 | 0.036 | 0.116 |
| MXRA5Y | 2.06 | 4.17 | <0.001 | 0.018 |  |  | SOX9-AS1 | -1.25 | -2.38 | <0.001 | 0.002 |
| FAM84A | 2.06 | 4.17 | <0.001 | <0.001 |  |  | CCDC144A | -1.24 | -2.36 | 0.007 | 0.034 |
| FCGR1B | 2.04 | 4.11 | <0.001 | <0.001 |  |  | KRT222 | -1.23 | -2.35 | <0.001 | 0.002 |
| AKR1B15 | 2.03 | 4.08 | <0.001 | <0.001 |  |  | ARF4-AS1 | -1.21 | -2.31 | <0.001 | 0.001 |
| RASGRF1 | 2.03 | 4.08 | <0.001 | <0.001 |  |  | LINC00853 | -1.21 | -2.31 | <0.001 | 0.004 |
| SLC6A12 | 2.02 | 4.06 | <0.001 | <0.001 |  |  | CYB5A | -1.21 | -2.31 | <0.001 | <0.001 |
| FAM111B | 2.02 | 4.06 | <0.001 | <0.001 |  |  | PDE4C | -1.19 | -2.28 | <0.001 | <0.001 |
| MATK | 2.02 | 4.06 | <0.001 | <0.001 |  |  | TMEM25 | -1.19 | -2.28 | <0.001 | <0.001 |
| SERPINE1 | 1.99 | 3.97 | <0.001 | <0.001 |  |  | SULT4A1 | -1.18 | -2.27 | 0.002 | 0.011 |
| BIRC5 | 1.99 | 3.97 | <0.001 | <0.001 |  |  | FAM156A | -1.18 | -2.27 | 0.003 | 0.016 |
| MXRA5 | 1.99 | 3.97 | <0.001 | <0.001 |  |  | DACT2 | -1.18 | -2.27 | 0.010 | 0.046 |
| CD300LB | 1.97 | 3.92 | <0.001 | <0.001 |  |  | RPS27 | -1.18 | -2.27 | <0.001 | <0.001 |
| CKAP2L | 1.96 | 3.89 | <0.001 | 0.002 |  |  | EIF4EBP1 | -1.18 | -2.27 | <0.001 | <0.001 |
| PCSK1 | 1.96 | 3.89 | <0.001 | <0.001 |  |  | PPP1R16A | -1.18 | -2.27 | <0.001 | <0.001 |
| ST14 | 1.96 | 3.89 | <0.001 | <0.001 |  |  | PEX5L | -1.17 | -2.25 | 0.003 | 0.016 |
| TREML1 | 1.94 | 3.84 | <0.001 | <0.001 |  |  | TBX4 | -1.17 | -2.25 | 0.004 | 0.025 |
| CLEC12A | 1.93 | 3.81 | <0.001 | <0.001 |  |  | PXDNL | -1.17 | -2.25 | 0.009 | 0.043 |
| IFI30 | 1.93 | 3.81 | <0.001 | <0.001 |  |  | HRCT1 | -1.17 | -2.25 | <0.001 | 0.002 |
| DLGAP5 | 1.93 | 3.81 | <0.001 | <0.001 |  |  | AACS | -1.17 | -2.25 | <0.001 | <0.001 |
| SPTA1 | 1.93 | 3.81 | <0.001 | <0.001 |  |  | FADS1 | -1.17 | -2.25 | <0.001 | <0.001 |
| MYCL | 1.92 | 3.78 | <0.001 | <0.001 |  |  | RPL37P6 | -1.17 | -2.25 | <0.001 | <0.001 |
| CCR5 | 1.91 | 3.76 | <0.001 | <0.001 |  |  | RORC | -1.17 | -2.25 | <0.001 | <0.001 |
| HLA-DQB1-AS1 | 1.9 | 3.73 | <0.001 | 0.013 |  |  | S100A1 | -1.16 | -2.23 | 0.012 | 0.052 |
| IGHA1 | 1.9 | 3.73 | <0.001 | <0.001 |  |  | ZNF334 | -1.16 | -2.23 | <0.001 | <0.001 |
| ITGAD | 1.9 | 3.73 | <0.001 | <0.001 |  |  | SLC22A25 | -1.16 | -2.23 | <0.001 | <0.001 |
| SPOCD1 | 1.9 | 3.73 | <0.001 | <0.001 |  |  | SCN7A | -1.16 | -2.23 | <0.001 | <0.001 |
| SPIB | 1.9 | 3.73 | <0.001 | <0.001 |  |  | HACD1 | -1.16 | -2.23 | <0.001 | <0.001 |
| CEP55 | 1.88 | 3.68 | <0.001 | <0.001 |  |  | RARB | -1.16 | -2.23 | <0.001 | <0.001 |
| SCIN | 1.88 | 3.68 | <0.001 | <0.001 |  |  | RPL41P2 | -1.15 | -2.22 | 0.001 | 0.007 |
| SPINK5 | 1.87 | 3.66 | <0.001 | 0.002 |  |  | GRIK3 | -1.15 | -2.22 | 0.002 | 0.014 |
| IGHGP | 1.87 | 3.66 | <0.001 | 0.047 |  |  | ARL17B | -1.15 | -2.22 | 0.003 | 0.019 |
| IGKV3-20 | 1.87 | 3.66 | <0.001 | <0.001 |  |  | SCN2B | -1.15 | -2.22 | 0.007 | 0.035 |
| HJURP | 1.87 | 3.66 | <0.001 | <0.001 |  |  | PLD6 | -1.15 | -2.22 | <0.001 | 0.001 |
| ITGAX | 1.87 | 3.66 | <0.001 | <0.001 |  |  | ADSSL1 | -1.15 | -2.22 | <0.001 | <0.001 |
| KRT7 | 1.86 | 3.63 | <0.001 | <0.001 |  |  | TRDC | -1.15 | -2.22 | <0.001 | <0.001 |
| MMP12 | 1.85 | 3.61 | <0.001 | <0.001 |  |  | ALB | -1.14 | -2.20 | 0.001 | 0.007 |
| LGALS2 | 1.85 | 3.61 | <0.001 | <0.001 |  |  | APOL4 | -1.14 | -2.20 | 0.002 | 0.015 |
| IGKV4-1 | 1.84 | 3.58 | <0.001 | 0.022 |  |  | PCK1 | -1.13 | -2.19 | 0.001 | 0.005 |
| HLA-DQA1 | 1.84 | 3.58 | <0.001 | <0.001 |  |  | AK5 | -1.13 | -2.19 | 0.005 | 0.028 |
| TTK | 1.84 | 3.58 | <0.001 | <0.001 |  |  | ZP1 | -1.13 | -2.19 | <0.001 | <0.001 |
| ZNF804A | 1.83 | 3.56 | <0.001 | <0.001 |  |  | ANKRD53 | -1.13 | -2.19 | <0.001 | <0.001 |
| CYP2S1 | 1.82 | 3.53 | <0.001 | <0.001 |  |  | GLUL | -1.13 | -2.19 | <0.001 | <0.001 |
| CLEC12B | 1.82 | 3.53 | <0.001 | <0.001 |  |  | NAALAD2 | -1.13 | -2.19 | <0.001 | <0.001 |
| IGKC | 1.81 | 3.51 | <0.001 | 0.001 |  |  | SLC25A21 | -1.13 | -2.19 | <0.001 | <0.001 |
| HLA-DQA2 | 1.81 | 3.51 | <0.001 | 0.041 |  |  | SMIM1 | -1.13 | -2.19 | <0.001 | <0.001 |
| CCR2 | 1.81 | 3.51 | <0.001 | <0.001 |  |  | NTRK3 | -1.12 | -2.17 | 0.001 | 0.010 |
| IGHM | 1.81 | 3.51 | <0.001 | <0.001 |  |  | PLIN5 | -1.12 | -2.17 | <0.001 | <0.001 |
| TCHH | 1.8 | 3.48 | <0.001 | <0.001 |  |  | ANXA3 | -1.12 | -2.17 | <0.001 | <0.001 |
| RRM2 | 1.8 | 3.48 | <0.001 | <0.001 |  |  | PPP1R14A | -1.12 | -2.17 | <0.001 | <0.001 |
| GRIN2B | 1.8 | 3.48 | <0.001 | <0.001 |  |  | OR7E13P | -1.11 | -2.16 | 0.001 | 0.007 |
| KCNJ1 | 1.8 | 3.48 | <0.001 | <0.001 |  |  | GRIK4 | -1.11 | -2.16 | 0.002 | 0.015 |
| KIAA0125 | 1.8 | 3.48 | <0.001 | <0.001 |  |  | GCK | -1.11 | -2.16 | 0.005 | 0.028 |
| LBP | 1.79 | 3.46 | <0.001 | <0.001 |  |  | S100P | -1.10 | -2.14 | 0.004 | 0.024 |
| LINC00968 | 1.79 | 3.46 | <0.001 | <0.001 |  |  | C11orf53 | -1.10 | -2.14 | 0.006 | 0.032 |
| JCHAIN | 1.78 | 3.43 | <0.001 | 0.001 |  |  | RPS27P23 | -1.10 | -2.14 | <0.001 | 0.003 |
| BCL11A | 1.78 | 3.43 | <0.001 | <0.001 |  |  | PXMP2 | -1.10 | -2.14 | <0.001 | <0.001 |
| CXCL1 | 1.77 | 3.41 | <0.001 | 0.001 |  |  | KRBOX1 | -1.07 | -2.10 | 0.002 | 0.013 |
| UBE2C | 1.75 | 3.36 | <0.001 | 0.001 |  |  | GCHFR | -1.07 | -2.10 | <0.001 | <0.001 |
| CLIC6 | 1.75 | 3.36 | <0.001 | 0.024 |  |  | KANSL1-AS1 | -1.07 | -2.10 | <0.001 | <0.001 |
| UCHL1 | 1.75 | 3.36 | <0.001 | <0.001 |  |  | C9orf170 | -1.06 | -2.08 | 0.009 | 0.041 |
| CECR1 | 1.75 | 3.36 | <0.001 | <0.001 |  |  | ERBB3 | -1.06 | -2.08 | <0.001 | 0.004 |
| CD1C | 1.75 | 3.36 | <0.001 | <0.001 |  |  | RBPMS-AS1 | -1.06 | -2.08 | <0.001 | <0.001 |
| ASPHD1 | 1.74 | 3.34 | <0.001 | <0.001 |  |  | DPH6-AS1 | -1.05 | -2.07 | 0.004 | 0.023 |
| SLAMF7 | 1.74 | 3.34 | <0.001 | <0.001 |  |  | MT-ND3 | -1.05 | -2.07 | <0.001 | <0.001 |
| ANKRD30B | 1.73 | 3.32 | <0.001 | 0.001 |  |  | PCBD1 | -1.05 | -2.07 | <0.001 | <0.001 |
| COL4A4 | 1.72 | 3.29 | <0.001 | <0.001 |  |  | SHOX2 | -1.04 | -2.06 | 0.001 | 0.005 |
| FLT3 | 1.72 | 3.29 | <0.001 | <0.001 |  |  | NWD2 | -1.04 | -2.06 | 0.001 | 0.007 |
| ALCAM | 1.71 | 3.27 | <0.001 | <0.001 |  |  | FAM46B | -1.04 | -2.06 | 0.002 | 0.011 |
| GPR39 | 1.71 | 3.27 | <0.001 | <0.001 |  |  | ARHGEF26-AS1 | -1.04 | -2.06 | <0.001 | 0.003 |
| MSR1 | 1.7 | 3.25 | <0.001 | <0.001 |  |  | GLYCTK | -1.04 | -2.06 | <0.001 | <0.001 |
| AATBC | 1.7 | 3.25 | <0.001 | <0.001 |  |  | MPPED2 | -1.04 | -2.06 | <0.001 | <0.001 |
| AURKB | 1.69 | 3.23 | <0.001 | 0.004 |  |  | PLAG1 | -1.04 | -2.06 | <0.001 | <0.001 |
| CXCL8 | 1.69 | 3.23 | <0.001 | 0.025 |  |  | SLC16A11 | -1.04 | -2.06 | <0.001 | <0.001 |
| U2AF1 | 1.69 | 3.23 | <0.001 | 0.034 |  |  | ADAM20P1 | -1.02 | -2.03 | <0.001 | 0.002 |
| CASC5 | 1.69 | 3.23 | <0.001 | <0.001 |  |  | NIPSNAP3B | -1.02 | -2.03 | <0.001 | <0.001 |
| KMO | 1.69 | 3.23 | <0.001 | <0.001 |  |  | ASIC4 | -1.01 | -2.01 | 0.005 | 0.025 |
| TRDN | 1.68 | 3.20 | <0.001 | 0.008 |  |  | PKP2 | -1.01 | -2.01 | 0.006 | 0.031 |
| AOAH | 1.68 | 3.20 | <0.001 | <0.001 |  |  | RPL9P9 | -1.01 | -2.01 | <0.001 | 0.002 |
| NPR3 | 1.68 | 3.20 | <0.001 | <0.001 |  |  | PLPP2 | -1.01 | -2.01 | <0.001 | 0.004 |
| RPL7AP64 | 1.67 | 3.18 | <0.001 | 0.001 |  |  | DMKN | -1.01 | -2.01 | <0.001 | <0.001 |
| SEZ6 | 1.67 | 3.18 | <0.001 | 0.013 |  |  | IGFBP6 | -1.01 | -2.01 | <0.001 | <0.001 |
| UCKL1-AS1 | 1.67 | 3.18 | <0.001 | <0.001 |  |  | COL9A3 | -1.00 | -2.00 | 0.001 | 0.007 |
| PRAM1 | 1.67 | 3.18 | <0.001 | <0.001 |  |  | RPL31P61 | -1.00 | -2.00 | 0.001 | 0.009 |
| FCN1 | 1.66 | 3.16 | <0.001 | <0.001 |  |  | CDH19 | -1.00 | -2.00 | 0.003 | 0.020 |
| LYZ | 1.66 | 3.16 | <0.001 | <0.001 |  |  | GPX3 | -1.00 | -2.00 | <0.001 | 0.002 |
| IGHG4 | 1.65 | 3.14 | <0.001 | 0.009 |  |  | DCXR | -1.00 | -2.00 | <0.001 | <0.001 |
| HMMR | 1.65 | 3.14 | <0.001 | <0.001 |  |  |  |  |  |  |  |
| PAX3 | 1.64 | 3.12 | <0.001 | 0.010 |  |  |  |  |  |  |  |
| TUBB2B | 1.64 | 3.12 | <0.001 | <0.001 |  |  |  |  |  |  |  |
| IQGAP3 | 1.64 | 3.12 | <0.001 | <0.001 |  |  |  |  |  |  |  |
| SAPCD2 | 1.63 | 3.10 | <0.001 | 0.002 |  |  |  |  |  |  |  |
| MZB1 | 1.63 | 3.10 | <0.001 | 0.004 |  |  |  |  |  |  |  |
| CLEC7A | 1.63 | 3.10 | <0.001 | <0.001 |  |  |  |  |  |  |  |
| KIF2C | 1.63 | 3.10 | <0.001 | <0.001 |  |  |  |  |  |  |  |
| NME8 | 1.63 | 3.10 | <0.001 | <0.001 |  |  |  |  |  |  |  |
| SAA2 | 1.62 | 3.07 | <0.001 | 0.040 |  |  |  |  |  |  |  |
| LCP1 | 1.62 | 3.07 | <0.001 | <0.001 |  |  |  |  |  |  |  |
| LILRA4 | 1.62 | 3.07 | <0.001 | <0.001 |  |  |  |  |  |  |  |
| THBS1 | 1.62 | 3.07 | <0.001 | <0.001 |  |  |  |  |  |  |  |
| GABRG1 | 1.62 | 3.07 | <0.001 | <0.001 |  |  |  |  |  |  |  |
| TENM4 | 1.61 | 3.05 | <0.001 | <0.001 |  |  |  |  |  |  |  |
| JAKMIP3 | 1.61 | 3.05 | <0.001 | <0.001 |  |  |  |  |  |  |  |
| JPH2 | 1.6 | 3.03 | <0.001 | 0.005 |  |  |  |  |  |  |  |
| PDE6G | 1.6 | 3.03 | <0.001 | <0.001 |  |  |  |  |  |  |  |
| ADGRE4P | 1.6 | 3.03 | <0.001 | <0.001 |  |  |  |  |  |  |  |
| ACP5 | 1.6 | 3.03 | <0.001 | <0.001 |  |  |  |  |  |  |  |
| APLN | 1.6 | 3.03 | <0.001 | <0.001 |  |  |  |  |  |  |  |
| CXCL9 | 1.59 | 3.01 | <0.001 | 0.005 |  |  |  |  |  |  |  |
| CLCA2 | 1.59 | 3.01 | <0.001 | <0.001 |  |  |  |  |  |  |  |
| IGFN1 | 1.59 | 3.01 | <0.001 | <0.001 |  |  |  |  |  |  |  |
| WDFY4 | 1.58 | 2.99 | <0.001 | <0.001 |  |  |  |  |  |  |  |
| KCNC3 | 1.58 | 2.99 | <0.001 | <0.001 |  |  |  |  |  |  |  |
| NUGGC | 1.58 | 2.99 | <0.001 | <0.001 |  |  |  |  |  |  |  |
| ADCYAP1 | 1.57 | 2.97 | <0.001 | 0.017 |  |  |  |  |  |  |  |
| DPP6 | 1.57 | 2.97 | <0.001 | 0.022 |  |  |  |  |  |  |  |
| RANBP3L | 1.57 | 2.97 | <0.001 | <0.001 |  |  |  |  |  |  |  |
| IGLV2-14 | 1.56 | 2.95 | <0.001 | 0.005 |  |  |  |  |  |  |  |
| CENPF | 1.56 | 2.95 | <0.001 | <0.001 |  |  |  |  |  |  |  |
| LINC01094 | 1.56 | 2.95 | <0.001 | <0.001 |  |  |  |  |  |  |  |
| APOBR | 1.56 | 2.95 | <0.001 | <0.001 |  |  |  |  |  |  |  |
| KIF14 | 1.55 | 2.93 | <0.001 | 0.002 |  |  |  |  |  |  |  |
| C5orf64 | 1.55 | 2.93 | <0.001 | <0.001 |  |  |  |  |  |  |  |
| GABRB2 | 1.55 | 2.93 | <0.001 | <0.001 |  |  |  |  |  |  |  |
| CTSV | 1.55 | 2.93 | <0.001 | <0.001 |  |  |  |  |  |  |  |
| CLSPN | 1.53 | 2.89 | <0.001 | 0.001 |  |  |  |  |  |  |  |
| TMC5 | 1.53 | 2.89 | <0.001 | 0.028 |  |  |  |  |  |  |  |
| PROSER2-AS1 | 1.53 | 2.89 | <0.001 | <0.001 |  |  |  |  |  |  |  |
| FAM225A | 1.53 | 2.89 | <0.001 | <0.001 |  |  |  |  |  |  |  |
| CACNG8 | 1.53 | 2.89 | <0.001 | <0.001 |  |  |  |  |  |  |  |
| MSC | 1.53 | 2.89 | <0.001 | <0.001 |  |  |  |  |  |  |  |
| IGKV3-11 | 1.52 | 2.87 | <0.001 | 0.029 |  |  |  |  |  |  |  |
| CXCL10 | 1.52 | 2.87 | <0.001 | 0.045 |  |  |  |  |  |  |  |
| SPINT1 | 1.52 | 2.87 | <0.001 | <0.001 |  |  |  |  |  |  |  |
| ITGB2 | 1.52 | 2.87 | <0.001 | <0.001 |  |  |  |  |  |  |  |
| HP | 1.51 | 2.85 | <0.001 | 0.004 |  |  |  |  |  |  |  |
| IGLC2 | 1.51 | 2.85 | <0.001 | 0.006 |  |  |  |  |  |  |  |
| PLD4 | 1.51 | 2.85 | <0.001 | <0.001 |  |  |  |  |  |  |  |
| TTYH3 | 1.51 | 2.85 | <0.001 | <0.001 |  |  |  |  |  |  |  |
| NR4A3 | 1.51 | 2.85 | <0.001 | <0.001 |  |  |  |  |  |  |  |
| DNAH10 | 1.51 | 2.85 | <0.001 | <0.001 |  |  |  |  |  |  |  |
| WWC1 | 1.51 | 2.85 | <0.001 | <0.001 |  |  |  |  |  |  |  |
| CD52 | 1.51 | 2.85 | <0.001 | <0.001 |  |  |  |  |  |  |  |
| CNTN6 | 1.5 | 2.83 | <0.001 | 0.002 |  |  |  |  |  |  |  |
| MFSD2A | 1.5 | 2.83 | <0.001 | <0.001 |  |  |  |  |  |  |  |
| SPN | 1.5 | 2.83 | <0.001 | <0.001 |  |  |  |  |  |  |  |
| ARPP21 | 1.5 | 2.83 | <0.001 | <0.001 |  |  |  |  |  |  |  |
| CLEC4D | 1.49 | 2.81 | <0.001 | 0.053 |  |  |  |  |  |  |  |
| LY9 | 1.49 | 2.81 | <0.001 | <0.001 |  |  |  |  |  |  |  |
| LINC01503 | 1.49 | 2.81 | <0.001 | <0.001 |  |  |  |  |  |  |  |
| GSG2 | 1.49 | 2.81 | <0.001 | <0.001 |  |  |  |  |  |  |  |
| LINC00877 | 1.49 | 2.81 | <0.001 | <0.001 |  |  |  |  |  |  |  |
| FPR3 | 1.49 | 2.81 | <0.001 | <0.001 |  |  |  |  |  |  |  |
| ESCO2 | 1.48 | 2.79 | <0.001 | 0.002 |  |  |  |  |  |  |  |
| HLA-DQB1 | 1.48 | 2.79 | <0.001 | 0.020 |  |  |  |  |  |  |  |
| KCNN4 | 1.48 | 2.79 | <0.001 | <0.001 |  |  |  |  |  |  |  |
| SIGLEC9 | 1.48 | 2.79 | <0.001 | <0.001 |  |  |  |  |  |  |  |
| XCR1 | 1.47 | 2.77 | <0.001 | 0.001 |  |  |  |  |  |  |  |
| MIR4767 | 1.47 | 2.77 | <0.001 | <0.001 |  |  |  |  |  |  |  |
| OSCAR | 1.47 | 2.77 | <0.001 | <0.001 |  |  |  |  |  |  |  |
| IL6 | 1.46 | 2.75 | <0.001 | 0.002 |  |  |  |  |  |  |  |
| CP | 1.46 | 2.75 | <0.001 | <0.001 |  |  |  |  |  |  |  |
| LINC00942 | 1.45 | 2.73 | <0.001 | 0.015 |  |  |  |  |  |  |  |
| ANKRD30A | 1.45 | 2.73 | <0.001 | <0.001 |  |  |  |  |  |  |  |
| ACSM4 | 1.44 | 2.71 | <0.001 | 0.001 |  |  |  |  |  |  |  |
| HLA-DQB2 | 1.44 | 2.71 | <0.001 | 0.008 |  |  |  |  |  |  |  |
| ACPP | 1.44 | 2.71 | <0.001 | <0.001 |  |  |  |  |  |  |  |
| CCR4 | 1.44 | 2.71 | <0.001 | <0.001 |  |  |  |  |  |  |  |
| TROAP | 1.43 | 2.69 | <0.001 | 0.002 |  |  |  |  |  |  |  |
| SULT1A2 | 1.42 | 2.68 | <0.001 | 0.001 |  |  |  |  |  |  |  |
| CELSR1 | 1.42 | 2.68 | <0.001 | 0.001 |  |  |  |  |  |  |  |
| GPR141 | 1.42 | 2.68 | <0.001 | <0.001 |  |  |  |  |  |  |  |
| BRCA2 | 1.42 | 2.68 | <0.001 | <0.001 |  |  |  |  |  |  |  |
| PAQR5 | 1.42 | 2.68 | <0.001 | <0.001 |  |  |  |  |  |  |  |
| CFB | 1.42 | 2.68 | <0.001 | <0.001 |  |  |  |  |  |  |  |
| PRG4 | 1.41 | 2.66 | <0.001 | 0.010 |  |  |  |  |  |  |  |
| C6orf183 | 1.41 | 2.66 | <0.001 | <0.001 |  |  |  |  |  |  |  |
| NCAPH | 1.41 | 2.66 | <0.001 | <0.001 |  |  |  |  |  |  |  |
| SERINC2 | 1.41 | 2.66 | <0.001 | <0.001 |  |  |  |  |  |  |  |
| GDF15 | 1.4 | 2.64 | <0.001 | 0.001 |  |  |  |  |  |  |  |
| P2RX6P | 1.4 | 2.64 | <0.001 | 0.004 |  |  |  |  |  |  |  |
| CDHR5 | 1.4 | 2.64 | <0.001 | 0.029 |  |  |  |  |  |  |  |
| ADGRE2 | 1.4 | 2.64 | <0.001 | <0.001 |  |  |  |  |  |  |  |
| LINC01272 | 1.4 | 2.64 | <0.001 | <0.001 |  |  |  |  |  |  |  |
| MELK | 1.4 | 2.64 | <0.001 | <0.001 |  |  |  |  |  |  |  |
| KCNAB2 | 1.4 | 2.64 | <0.001 | <0.001 |  |  |  |  |  |  |  |
| TPX2 | 1.4 | 2.64 | <0.001 | <0.001 |  |  |  |  |  |  |  |
| MYH2 | 1.39 | 2.62 | <0.001 | 0.001 |  |  |  |  |  |  |  |
| RGS20 | 1.39 | 2.62 | <0.001 | <0.001 |  |  |  |  |  |  |  |
| SYNPO | 1.39 | 2.62 | <0.001 | <0.001 |  |  |  |  |  |  |  |
| CYP19A1 | 1.39 | 2.62 | <0.001 | <0.001 |  |  |  |  |  |  |  |
| LINC01347 | 1.39 | 2.62 | <0.001 | <0.001 |  |  |  |  |  |  |  |
| HPD | 1.39 | 2.62 | <0.001 | <0.001 |  |  |  |  |  |  |  |
| CHST1 | 1.39 | 2.62 | <0.001 | <0.001 |  |  |  |  |  |  |  |
| KCNQ3 | 1.39 | 2.62 | <0.001 | <0.001 |  |  |  |  |  |  |  |
| DCHS2 | 1.38 | 2.60 | <0.001 | 0.001 |  |  |  |  |  |  |  |
| LINC01021 | 1.38 | 2.60 | <0.001 | 0.023 |  |  |  |  |  |  |  |
| DHRS9 | 1.37 | 2.58 | <0.001 | <0.001 |  |  |  |  |  |  |  |
| FAT2 | 1.37 | 2.58 | <0.001 | <0.001 |  |  |  |  |  |  |  |
| SLC4A3 | 1.37 | 2.58 | <0.001 | <0.001 |  |  |  |  |  |  |  |
| B3GNT7 | 1.37 | 2.58 | <0.001 | <0.001 |  |  |  |  |  |  |  |
| CIITA | 1.37 | 2.58 | <0.001 | <0.001 |  |  |  |  |  |  |  |
| CYP27B1 | 1.36 | 2.57 | <0.001 | 0.002 |  |  |  |  |  |  |  |
| SLC34A3 | 1.36 | 2.57 | <0.001 | 0.015 |  |  |  |  |  |  |  |
| TRPC4 | 1.36 | 2.57 | <0.001 | 0.028 |  |  |  |  |  |  |  |
| TLR8 | 1.36 | 2.57 | <0.001 | <0.001 |  |  |  |  |  |  |  |
| NUF2 | 1.35 | 2.55 | <0.001 | 0.005 |  |  |  |  |  |  |  |
| CD300LF | 1.35 | 2.55 | <0.001 | <0.001 |  |  |  |  |  |  |  |
| POU2F2 | 1.35 | 2.55 | <0.001 | <0.001 |  |  |  |  |  |  |  |
| TLR7 | 1.35 | 2.55 | <0.001 | <0.001 |  |  |  |  |  |  |  |
| SIGLEC14 | 1.34 | 2.53 | <0.001 | 0.002 |  |  |  |  |  |  |  |
| CCNB2 | 1.34 | 2.53 | <0.001 | <0.001 |  |  |  |  |  |  |  |
| SYNC | 1.34 | 2.53 | <0.001 | <0.001 |  |  |  |  |  |  |  |
| NGFR | 1.34 | 2.53 | <0.001 | <0.001 |  |  |  |  |  |  |  |
| PHLDA2 | 1.33 | 2.51 | <0.001 | 0.003 |  |  |  |  |  |  |  |
| USP2 | 1.33 | 2.51 | <0.001 | <0.001 |  |  |  |  |  |  |  |
| MYBPC3 | 1.32 | 2.50 | <0.001 | 0.001 |  |  |  |  |  |  |  |
| CLECL1 | 1.32 | 2.50 | <0.001 | 0.026 |  |  |  |  |  |  |  |
| FCMR | 1.32 | 2.50 | <0.001 | <0.001 |  |  |  |  |  |  |  |
| CD300C | 1.32 | 2.50 | <0.001 | <0.001 |  |  |  |  |  |  |  |
| NOD2 | 1.32 | 2.50 | <0.001 | <0.001 |  |  |  |  |  |  |  |
| LAMA5-AS1 | 1.32 | 2.50 | <0.001 | <0.001 |  |  |  |  |  |  |  |
| CACNA2D1 | 1.31 | 2.48 | <0.001 | <0.001 |  |  |  |  |  |  |  |
| HLA-DOA | 1.31 | 2.48 | <0.001 | <0.001 |  |  |  |  |  |  |  |
| SLC7A5 | 1.31 | 2.48 | <0.001 | <0.001 |  |  |  |  |  |  |  |
| KCNA3 | 1.3 | 2.46 | <0.001 | 0.001 |  |  |  |  |  |  |  |
| ASGR2 | 1.3 | 2.46 | <0.001 | 0.001 |  |  |  |  |  |  |  |
| KIF20A | 1.3 | 2.46 | <0.001 | 0.008 |  |  |  |  |  |  |  |
| JAKMIP2 | 1.3 | 2.46 | <0.001 | 0.008 |  |  |  |  |  |  |  |
| ADAMTS19 | 1.3 | 2.46 | <0.001 | 0.047 |  |  |  |  |  |  |  |
| SLC9A7 | 1.3 | 2.46 | <0.001 | <0.001 |  |  |  |  |  |  |  |
| PALM2-AKAP2 | 1.3 | 2.46 | <0.001 | <0.001 |  |  |  |  |  |  |  |
| SCIMP | 1.3 | 2.46 | <0.001 | <0.001 |  |  |  |  |  |  |  |
| TM4SF19-AS1 | 1.3 | 2.46 | <0.001 | <0.001 |  |  |  |  |  |  |  |
| NCEH1 | 1.3 | 2.46 | <0.001 | <0.001 |  |  |  |  |  |  |  |
| PANX2 | 1.3 | 2.46 | <0.001 | <0.001 |  |  |  |  |  |  |  |
| NUP210 | 1.3 | 2.46 | <0.001 | <0.001 |  |  |  |  |  |  |  |
| CENPE | 1.3 | 2.46 | <0.001 | <0.001 |  |  |  |  |  |  |  |
| LUCAT1 | 1.29 | 2.45 | <0.001 | 0.002 |  |  |  |  |  |  |  |
| CFAP221 | 1.29 | 2.45 | <0.001 | 0.002 |  |  |  |  |  |  |  |
| NFE4 | 1.29 | 2.45 | <0.001 | 0.003 |  |  |  |  |  |  |  |
| COL4A3 | 1.29 | 2.45 | <0.001 | 0.028 |  |  |  |  |  |  |  |
| PLA2G2D | 1.29 | 2.45 | <0.001 | <0.001 |  |  |  |  |  |  |  |
| TGM1 | 1.29 | 2.45 | <0.001 | <0.001 |  |  |  |  |  |  |  |
| LTBP2 | 1.29 | 2.45 | <0.001 | <0.001 |  |  |  |  |  |  |  |
| NETO2 | 1.29 | 2.45 | <0.001 | <0.001 |  |  |  |  |  |  |  |
| NCR3LG1 | 1.29 | 2.45 | <0.001 | <0.001 |  |  |  |  |  |  |  |
| HLA-DRA | 1.29 | 2.45 | <0.001 | <0.001 |  |  |  |  |  |  |  |
| SIGLEC7 | 1.29 | 2.45 | <0.001 | <0.001 |  |  |  |  |  |  |  |
| DIAPH3 | 1.28 | 2.43 | <0.001 | 0.001 |  |  |  |  |  |  |  |
| IL21R | 1.28 | 2.43 | <0.001 | 0.001 |  |  |  |  |  |  |  |
| TFCP2L1 | 1.28 | 2.43 | <0.001 | 0.002 |  |  |  |  |  |  |  |
| HTR7 | 1.28 | 2.43 | <0.001 | 0.007 |  |  |  |  |  |  |  |
| TRGV5 | 1.28 | 2.43 | <0.001 | 0.029 |  |  |  |  |  |  |  |
| DEPDC1 | 1.28 | 2.43 | <0.001 | 0.030 |  |  |  |  |  |  |  |
| CD163 | 1.28 | 2.43 | <0.001 | <0.001 |  |  |  |  |  |  |  |
| SIRPB1 | 1.27 | 2.41 | <0.001 | 0.001 |  |  |  |  |  |  |  |
| KLHDC7B | 1.27 | 2.41 | <0.001 | 0.001 |  |  |  |  |  |  |  |
| KRT5 | 1.27 | 2.41 | <0.001 | 0.001 |  |  |  |  |  |  |  |
| ZMYND15 | 1.27 | 2.41 | <0.001 | <0.001 |  |  |  |  |  |  |  |
| ADAM28 | 1.27 | 2.41 | <0.001 | <0.001 |  |  |  |  |  |  |  |
| KYNU | 1.27 | 2.41 | <0.001 | <0.001 |  |  |  |  |  |  |  |
| PIK3R5 | 1.27 | 2.41 | <0.001 | <0.001 |  |  |  |  |  |  |  |
| TNFAIP6 | 1.27 | 2.41 | <0.001 | <0.001 |  |  |  |  |  |  |  |
| PTCHD4 | 1.27 | 2.41 | <0.001 | <0.001 |  |  |  |  |  |  |  |
| CHST11 | 1.27 | 2.41 | <0.001 | <0.001 |  |  |  |  |  |  |  |
| ITGA4 | 1.27 | 2.41 | <0.001 | <0.001 |  |  |  |  |  |  |  |
| HPSE | 1.27 | 2.41 | <0.001 | <0.001 |  |  |  |  |  |  |  |
| GM2AP1 | 1.26 | 2.39 | <0.001 | 0.003 |  |  |  |  |  |  |  |
| IDO1 | 1.26 | 2.39 | <0.001 | 0.007 |  |  |  |  |  |  |  |
| LINC00993 | 1.26 | 2.39 | <0.001 | 0.046 |  |  |  |  |  |  |  |
| CES1 | 1.26 | 2.39 | <0.001 | <0.001 |  |  |  |  |  |  |  |
| DAPP1 | 1.26 | 2.39 | <0.001 | <0.001 |  |  |  |  |  |  |  |
| RTN4R | 1.26 | 2.39 | <0.001 | <0.001 |  |  |  |  |  |  |  |
| KCNJ5 | 1.26 | 2.39 | <0.001 | <0.001 |  |  |  |  |  |  |  |
| ATF3 | 1.26 | 2.39 | <0.001 | <0.001 |  |  |  |  |  |  |  |
| SFRP4 | 1.25 | 2.38 | <0.001 | 0.002 |  |  |  |  |  |  |  |
| CDCA2 | 1.25 | 2.38 | <0.001 | 0.007 |  |  |  |  |  |  |  |
| NALCN | 1.25 | 2.38 | <0.001 | 0.032 |  |  |  |  |  |  |  |
| SCX | 1.25 | 2.38 | <0.001 | <0.001 |  |  |  |  |  |  |  |
| LILRB4 | 1.25 | 2.38 | <0.001 | <0.001 |  |  |  |  |  |  |  |
| CLDN18 | 1.25 | 2.38 | <0.001 | <0.001 |  |  |  |  |  |  |  |
| CCDC162P | 1.24 | 2.36 | <0.001 | 0.004 |  |  |  |  |  |  |  |
| TNFRSF9 | 1.24 | 2.36 | <0.001 | 0.009 |  |  |  |  |  |  |  |
| RNASE2 | 1.24 | 2.36 | <0.001 | 0.036 |  |  |  |  |  |  |  |
| BCL2A1 | 1.24 | 2.36 | <0.001 | <0.001 |  |  |  |  |  |  |  |
| GALNT6 | 1.24 | 2.36 | <0.001 | <0.001 |  |  |  |  |  |  |  |
| CXCL16 | 1.24 | 2.36 | <0.001 | <0.001 |  |  |  |  |  |  |  |
| NOS1AP | 1.24 | 2.36 | <0.001 | <0.001 |  |  |  |  |  |  |  |
| NRP2 | 1.24 | 2.36 | <0.001 | <0.001 |  |  |  |  |  |  |  |
| OTX1 | 1.23 | 2.35 | <0.001 | 0.006 |  |  |  |  |  |  |  |
| POPDC3 | 1.23 | 2.35 | <0.001 | 0.036 |  |  |  |  |  |  |  |
| SLC7A4 | 1.23 | 2.35 | <0.001 | <0.001 |  |  |  |  |  |  |  |
| CCR1 | 1.23 | 2.35 | <0.001 | <0.001 |  |  |  |  |  |  |  |
| ADCY7 | 1.23 | 2.35 | <0.001 | <0.001 |  |  |  |  |  |  |  |
| NFAM1 | 1.23 | 2.35 | <0.001 | <0.001 |  |  |  |  |  |  |  |
| DNM1P51 | 1.22 | 2.33 | <0.001 | 0.001 |  |  |  |  |  |  |  |
| SALL1 | 1.22 | 2.33 | <0.001 | 0.014 |  |  |  |  |  |  |  |
| GRIN2D | 1.22 | 2.33 | <0.001 | 0.014 |  |  |  |  |  |  |  |
| TDRD9 | 1.22 | 2.33 | <0.001 | 0.015 |  |  |  |  |  |  |  |
| LCNL1 | 1.22 | 2.33 | <0.001 | 0.018 |  |  |  |  |  |  |  |
| BPIFB2 | 1.22 | 2.33 | <0.001 | 0.020 |  |  |  |  |  |  |  |
| CARD9 | 1.22 | 2.33 | <0.001 | <0.001 |  |  |  |  |  |  |  |
| CXCL3 | 1.22 | 2.33 | <0.001 | <0.001 |  |  |  |  |  |  |  |
| ALPL | 1.21 | 2.31 | <0.001 | 0.001 |  |  |  |  |  |  |  |
| SLC4A10 | 1.21 | 2.31 | <0.001 | 0.008 |  |  |  |  |  |  |  |
| EPCAM | 1.21 | 2.31 | <0.001 | 0.009 |  |  |  |  |  |  |  |
| ABHD11-AS1 | 1.21 | 2.31 | <0.001 | 0.030 |  |  |  |  |  |  |  |
| SMTNL1 | 1.21 | 2.31 | <0.001 | 0.043 |  |  |  |  |  |  |  |
| GPR183 | 1.21 | 2.31 | <0.001 | <0.001 |  |  |  |  |  |  |  |
| CD84 | 1.21 | 2.31 | <0.001 | <0.001 |  |  |  |  |  |  |  |
| TNFRSF11B | 1.21 | 2.31 | <0.001 | <0.001 |  |  |  |  |  |  |  |
| F2R | 1.21 | 2.31 | <0.001 | <0.001 |  |  |  |  |  |  |  |
| CD180 | 1.21 | 2.31 | <0.001 | <0.001 |  |  |  |  |  |  |  |
| LINC01426 | 1.21 | 2.31 | <0.001 | <0.001 |  |  |  |  |  |  |  |
| ZNF469 | 1.21 | 2.31 | <0.001 | <0.001 |  |  |  |  |  |  |  |
| IL10 | 1.2 | 2.30 | <0.001 | 0.001 |  |  |  |  |  |  |  |
| CCDC144B | 1.2 | 2.30 | <0.001 | 0.002 |  |  |  |  |  |  |  |
| UHRF1 | 1.2 | 2.30 | <0.001 | 0.004 |  |  |  |  |  |  |  |
| CILP2 | 1.2 | 2.30 | <0.001 | 0.011 |  |  |  |  |  |  |  |
| C9orf106 | 1.2 | 2.30 | <0.001 | 0.012 |  |  |  |  |  |  |  |
| HORMAD2 | 1.2 | 2.30 | <0.001 | 0.027 |  |  |  |  |  |  |  |
| HK3 | 1.2 | 2.30 | <0.001 | <0.001 |  |  |  |  |  |  |  |
| PLEKHM3 | 1.2 | 2.30 | <0.001 | <0.001 |  |  |  |  |  |  |  |
| RTKN2 | 1.2 | 2.30 | <0.001 | <0.001 |  |  |  |  |  |  |  |
| KIF23 | 1.19 | 2.28 | <0.001 | 0.001 |  |  |  |  |  |  |  |
| CXCR3 | 1.19 | 2.28 | <0.001 | 0.002 |  |  |  |  |  |  |  |
| SHANK1 | 1.19 | 2.28 | <0.001 | 0.006 |  |  |  |  |  |  |  |
| SDC1 | 1.19 | 2.28 | <0.001 | 0.006 |  |  |  |  |  |  |  |
| RNF39 | 1.19 | 2.28 | <0.001 | 0.010 |  |  |  |  |  |  |  |
| KIF4A | 1.19 | 2.28 | <0.001 | 0.025 |  |  |  |  |  |  |  |
| RAPH1 | 1.19 | 2.28 | <0.001 | <0.001 |  |  |  |  |  |  |  |
| CCND1 | 1.19 | 2.28 | <0.001 | <0.001 |  |  |  |  |  |  |  |
| FCHO1 | 1.19 | 2.28 | <0.001 | <0.001 |  |  |  |  |  |  |  |
| NRXN3 | 1.19 | 2.28 | <0.001 | <0.001 |  |  |  |  |  |  |  |
| PEG10 | 1.18 | 2.27 | <0.001 | 0.001 |  |  |  |  |  |  |  |
| ANGPTL6 | 1.18 | 2.27 | <0.001 | 0.001 |  |  |  |  |  |  |  |
| AQP9 | 1.18 | 2.27 | <0.001 | 0.015 |  |  |  |  |  |  |  |
| CUX2 | 1.18 | 2.27 | <0.001 | 0.017 |  |  |  |  |  |  |  |
| HSPG2 | 1.18 | 2.27 | <0.001 | <0.001 |  |  |  |  |  |  |  |
| KCNB1 | 1.18 | 2.27 | <0.001 | <0.001 |  |  |  |  |  |  |  |
| PARVG | 1.18 | 2.27 | <0.001 | <0.001 |  |  |  |  |  |  |  |
| ANKDD1A | 1.18 | 2.27 | <0.001 | <0.001 |  |  |  |  |  |  |  |
| TNRC18P1 | 1.18 | 2.27 | <0.001 | <0.001 |  |  |  |  |  |  |  |
| FGF11 | 1.17 | 2.25 | <0.001 | 0.002 |  |  |  |  |  |  |  |
| E2F2 | 1.17 | 2.25 | <0.001 | 0.005 |  |  |  |  |  |  |  |
| CXorf65 | 1.17 | 2.25 | <0.001 | 0.018 |  |  |  |  |  |  |  |
| C5AR1 | 1.17 | 2.25 | <0.001 | <0.001 |  |  |  |  |  |  |  |
| ASGR1 | 1.17 | 2.25 | <0.001 | <0.001 |  |  |  |  |  |  |  |
| AMPD3 | 1.17 | 2.25 | <0.001 | <0.001 |  |  |  |  |  |  |  |
| CD1D | 1.17 | 2.25 | <0.001 | <0.001 |  |  |  |  |  |  |  |
| CGNL1 | 1.17 | 2.25 | <0.001 | <0.001 |  |  |  |  |  |  |  |
| DBNDD1 | 1.17 | 2.25 | <0.001 | <0.001 |  |  |  |  |  |  |  |
| NLRC4 | 1.17 | 2.25 | <0.001 | <0.001 |  |  |  |  |  |  |  |
| RAB3C | 1.17 | 2.25 | <0.001 | <0.001 |  |  |  |  |  |  |  |
| SEZ6L | 1.16 | 2.23 | <0.001 | 0.018 |  |  |  |  |  |  |  |
| ECEL1 | 1.16 | 2.23 | <0.001 | 0.026 |  |  |  |  |  |  |  |
| CTSS | 1.16 | 2.23 | <0.001 | <0.001 |  |  |  |  |  |  |  |
| ZMIZ1-AS1 | 1.16 | 2.23 | <0.001 | <0.001 |  |  |  |  |  |  |  |
| NCKAP1L | 1.16 | 2.23 | <0.001 | <0.001 |  |  |  |  |  |  |  |
| RAB39A | 1.16 | 2.23 | <0.001 | <0.001 |  |  |  |  |  |  |  |
| LGALS9 | 1.16 | 2.23 | <0.001 | <0.001 |  |  |  |  |  |  |  |
| COL12A1 | 1.16 | 2.23 | <0.001 | <0.001 |  |  |  |  |  |  |  |
| KIAA1522 | 1.16 | 2.23 | <0.001 | <0.001 |  |  |  |  |  |  |  |
| GPR17 | 1.15 | 2.22 | <0.001 | 0.001 |  |  |  |  |  |  |  |
| DIO2 | 1.15 | 2.22 | <0.001 | 0.003 |  |  |  |  |  |  |  |
| DTHD1 | 1.15 | 2.22 | <0.001 | 0.005 |  |  |  |  |  |  |  |
| SIGLEC5 | 1.15 | 2.22 | <0.001 | 0.029 |  |  |  |  |  |  |  |
| LINC00677 | 1.15 | 2.22 | <0.001 | 0.030 |  |  |  |  |  |  |  |
| PPP2R2C | 1.15 | 2.22 | <0.001 | 0.033 |  |  |  |  |  |  |  |
| PTGFR | 1.15 | 2.22 | <0.001 | <0.001 |  |  |  |  |  |  |  |
| KCNH3 | 1.15 | 2.22 | <0.001 | <0.001 |  |  |  |  |  |  |  |
| AMICA1 | 1.15 | 2.22 | <0.001 | <0.001 |  |  |  |  |  |  |  |
| LILRB1 | 1.15 | 2.22 | <0.001 | <0.001 |  |  |  |  |  |  |  |
| AP1S3 | 1.15 | 2.22 | <0.001 | <0.001 |  |  |  |  |  |  |  |
| STAC3 | 1.15 | 2.22 | <0.001 | <0.001 |  |  |  |  |  |  |  |
| NRIP3 | 1.14 | 2.20 | <0.001 | 0.002 |  |  |  |  |  |  |  |
| OAS3 | 1.14 | 2.20 | <0.001 | 0.003 |  |  |  |  |  |  |  |
| PTPRJ | 1.14 | 2.20 | <0.001 | <0.001 |  |  |  |  |  |  |  |
| GCNT2 | 1.14 | 2.20 | <0.001 | <0.001 |  |  |  |  |  |  |  |
| PTGIR | 1.14 | 2.20 | <0.001 | <0.001 |  |  |  |  |  |  |  |
| CD86 | 1.14 | 2.20 | <0.001 | <0.001 |  |  |  |  |  |  |  |
| TFEC | 1.14 | 2.20 | <0.001 | <0.001 |  |  |  |  |  |  |  |
| GRIP1 | 1.13 | 2.19 | <0.001 | 0.001 |  |  |  |  |  |  |  |
| CSF2RA | 1.13 | 2.19 | <0.001 | 0.001 |  |  |  |  |  |  |  |
| GAPT | 1.13 | 2.19 | <0.001 | 0.002 |  |  |  |  |  |  |  |
| RASL11B | 1.13 | 2.19 | <0.001 | 0.032 |  |  |  |  |  |  |  |
| FCRL5 | 1.13 | 2.19 | <0.001 | 0.032 |  |  |  |  |  |  |  |
| PIK3R6 | 1.13 | 2.19 | <0.001 | <0.001 |  |  |  |  |  |  |  |
| TNFRSF12A | 1.13 | 2.19 | <0.001 | <0.001 |  |  |  |  |  |  |  |
| PTPRC | 1.13 | 2.19 | <0.001 | <0.001 |  |  |  |  |  |  |  |
| FYB | 1.13 | 2.19 | <0.001 | <0.001 |  |  |  |  |  |  |  |
| SLCO2B1 | 1.13 | 2.19 | <0.001 | <0.001 |  |  |  |  |  |  |  |
| PLCB2 | 1.13 | 2.19 | <0.001 | <0.001 |  |  |  |  |  |  |  |
| GPAT2 | 1.12 | 2.17 | <0.001 | 0.001 |  |  |  |  |  |  |  |
| TRPM3 | 1.12 | 2.17 | <0.001 | 0.001 |  |  |  |  |  |  |  |
| ZBED6 | 1.12 | 2.17 | <0.001 | 0.007 |  |  |  |  |  |  |  |
| SLC46A2 | 1.12 | 2.17 | <0.001 | 0.016 |  |  |  |  |  |  |  |
| CFH | 1.12 | 2.17 | <0.001 | <0.001 |  |  |  |  |  |  |  |
| ZDHHC20P4 | 1.12 | 2.17 | <0.001 | <0.001 |  |  |  |  |  |  |  |
| HLA-DPA1 | 1.12 | 2.17 | <0.001 | <0.001 |  |  |  |  |  |  |  |
| RCC2P7 | 1.11 | 2.16 | <0.001 | 0.001 |  |  |  |  |  |  |  |
| PCDHAC2 | 1.11 | 2.16 | <0.001 | 0.001 |  |  |  |  |  |  |  |
| DCC | 1.11 | 2.16 | <0.001 | 0.008 |  |  |  |  |  |  |  |
| RPS4XP5 | 1.11 | 2.16 | <0.001 | 0.018 |  |  |  |  |  |  |  |
| PIK3AP1 | 1.11 | 2.16 | <0.001 | <0.001 |  |  |  |  |  |  |  |
| NFIX | 1.11 | 2.16 | <0.001 | <0.001 |  |  |  |  |  |  |  |
| TPTEP1 | 1.11 | 2.16 | <0.001 | <0.001 |  |  |  |  |  |  |  |
| SAMHD1 | 1.11 | 2.16 | <0.001 | <0.001 |  |  |  |  |  |  |  |
| SLC37A2 | 1.11 | 2.16 | <0.001 | <0.001 |  |  |  |  |  |  |  |
| BGN | 1.11 | 2.16 | <0.001 | <0.001 |  |  |  |  |  |  |  |
| CYP7B1 | 1.11 | 2.16 | <0.001 | <0.001 |  |  |  |  |  |  |  |
| FMNL1 | 1.11 | 2.16 | <0.001 | <0.001 |  |  |  |  |  |  |  |
| TNXB | 1.1 | 2.14 | <0.001 | 0.005 |  |  |  |  |  |  |  |
| DPYSL4 | 1.1 | 2.14 | <0.001 | 0.028 |  |  |  |  |  |  |  |
| CSF2RB | 1.1 | 2.14 | <0.001 | <0.001 |  |  |  |  |  |  |  |
| ITIH5 | 1.1 | 2.14 | <0.001 | <0.001 |  |  |  |  |  |  |  |
| FAM83H | 1.1 | 2.14 | <0.001 | <0.001 |  |  |  |  |  |  |  |
| RPS6KA1 | 1.1 | 2.14 | <0.001 | <0.001 |  |  |  |  |  |  |  |
| DOCK3 | 1.09 | 2.13 | <0.001 | 0.001 |  |  |  |  |  |  |  |
| LMOD1 | 1.09 | 2.13 | <0.001 | 0.001 |  |  |  |  |  |  |  |
| FCER1A | 1.09 | 2.13 | <0.001 | 0.002 |  |  |  |  |  |  |  |
| RPS24P6 | 1.09 | 2.13 | <0.001 | 0.039 |  |  |  |  |  |  |  |
| IQGAP2 | 1.09 | 2.13 | <0.001 | <0.001 |  |  |  |  |  |  |  |
| C10orf90 | 1.09 | 2.13 | <0.001 | <0.001 |  |  |  |  |  |  |  |
| HLA-DMB | 1.09 | 2.13 | <0.001 | <0.001 |  |  |  |  |  |  |  |
| VENTX | 1.09 | 2.13 | <0.001 | <0.001 |  |  |  |  |  |  |  |
| GPLD1 | 1.09 | 2.13 | <0.001 | <0.001 |  |  |  |  |  |  |  |
| PTPN7 | 1.09 | 2.13 | <0.001 | <0.001 |  |  |  |  |  |  |  |
| KCP | 1.09 | 2.13 | <0.001 | <0.001 |  |  |  |  |  |  |  |
| HLA-DRB6 | 1.08 | 2.11 | <0.001 | 0.002 |  |  |  |  |  |  |  |
| NOTCH3 | 1.08 | 2.11 | <0.001 | <0.001 |  |  |  |  |  |  |  |
| C2 | 1.08 | 2.11 | <0.001 | <0.001 |  |  |  |  |  |  |  |
| MMP24 | 1.08 | 2.11 | <0.001 | <0.001 |  |  |  |  |  |  |  |
| PHLDA1 | 1.08 | 2.11 | <0.001 | <0.001 |  |  |  |  |  |  |  |
| ITGAM | 1.08 | 2.11 | <0.001 | <0.001 |  |  |  |  |  |  |  |
| SEMA6B | 1.08 | 2.11 | <0.001 | <0.001 |  |  |  |  |  |  |  |
| BTK | 1.08 | 2.11 | <0.001 | <0.001 |  |  |  |  |  |  |  |
| THEMIS2 | 1.08 | 2.11 | <0.001 | <0.001 |  |  |  |  |  |  |  |
| SERPINA1 | 1.07 | 2.10 | <0.001 | 0.001 |  |  |  |  |  |  |  |
| CPNE5 | 1.07 | 2.10 | <0.001 | 0.001 |  |  |  |  |  |  |  |
| LRRC43 | 1.07 | 2.10 | <0.001 | 0.001 |  |  |  |  |  |  |  |
| KCNK15 | 1.07 | 2.10 | <0.001 | 0.002 |  |  |  |  |  |  |  |
| NCAPG | 1.07 | 2.10 | <0.001 | 0.009 |  |  |  |  |  |  |  |
| NPTX2 | 1.07 | 2.10 | <0.001 | 0.022 |  |  |  |  |  |  |  |
| KCNA2 | 1.07 | 2.10 | <0.001 | 0.040 |  |  |  |  |  |  |  |
| FCAR | 1.07 | 2.10 | <0.001 | 0.053 |  |  |  |  |  |  |  |
| IRF8 | 1.07 | 2.10 | <0.001 | <0.001 |  |  |  |  |  |  |  |
| POLR2A | 1.07 | 2.10 | <0.001 | <0.001 |  |  |  |  |  |  |  |
| CD74 | 1.07 | 2.10 | <0.001 | <0.001 |  |  |  |  |  |  |  |
| GMIP | 1.07 | 2.10 | <0.001 | <0.001 |  |  |  |  |  |  |  |
| C1orf233 | 1.07 | 2.10 | <0.001 | <0.001 |  |  |  |  |  |  |  |
| STC1 | 1.07 | 2.10 | <0.001 | <0.001 |  |  |  |  |  |  |  |
| AGRN | 1.07 | 2.10 | <0.001 | <0.001 |  |  |  |  |  |  |  |
| IL2RG | 1.07 | 2.10 | <0.001 | <0.001 |  |  |  |  |  |  |  |
| EPHB1 | 1.06 | 2.08 | <0.001 | 0.001 |  |  |  |  |  |  |  |
| IL24 | 1.06 | 2.08 | <0.001 | 0.006 |  |  |  |  |  |  |  |
| OTOA | 1.06 | 2.08 | <0.001 | 0.011 |  |  |  |  |  |  |  |
| ESPL1 | 1.06 | 2.08 | <0.001 | 0.015 |  |  |  |  |  |  |  |
| RELT | 1.06 | 2.08 | <0.001 | <0.001 |  |  |  |  |  |  |  |
| MS4A7 | 1.06 | 2.08 | <0.001 | <0.001 |  |  |  |  |  |  |  |
| ID4 | 1.06 | 2.08 | <0.001 | <0.001 |  |  |  |  |  |  |  |
| LILRB3 | 1.06 | 2.08 | <0.001 | <0.001 |  |  |  |  |  |  |  |
| FERMT3 | 1.06 | 2.08 | <0.001 | <0.001 |  |  |  |  |  |  |  |
| MYO1G | 1.06 | 2.08 | <0.001 | <0.001 |  |  |  |  |  |  |  |
| CARD11 | 1.06 | 2.08 | <0.001 | <0.001 |  |  |  |  |  |  |  |
| NPY1R | 1.06 | 2.08 | <0.001 | <0.001 |  |  |  |  |  |  |  |
| VGLL3 | 1.06 | 2.08 | <0.001 | <0.001 |  |  |  |  |  |  |  |
| C19orf35 | 1.05 | 2.07 | <0.001 | 0.001 |  |  |  |  |  |  |  |
| LINGO3 | 1.05 | 2.07 | <0.001 | 0.001 |  |  |  |  |  |  |  |
| PKP1 | 1.05 | 2.07 | <0.001 | 0.006 |  |  |  |  |  |  |  |
| SAMD5 | 1.05 | 2.07 | <0.001 | 0.006 |  |  |  |  |  |  |  |
| ARRDC5 | 1.05 | 2.07 | <0.001 | 0.025 |  |  |  |  |  |  |  |
| TLDC2 | 1.05 | 2.07 | <0.001 | <0.001 |  |  |  |  |  |  |  |
| LEP | 1.05 | 2.07 | <0.001 | <0.001 |  |  |  |  |  |  |  |
| IGSF6 | 1.05 | 2.07 | <0.001 | <0.001 |  |  |  |  |  |  |  |
| FCGR3A | 1.05 | 2.07 | <0.001 | <0.001 |  |  |  |  |  |  |  |
| SOWAHD | 1.05 | 2.07 | <0.001 | <0.001 |  |  |  |  |  |  |  |
| PALLD | 1.05 | 2.07 | <0.001 | <0.001 |  |  |  |  |  |  |  |
| HIST1H2BC | 1.04 | 2.06 | <0.001 | 0.001 |  |  |  |  |  |  |  |
| VCAN | 1.04 | 2.06 | <0.001 | 0.001 |  |  |  |  |  |  |  |
| NAPSB | 1.04 | 2.06 | <0.001 | 0.005 |  |  |  |  |  |  |  |
| GPR68 | 1.04 | 2.06 | <0.001 | 0.015 |  |  |  |  |  |  |  |
| CCDC88B | 1.04 | 2.06 | <0.001 | <0.001 |  |  |  |  |  |  |  |
| SASH3 | 1.04 | 2.06 | <0.001 | <0.001 |  |  |  |  |  |  |  |
| CD101 | 1.04 | 2.06 | <0.001 | <0.001 |  |  |  |  |  |  |  |
| PTAFR | 1.04 | 2.06 | <0.001 | <0.001 |  |  |  |  |  |  |  |
| ARHGAP30 | 1.04 | 2.06 | <0.001 | <0.001 |  |  |  |  |  |  |  |
| MSNP1 | 1.04 | 2.06 | <0.001 | <0.001 |  |  |  |  |  |  |  |
| SLIT2 | 1.04 | 2.06 | <0.001 | <0.001 |  |  |  |  |  |  |  |
| PLBD2 | 1.04 | 2.06 | <0.001 | <0.001 |  |  |  |  |  |  |  |
| P2RX7 | 1.04 | 2.06 | <0.001 | <0.001 |  |  |  |  |  |  |  |
| PEMT | 1.04 | 2.06 | <0.001 | <0.001 |  |  |  |  |  |  |  |
| LINC01106 | 1.03 | 2.04 | <0.001 | 0.018 |  |  |  |  |  |  |  |
| CSMD2 | 1.03 | 2.04 | <0.001 | 0.030 |  |  |  |  |  |  |  |
| MPO | 1.03 | 2.04 | <0.001 | 0.042 |  |  |  |  |  |  |  |
| EGR3 | 1.03 | 2.04 | <0.001 | <0.001 |  |  |  |  |  |  |  |
| HLA-DPB1 | 1.03 | 2.04 | <0.001 | <0.001 |  |  |  |  |  |  |  |
| FCGR2A | 1.03 | 2.04 | <0.001 | <0.001 |  |  |  |  |  |  |  |
| VLDLR | 1.03 | 2.04 | <0.001 | <0.001 |  |  |  |  |  |  |  |
| DOK5 | 1.03 | 2.04 | <0.001 | <0.001 |  |  |  |  |  |  |  |
| CXCR2P1 | 1.02 | 2.03 | <0.001 | 0.010 |  |  |  |  |  |  |  |
| CROCC2 | 1.02 | 2.03 | <0.001 | 0.012 |  |  |  |  |  |  |  |
| EPHX3 | 1.02 | 2.03 | <0.001 | 0.022 |  |  |  |  |  |  |  |
| TMEM139 | 1.02 | 2.03 | <0.001 | 0.047 |  |  |  |  |  |  |  |
| OSBPL3 | 1.02 | 2.03 | <0.001 | <0.001 |  |  |  |  |  |  |  |
| MAP4K1 | 1.02 | 2.03 | <0.001 | <0.001 |  |  |  |  |  |  |  |
| STXBP2 | 1.02 | 2.03 | <0.001 | <0.001 |  |  |  |  |  |  |  |
| DOCK2 | 1.02 | 2.03 | <0.001 | <0.001 |  |  |  |  |  |  |  |
| SYT12 | 1.02 | 2.03 | <0.001 | <0.001 |  |  |  |  |  |  |  |
| CD248 | 1.02 | 2.03 | <0.001 | <0.001 |  |  |  |  |  |  |  |
| FBXO41 | 1.02 | 2.03 | <0.001 | <0.001 |  |  |  |  |  |  |  |
| GAS6-AS1 | 1.02 | 2.03 | <0.001 | <0.001 |  |  |  |  |  |  |  |
| EOMES | 1.01 | 2.01 | <0.001 | 0.002 |  |  |  |  |  |  |  |
| PMFBP1 | 1.01 | 2.01 | <0.001 | 0.003 |  |  |  |  |  |  |  |
| FBXL16 | 1.01 | 2.01 | <0.001 | 0.004 |  |  |  |  |  |  |  |
| DNAH2 | 1.01 | 2.01 | <0.001 | 0.004 |  |  |  |  |  |  |  |
| FAM225B | 1.01 | 2.01 | <0.001 | 0.006 |  |  |  |  |  |  |  |
| SPAG17 | 1.01 | 2.01 | <0.001 | 0.015 |  |  |  |  |  |  |  |
| VSTM2L | 1.01 | 2.01 | <0.001 | 0.026 |  |  |  |  |  |  |  |
| LINC00937 | 1.01 | 2.01 | <0.001 | 0.032 |  |  |  |  |  |  |  |
| IL6STP1 | 1.01 | 2.01 | <0.001 | <0.001 |  |  |  |  |  |  |  |
| LACE1 | 1.01 | 2.01 | <0.001 | <0.001 |  |  |  |  |  |  |  |
| LAPTM5 | 1.01 | 2.01 | <0.001 | <0.001 |  |  |  |  |  |  |  |
| GLB1 | 1.01 | 2.01 | <0.001 | <0.001 |  |  |  |  |  |  |  |
| GRM7 | 1.00 | 2.00 | 0.003 | 0.017 |  |  |  |  |  |  |  |
| CHST13 | 1.00 | 2.00 | 0.010 | 0.055 |  |  |  |  |  |  |  |
| MUC1 | 1.00 | 2.00 | <0.001 | 0.006 |  |  |  |  |  |  |  |
| TNS3 | 1.00 | 2.00 | <0.001 | <0.001 |  |  |  |  |  |  |  |
| DLEC1 | 1.00 | 2.00 | <0.001 | <0.001 |  |  |  |  |  |  |  |
| FGF1 | 1.00 | 2.00 | <0.001 | <0.001 |  |  |  |  |  |  |  |
| TRIM14 | 1.00 | 2.00 | <0.001 | <0.001 |  |  |  |  |  |  |  |
| IL18 | 1.00 | 2.00 | <0.001 | <0.001 |  |  |  |  |  |  |  |

FC, fold change; FDR, false discovery rate

Log2 FC and *P* values were obtained by comparison of data from individuals living with obesity vs data from normal weight individuals at study entry (week-0) in a general linear model likelihood ratio test in EdgeR software.
