# Supplementary material for: Modification of subcutaneous white adipose tissue inflammation by omega-3 fatty acids is limited in human obesity-a double blind, randomised clinical trial
Source: eBioMedicine. 2022 Mar 2;77:103909. doi: 10.1016/j.ebiom.2022.103909 (PMC8894262; doi:10.1016/j.ebiom.2022.103909)
Supplement: Supplementary file 6 [file mmc6.docx]

|  |  | Fish Oil | Corn Oil |
| --- | --- | --- | --- |
| Saturated fatty acids (g) | 16:00 | 0.04 | 0.11 |
|  | 18:00 | 0.04 | 0.02 |
|  | **Total SFA** | **0.08** | **0.12** |
|  |  |  |  |
| Monounsaturated (g) | 16:1n-7 | 0.01 | 0.00 |
|  | 18:1n-7 | 0.03 | 0.01 |
|  | **Total n-7** | **0.04** | **0.01** |
|  | 18:1n-9 | 0.08 | 0.27 |
|  | 20:1n-9 | 0.02 | 0.00 |
|  | **Total n-9** | **0.10** | **0.27** |
|  | **Total MUFA** | **0.14** | **0.28** |
|  |  |  |  |
| Polyunsaturated (g) | 18:2n-6 | 0.01 | 0.55 |
|  | 20:4n-6 | 0.02 | 0.00 |
|  | **Total n-6 PUFA** | **0.03** | **0.55** |
|  | 18:3n-3 | 0.02 | 0.01 |
|  | 20:4n-3 | 0.02 | 0.00 |
|  | 20:5n-3 | 0.36 | 0.00 |
|  | 22:5n-3 | 0.05 | 0.00 |
|  | 22:6n-3 | 0.26 | 0.00 |
|  | **Total n-3 PUFA** | **0.71** | **0.00** |
|  | **Total PUFA** | **0.77** | **0.56** |
| **Total FA** |  | **0.98** | **0.98** |
| g of fatty / g of oil |  |  |  |
|  |  |  |  |
