# Supplementary material for: Modification of subcutaneous white adipose tissue inflammation by omega-3 fatty acids is limited in human obesity-a double blind, randomised clinical trial
Source: eBioMedicine. 2022 Mar 2;77:103909. doi: 10.1016/j.ebiom.2022.103909 (PMC8894262; doi:10.1016/j.ebiom.2022.103909)
Supplement: Supplementary file 7 [file mmc7.docx]

| **Official Gene Symbol** | **Sequence Length** | **Sense Primer** | **Anti-sense Primer** | **Dye** |
| --- | --- | --- | --- | --- |
| ALOX5 | 2591 | 5' AAGCGATGGAGAACCTGTTCA | 3' GTCTTCCTGCCAGTGATTCATG | VIC |
| ALOX12 | 2358 | 5' CTCCTGGAACTGCCTSGAAGAC | 3' CTGGTAGCTGAACAACTCATCATC | FAM |
| ALOX15 | 2707 | 5' ATCTTCTGAGGGGACACTTGA | 3' GTATCGCAGGTGGGGAATTATA | FAM |
| GABARAP | 924 | 5' GCTCGGATAGGAGACCTGGA | 3' GCTCGGAGATGAATTCGCTTC | VIC |
| PTGS2 | 4507 | 5' CAAATCATCAACACTGCCTCAAT | 3' TCTGGATCTGGAACACTGAATG | FAM |
| SLC27A2 | 2408 | 5' TATGACCTGATTAAATATGATGTGGAG | 3' CATTAAATGGTGTAAGTTGTGTGATT | FAM |
| VPS36 | 4473 | 5' GGAGGTAGAXGGXTXAGGAA | 3' CCTGTCTGTGGCTTTGAGGTAA | VIC |
