# Supplementary material for: Modification of subcutaneous white adipose tissue inflammation by omega-3 fatty acids is limited in human obesity-a double blind, randomised clinical trial
Source: eBioMedicine. 2022 Mar 2;77:103909. doi: 10.1016/j.ebiom.2022.103909 (PMC8894262; doi:10.1016/j.ebiom.2022.103909)
Supplement: Supplementary file 8 [file mmc8.docx]

| **Gene** | **hgnc_symbol** | **Log FC** | **Log CPM** | **P value** | **FDR** |
| --- | --- | --- | --- | --- | --- |
| ENSG00000130234 | *ACE2* | -0.586 | 0.313 | 3.54E-02 | 0.114 |
