# Supplementary material for: Modification of subcutaneous white adipose tissue inflammation by omega-3 fatty acids is limited in human obesity-a double blind, randomised clinical trial
Source: eBioMedicine. 2022 Mar 2;77:103909. doi: 10.1016/j.ebiom.2022.103909 (PMC8894262; doi:10.1016/j.ebiom.2022.103909)
Supplement: Supplementary file 9 [file mmc9.docx]

| RBC EPA (%) Week-0 | | | RBC EPA (%) Week-12 | | |
| --- | --- | --- | --- | --- | --- |
| Normal weight | Obese | *P* | Normal weight | Obese | *P* |
| 0.80 (0.66, 1.09) | 1.15 (0.96, 1.24) | 0.005 | 3.21 (2.59, 4.09) | 3.01 (2.60, 3.27) | 0.214 |
|  |  |  |  |  |  |
| NEFA EPA (ug/mL) Week-0 | | | NEFA EPA (ug/mL) Week-12 | | |
| Normal weight | Obese | *P* | Normal weight | Obese | *P* |
| 0.40 (0.24, 0.77) | 0.32 (0.16, 0.53) | 0.367 | 1.13 (0.59, 1.55) | 0.48 (0.33, 1.00) | 0.036 |
|  |  |  |  |  |  |
| scWAT EPA (%) Week-0 | | | scWAT EPA (%) Week-12 | | |
| Normal weight | Obese | *P* | Normal weight | Obese | *P* |
| 0.08 (0.07, 0.11) | 0.10 (0.08, 0.12) | 0.141 | 0.14 (0.12, 0.17) | 0.16 (0.14, 0.22) | 0.149 |
|  |  |  |  |  |  |
| RBC DHA (%) Week-0 | | | RBC DHA (%) Week-12 | | |
| Normal weight | Obese | *P* | Normal weight | Obese | *P* |
| 4.23 (3.43, 5.25) | 4.63 (3.43, 5.55) | 0.513 | 6.20 (5.80, 6.46) | 6.15 (5.51, 6.63) | 0.989 |
|  |  |  |  |  |  |
| NEFA DHA (ug/mL) Week-0 | | | NEFA DHA (ug/mL) Week-12 | | |
| Normal weight | Obese | *P* | Normal weight | Obese | *P* |
| 1.46 (0.76, 1.99) | 1.29 (0.87, 1.76) | 0.497 | 2.69 (1.80, 3.75) | 1.67 (1.16, 2.07) | 0.008 |
|  |  |  |  |  |  |
| scWAT DHA (%) Week-0 | | | scWAT DHA (%) Week-12 | | |
| Normal weight | Obese | *P* | Normal weight | Obese | *P* |
| 0.16 (0.14, 0.21) | 0.19 (0.17, 0.26) | 0.373 | 0.22 (0.19, 0.29) | 0.25 (0.18, 0.32) | 0.940 |
